# A translatome-transcriptome multi-omics gene regulatory network reveals the complicated functional landscape of maize

**DOI:** 10.1186/s13059-023-02890-4

**Published:** 2023-03-29

**Authors:** Wanchao Zhu, Xinxin Miao, Jia Qian, Sijia Chen, Qixiao Jin, Mingzhu Li, Linqian Han, Wanshun Zhong, Dan Xie, Xiaoyang Shang, Lin Li

**Affiliations:** 1 National Key Laboratory of Crop Genetic Improvement, Huazhong Agricultural University, Wuhan, 430070 China; 2 HuBei HongShan Laboratory, Wuhan, 430070 China

**Keywords:** Maize, Translatome, Gene regulatory networks (GRNs), Transcription factor (TF)

## Abstract

**Background:**

Maize (*Zea mays* L.) is one of the most important crops worldwide. Although sophisticated maize gene regulatory networks (GRNs) have been constructed for functional genomics and phenotypic dissection, a multi-omics GRN connecting the translatome and transcriptome is lacking, hampering our understanding and exploration of the maize regulatome.

**Results:**

We collect spatio-temporal translatome and transcriptome data and systematically explore the landscape of gene transcription and translation across 33 tissues or developmental stages of maize. Using this comprehensive transcriptome and translatome atlas, we construct a multi-omics GRN integrating mRNAs and translated mRNAs, demonstrating that translatome-related GRNs outperform GRNs solely using transcriptomic data and inter-omics GRNs outperform intra-omics GRNs in most cases. With the aid of the multi-omics GRN, we reconcile some known regulatory networks. We identify a novel transcription factor, *ZmGRF6*, which is associated with growth. Furthermore, we characterize a function related to drought response for the classic transcription factor *ZmMYB31*.

**Conclusions:**

Our findings provide insights into spatio-temporal changes across maize development at both the transcriptome and translatome levels. Multi-omics GRNs represent a useful resource for dissection of the regulatory mechanisms underlying phenotypic variation.

**Supplementary Information:**

The online version contains supplementary material available at 10.1186/s13059-023-02890-4.

## Background

Maize (*Zea mays* L.) is not only one of most important food and energy crops, but also a model species for plant genetics. Maize and other crops need to constantly adjust their gene expression profiles to adapt to changing environments and various stresses [1, 2]. Characterizing the regulation of gene expression is helpful for understanding adaptive mechanisms in maize and for modern breeding.

Transcription factors (TFs) account for 6–8% of protein-coding genes in multicellular organisms and play pivotal roles in regulating gene expression [3]. TFs recognize specific *cis*-regulatory elements to control the transcription of target genes, representing the regulatome [4]. The sessile mode of life subjects plants to drastic variations in environment, leading to dramatic changes in gene status compared to those seen in animals [5, 6]. Therefore, the regulatome or gene regulatory networks (GRNs) of plants are inherently complicated [7, 8].

Although a variety of approaches have been used to construct GRNs, including chromatin immunoprecipitation sequencing (ChIP-seq), yeast one-hybrid screen (Y1H), and protein-binding microarrays (PBM), these assays remain insufficient for studying the regulatory landscape of plants owing to their complexity and low throughput [9–11]. Exploiting the massive quantities of mRNA data now are available, GRNs linking TFs to their targets can be inferred using statistical algorithms and machine learning techniques based on gene expression data [12, 13]. These GRN inference approaches are effective tools for identifying genes that have important biological functions or participate in specific pathways [6, 7]. For example, characterization of key genes and their regulatory relationships at the transcript level using GRNs has revealed important roles in cell wall biosynthesis [14], regeneration [15], and root hair growth [16].

Pilot studies on GRN construction in maize have made great strides, providing substantial insight into the maize regulatome. A maize network was constructed using nine different endosperm, embryo, and kernel tissues, which illustrated the close correlation between the embryo and the aleurone layer of the endosperm [17]. Comprehensive GRNs have been established using large-scale transcriptome datasets (>6000 RNA sequencing samples) and showed that the presence/absence of TFs has greater effects on the expression of target genes than quantitative changes of TF expression [12]. However, these GRNs were constructed solely using transcriptome data.

Transcriptome datasets have been widely applied to infer genome-wide GRNs and investigate possible functional roles of individual genes at a system-wide scale based on the assumption that measured mRNA levels are a proxy for protein abundance [18]. In reality, mRNA levels are weakly correlated with protein abundance because multiple biological processes act on the intermediates between mRNAs and proteins, including mRNA degradation, translation, and protein folding [19]. Thus, GRNs built solely on transcriptome data are not as robust as once assumed. To enhance GRN predictions, integrating transcriptome and proteome data was employed to investigate regulatory relationships between TFs and their targets, improving the predictive power of GRNs of maize [18]. Nevertheless, in this example, only 17,862 unmodified proteins were detected across 33 maize tissues, and only 545 TFs with detectable protein abundance could be used to construct an independent GRN, thus limiting the thorough exploration of the complete regulatome [18].

The translatome, a middle layer between the transcriptome and the proteome, has received increasing attention recently because of its status as a more effective proxy for the proteome than the transcriptome [20]. Translatome research has been used to investigate multiple physiological processes in plants. In maize, the translatome and transcriptome landscapes of seedlings showed that drought stress results in independent transcriptional and translational responses [21]. The translatome and transcriptome of maize leaves at the V4 stage revealed translational fractionation of maize subgenome genes potentially associated with heterosis [22]. Given the relatively low correlation between the transcriptome and proteome and the fact that the translatome represents a better proxy for the proteome, an inter-omic GRN between the translatome (for TFs) and the transcriptome (for targets) might better characterize the underlying molecular regulatory machinery of maize.

In this study, we therefore compiled a comprehensive translatomic and transcriptomic database spanning most of the maize life cycle. Combining a spatiotemporally distributed translatome with transcriptome datasets, we constructed multiple GRNs at different omic levels to investigate the regulatome of maize (Additional file 1: Fig. S1). Translatome-related GRNs showed better performance than GRNs considering solely the transcriptome. We used the union GRN to verify known regulatory networks for kernel development and leaf photosynthesis. We also identified a previously unreported TF (*ZmGRF6*) associated with growth and defined an uncharacterized function of the TF *ZmMYB31* related to drought response. Our multi-omics GRN represents a comprehensive regulatome of maize and underscores the complex functional landscape of plants.

## Results

### Comprehensive transcriptome and translatome data across maize development

**Fig. 1:**
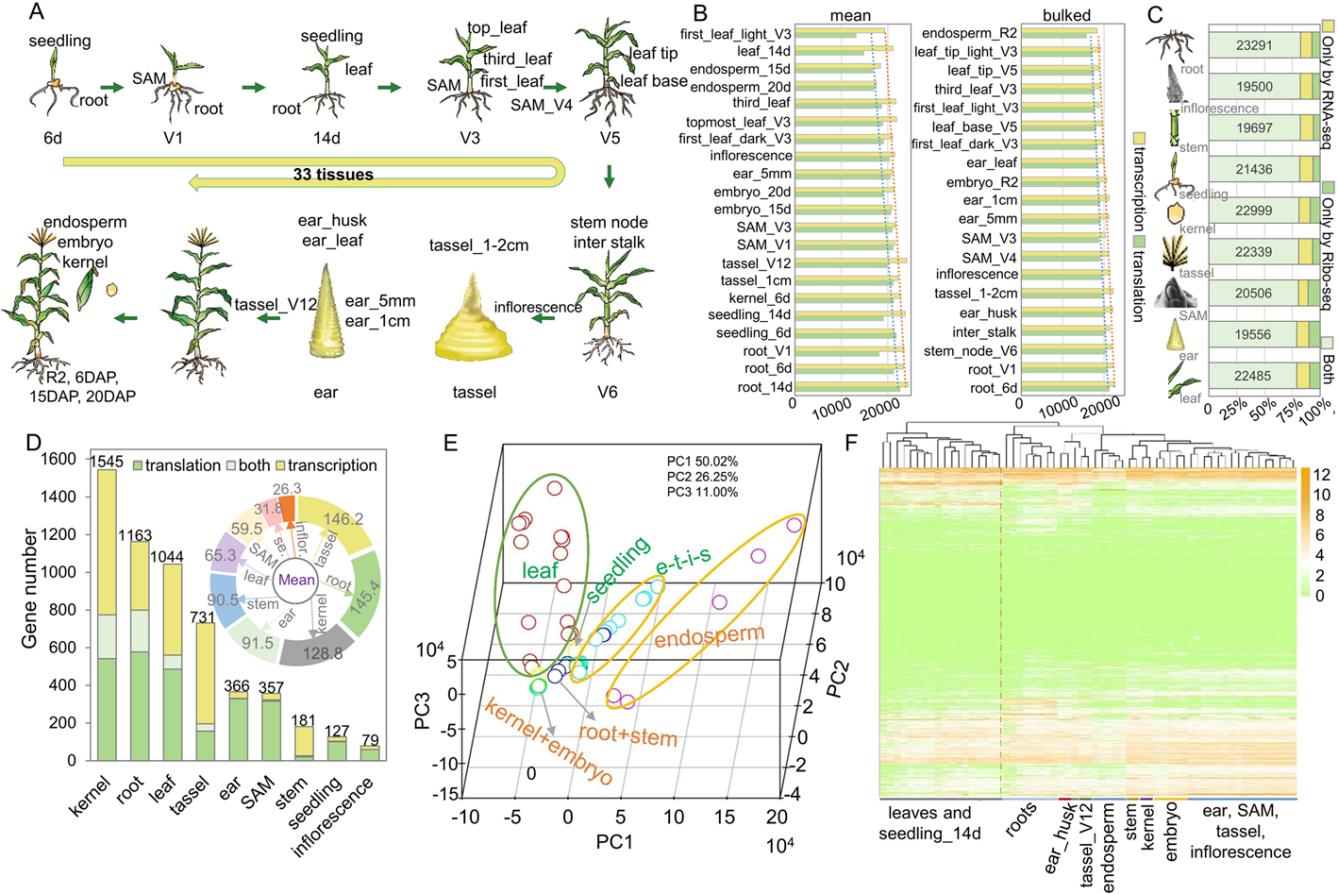
Overview of the samples and datasets used in this study. **A** Samples collected across maize development. **B** Numbers of translated or transcribed genes detected across all samples. Yellow, transcribed; green, translated. **C** Number of genes in different tissue categories detected by RNA-seq or Ribo-seq. **D** Bar plot, number of genes specifically transcribed (yellow), specifically translated (green), or both transcribed and translated (light green) in a single tissue type across different tissue categories; the circular diagram refers to the mean value of specifically expressed genes (SEGs) after dividing by the sample number in each tissue category. **E** Principal component analysis (PCA) results for the translatome data of all maize samples. The colored circles indicate the respective tissues/stages of maize development: red (leaf), green (kernel and embryo), yellow (seedling), purple (endosperm), light blue (e - ear, t - tassel, i - inflorescence and s - Shoot Apical Meristem), dark blue (root and stem). **F** Hierarchically clustered heatmap based on the translatome data from all maize samples

**Fig. 2:**
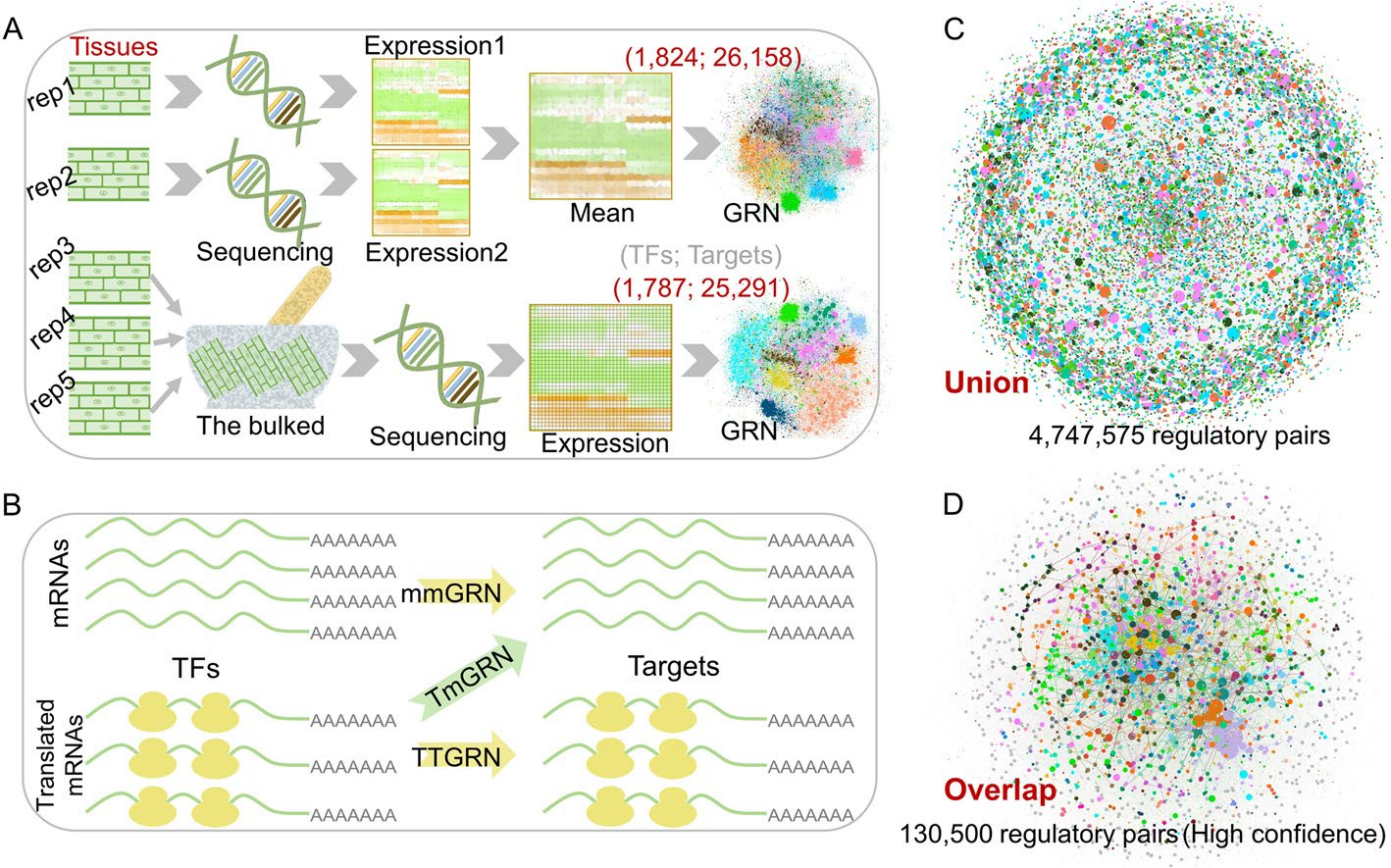
Genome-wide regulatory landscape of maize. **A** Construction of GRNs from two different data sources in maize. A mean GRN was constructed from the transcriptome and translatome of 21 tissues based on the mean expression levels from two biological replicates. A bulked GRN was constructed from the transcriptome and translatome of bulked samples from 20 tissues using the same methods, each bulked sample from three independent biological replicates. **B** Classification of different types of intra- and inter-omics GRNs. mmGRN and TTGRN represent intra-omics GRNs at the transcriptome and translatome levels, respectively, while TmGRN represents an inter-omics GRN between the translatome (TF) and transcriptome (Target). **C** Union GRN created by merging all GRNs (top 1 million edges), including both mean and bulked GRNs. **D** High-confidence network was generated by overlapping GRNs at network type level firstly (TT, TM, MM) and then merging the overlapped GRNs from different data sources (mean and bulked)

To create an integrated and dynamic transcriptome and translatome atlas across maize development, we collected comprehensive transcriptome and translatome data from 33 different tissues or developmental stages of the reference maize inbred line B73 (Fig. 1A, Table S1). The transcriptome and translatome of 21 tissues were profiled previously using RNA-seq and ribosome profiling (Ribo-seq), respectively, as two biological replicates [20, 23]. Here, we additionally obtained the transcriptome and translatome of bulk samples from 20 tissues (some tissues were identical to those described previously) using the same methods, each being a bulk from three independent biological replicates (each replicate contains three individual plants) (Fig. 2A). Transcriptome datasets across different tissues or stages exhibited high Pearson’s correlation coefficients, ranging from 0.880 to 0.963, between the two replicates and the bulked samples; translatome datasets had Pearson’s correlation coefficients ranging from 0.789 to 0.973 (Additional file 1: Fig. S2). Moreover, the dominant size of ribosome-imprinted RNA fragments (RPFs) ranged from 26 to 30 nucleotides (nt) in most samples, with a clear 3-nt periodicity near the start and stop codon sites of coding sequences (Additional file 1: Fig. S3 and Fig. S4), confirming the reliability of these translatome datasets. These results suggest that we had collected high-quality translatomic and transcriptomic data across maize developmental stages.

We then separately quantified transcript abundance from both the transcriptome and translatome datasets. In general, we detected more genes at the transcriptome level (Fig. 1B). In vegetative tissues, the roots and stems expressed more genes than leaves and endosperms at both the translatome and transcriptome levels (Fig. 1B, Additional file 1: Fig. S5A-5D). Reproductive tissues including ear, tassel, inflorescence, embryo, and kernel expressed more genes than leaves, but fewer than roots (Fig. 1B, Additional file 1: Fig. S5A-5D). We classified all samples into several major categories according to tissue type: root, inflorescence, stem, seedling, kernel, tassel, shoot apical meristem (SAM), ear, and leaf (Fig. 1C). Only a small proportion of genes could be specifically detected by a single technology (RNA-seq or Ribo-seq), while most expressed genes (78.97–82.43%) were detected by both omics technologies (Fig. 1C), suggesting that the transcripts from most transcribed genes are also actively translated. The number of genes specifically expressed in a single tissue type (specifically expressed genes, or SEGs, referring to the sum of specifically translated genes and specifically transcribed genes) in each category ranged from 79 to 1545, with most being detected at a single omics level (Fig. 1D, Additional file 1: Fig. S5E). Because the number of samples in each tissue category varied, we used the mean number of SEGs to evaluate tissue-specific expression, finding greater numbers of mean SEGs in most reproductive organs (tassel, kernel, and ear) and a few vegetative organs (root and stem) than in other tissues (Fig. 1D, Additional file 1: Fig. S5E-5F).

Principal component analysis (PCA) demonstrated that leaf and endosperm samples cluster across different developmental stages; however, the translatomic patterns of other tissues were ambiguous (Fig. 1E). To further characterize translatomic patterns of genes across different tissues, we performed a hierarchical clustering analysis using the translatome data. We observed the clustering of translated genes from almost green tissues including leaves and seedlings, while those translated in non-green tissues, including all other tissues, gathered into a second cluster (Fig. 1F). Additionally, the translatome and transcriptome of the same or similar tissues showed high correlation (Additional file 1: Fig. S6A), while correlation coefficients for the median abundance at the transcriptome and translatome levels across different tissues ranged from 0.829 to 0.837 (Additional file 1: Fig. S6B). The abundance of most genes at the two omics levels showed variable correlation coefficients ranging from 0.5 to 1 across different tissues (Additional file 1: Fig. S6C). Notably, a few genes exhibited very low or even negative correlation coefficients between the two omics levels. In addition, the translational efficiency (TE) of most detectable genes across all tissues ranged from 0.25 to 4, with TE of genes in green tissues likely exhibiting greater instability (Additional file 1: Fig. S6D). These results illustrate the generation of a complex transcriptome and translatome atlas in this study.

### Comprehensive regulatory landscape of maize inferred from multi-omics gene regulatory networks (GRNs)

Plant development and phenotypic variation are controlled by accurate and complex gene regulatory networks (GRNs) [6]. To construct a genome-wide regulatome of maize for dissecting the molecular mechanisms underlying complex traits, we generated a comprehensive transcriptome and translatome dataset using two different data sources (the two separately sequenced replicates-rep1 and rep2, the mean expression levels of which were used for the following analyses; bulked data from a sequencing library constructed from equivalent RNAs of three biological replicates) from 33 tissue or developmental stages (Fig. 2A). We collected information on all maize TFs from PlantTFDB (http://planttfdb.gao-lab.org/tf.php?sp=Ppe&did=Prupe.I004500.1.p). We set 1,824 and 1,787 TFs detectable at both the translatome and transcriptome levels as “regulators” in groups mean and bulked, respectively (Fig. 2A). Accordingly, 26,158 and 25,291 genes with detectable transcripts were set as potential “targets” across different groups (Fig. 2A). We constructed putative GRNs based upon the expression patterns of TF genes and the target genes of their encoded proteins for the two different datasets, representing three types of intra- and inter-GRNs across different datasets (Fig. 2B). Intra-omics GRNs at the transcriptome and translatome levels were named as mmGRNs (TF transcriptional level *vs*. target transcriptional level) and TTGRNs (TF translational level *vs*. target translational level), respectively, while inter-omics GRNs were named as TmGRNs (TF translational level *vs*. target transcriptional level) (Fig. 2B).

To characterize these intra- and inter-omics GRNs in maize, we employed network parameters such as transitivity, average path length, and module number to quantify the topological architecture of the different GRNs. TmGRNs showed the lowest transitivity, highest average.path.length, and fewest modules (nodes ≥5), but differences were marginal compared with mmGRNs and TTGRNs (Additional file 1: Fig. S7). Integration of different omics data sets can greatly improve the predictive power of GRNs [18]. We therefore merged all three types of GRNs (considering only the top one million edges from each GRN) from our two data groups to obtain 4,747,575 gene regulatory pairs (Fig. 2C; Additional file 13, which was also deposited at http://zeasystemsbio.hzau.edu.cn/dataset.html). We also used the overlap among the top one million edges of the mmGRNs, TTGRNs, and TmGRNs from each dataset to create a network comprising 130,500 high-confidence regulatory pairs (Fig. 2D; Additional file 14, which was also deposited at http://zeasystemsbio.hzau.edu.cn/dataset.html). These networks provide an unprecedented resource for a genome-wide dissection of the maize regulatome.

### TmGRN and TTGRN outperform mmGRN, and TmGRN shows the best performance in multiple scenarios

**Fig. 3:**
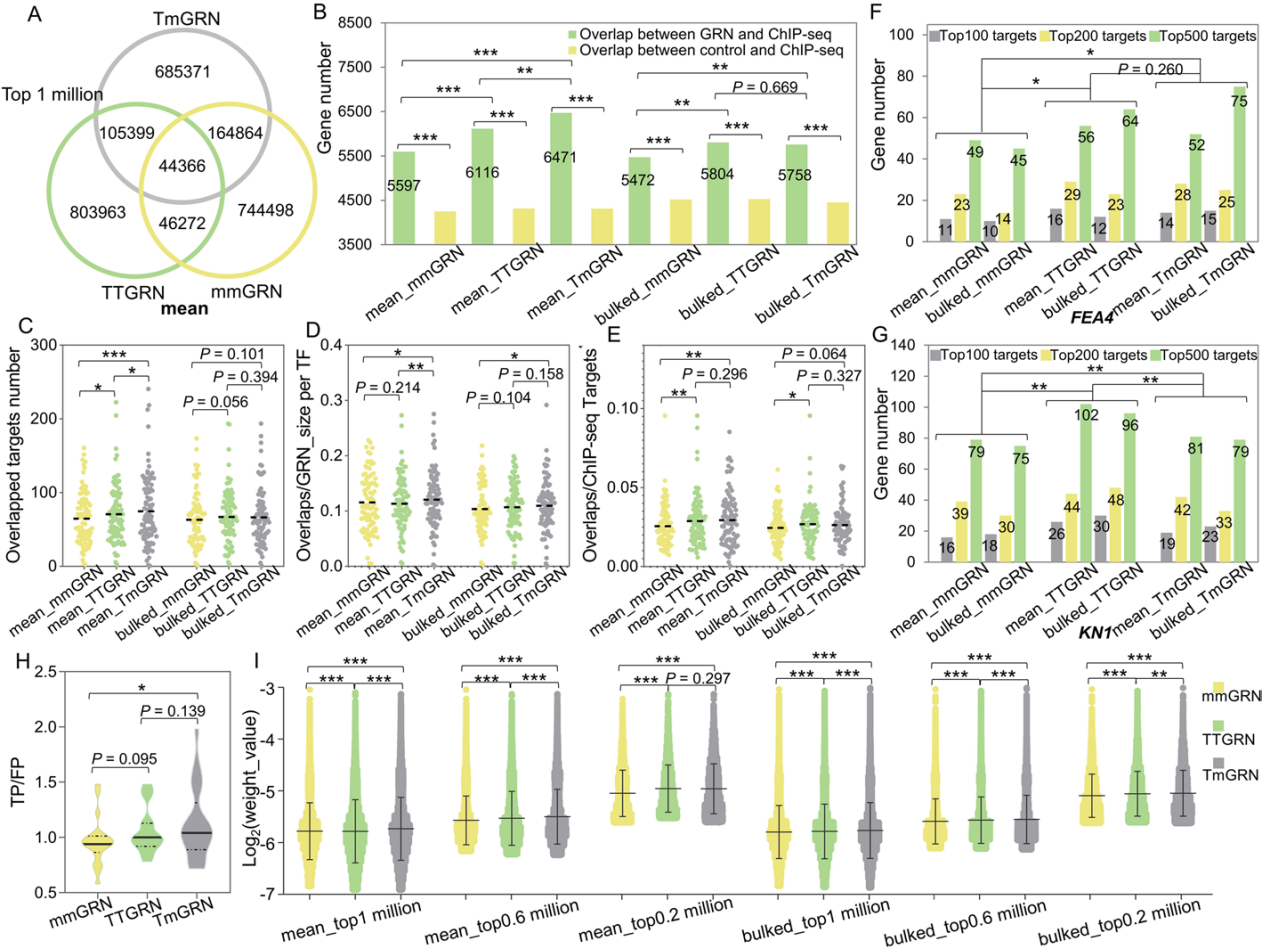
Comparisons of TmGRNs, TTGRNs and mmGRNs across different scenarios. **A** Overlap of mmGRNs, TmGRNs, and TTGRNs from mean data for the top 1 million edges. **B** Overlap between GRNs and ChIP-seq regulatory networks. The control consisting of TF-target pairs was generated randomly using the same TF sets and target sets for each dataset. The random TF-target pairs were generated 1,000 times for each GRN in each dataset. The average value of the overlaps between random targets and ChIP-seq targets was compared to the overlaps between GRN predictions and ChIP-seq targets using the *χ*^2^ test. In addition, because three types of GRN were constructed using the same TFs set and targets set in each dataset, the comparisons of overlapping targets among mmGRN, TTGRN, and TmGRN were also performed using the *χ*^2^ test. **C** The overlapped target number for each TF in 3 types of GRNs from both mean and bulked data (Student paired *t* test). **D** The overlaps between GRN and ChIP-seq for each TF after normalization by GRN size of each TF (Student paired *t* test). **E** The overlaps between GRN and ChIP-seq for each TF after normalization by ChIP-seq target number (Student paired *t* test). **F, G** Target comparisons between different GRNs and ChIP-seq for *FEA4* (**F**) and *KN1* (**G**). Top 100, top 200, and top 500 targets were considered. Significant differences were determined using the paired Student *t* test. **H** Simple Enrichment Analysis (SEA) [24] for the 1-kb sequences upstream for the start codons of the top 100 targets of 11 TFs in three types of GRNs, the center line in box represents the median. TP: Number of primary sequences matching the motif / number of primary sequences (percentage of primary sequences matching the motif); primary sequences are from the predicted targets. FP: Number of control sequences matching the motif / number of control sequences (percentage of control sequences matching the motif); control sequences are from the genes that randomly selected in the set of expressed genes. **I** Comparisons of GRN weights of three types of GRNs at different thresholds, significant differences were determined using the paired Student *t* test, center line in box represents the median. In all tests of significance above, “*” represents *P* < 0.05; “**” represents *P* < 0.01; “***” represents *P* < 0.001

To test the reliability of intra- and inter-omics GRNs, we analyzed the overlap between different types of GRNs and compared the results with those from a previous study [18]. TmGRNs and mmGRNs showed the highest overlap in all comparisons among the different types of GRNs (Fig. 3A, Additional file 1: Fig. S8). The number of overlapping edges detected in the three types of GRNs varied from 44,366 to 87,118 in the two datasets (Fig. 3A, Additional file 1: Fig. S8), each far more than the number of edges detected in GRNs built on the abundance of mRNAs and proteins in the previous study [18].

TFs usually bind to the promoter region of their target genes to regulate their transcription levels, thus defining the regulatome [25]. The translatome has been reported to show higher consistency with the proteome than with the transcriptome [20]. Therefore, we hypothesized that TmGRNs might better illustrate the regulatome than mmGRNs. To test this hypothesis, we conducted a comprehensive comparison between different GRNs and the regulatome built using ChIP-seq data from 86 TFs, target genes of these 86 TFs had been identified in both a previous study and this study [26]. We used three different thresholds (top 1 million, edge weight ≥ 0.0003 and edge weight ≥ 0.01) to test this hypothesis. At the threshold of “top 1 million,” we determined that our different GRNs show significantly more overlap with ChIP-seq data than the control GRNs (randomly generated network with the same gene sets of TFs and targets) (Fig. 3B). Importantly, TmGRNs and TTGRNs all displayed significantly more overlap than mmGRNs with ChIP-seq data across the three thresholds (Fig. 3B, Additional file 1: Fig. S9A and Fig. S10A). Compared to mmGRNs, TFs in TmGRNs and TTGRNs were more likely than those in mmGRNs to have more targets that were also detected in the ChIP-seq regulatome (Fig. 3C–E). However, TFs in TmGRNs have more targets also detected in the ChIP-seq regulatome than those in TTGRNs in some scenarios (Fig. 3C–E, Additional file 1: Fig. S9B-9G, Fig. S10B-10G, and Table S2). For example, four classical TFs (FASCIATED EAR4 [FEA4], KNOTTED1 [KN1], OPAQUE11 [O11], and ABSCISIC ACID INSENSITIVE 19 [ZmABI19]) showed more overlapping targets with ChIP-seq data in the TmGRNs than they did in the mmGRNs, and these TFs also showed more overlapping targets with ChIP-seq data in the TTGRNs than they did in the mmGRNs, except for O11 (Fig. 3F, G and Additional file 1: Fig. S11). We also noticed that TmGRNs have more overlap than TTGRNs with ChIP-seq data in multiple scenarios, although the significance was weak in a few cases (Fig. 3C–F, Additional file 1: Fig. S9-S11). Furthermore, we used area under the receiver operator characteristic curve (AUROC) and area under the precision-recall curve (AUPR) to evaluate the performance of each GRN (against ChIP-seq targets as benchmark) (Additional file 1: Fig. S12). The AUROC values ranged from 0.538 to 0.587, which were higher than the random predictions with a value of 0.500 and consistent to the previous study (Additional file 1: Fig. S12) [13]. Most values for AUROC and AUPR in TmGRNs and TTGRNs were higher than in the mmGRNs (Additional file 1: Fig. S12). The TmGRNs showed highest AUROC and AUPR values in the mean data source (Additional file 1: Fig. S12).

TFs generally bind to a specific region (motif) in the promoter of their target genes [25]. Although GRNs cannot distinguish between direct and indirect target genes, a certain proportion of direct targets should be detected. To test whether GRNs can capture the binding motifs of TFs, we extracted the top 100 target genes for 11 TFs (including the classical TFs O11 and Teosinte branched1 [Tb1], as well as TFs from the AUXIN-RESPONSE FACTOR [ARF], Homeodomain leucine zipper [HD-ZIP], ETHYLENE-RESPONSE FACTOR [ERF], MYB, basic leucine zipper [bZIP], basic helix-loop-helix [BHLH], and Golden2-like [G2-like] families) from the mmGRNs, TTGRNs, and TmGRNs. The 11 TFs are representative because they are from multiple different gene families and have well-known but divergent binding motifs (JASPAR, https://jaspar.genereg.net/). Satisfyingly, known motifs were identified in the 1-kb upstream of the start codons of these target genes using MEME [24]. Notably, we observed highest positive ratios in TmGRNs than in other GRNs (Fig. 3H, Table S3), suggesting that more real direct targets can be predicted by TmGRNs. In addition, we compared weights of overlapping GRN predictions (overlaps of the three types of GRNs) in mmGRNs, TTGRNs, and TmGRNs across different thresholds (top 0.2 million edges, 0.6 million edges, and 1 million edges) and observed that TmGRNs and TTGRNs have significantly higher weights than mmGRNs at each edge level (Fig. 3I). TmGRNs showed highest weights in most thresholds (Fig. 3I). These results indicate that translatome-related GRNs show better performance than mmGRNs that only based on transcriptome, and inter-omics GRNs likely outperform intra-omics GRNs for accurately representing the regulatome in maize.

### The multi-omics GRNs illustrate the potential functional landscape of maize

**Fig. 4:**
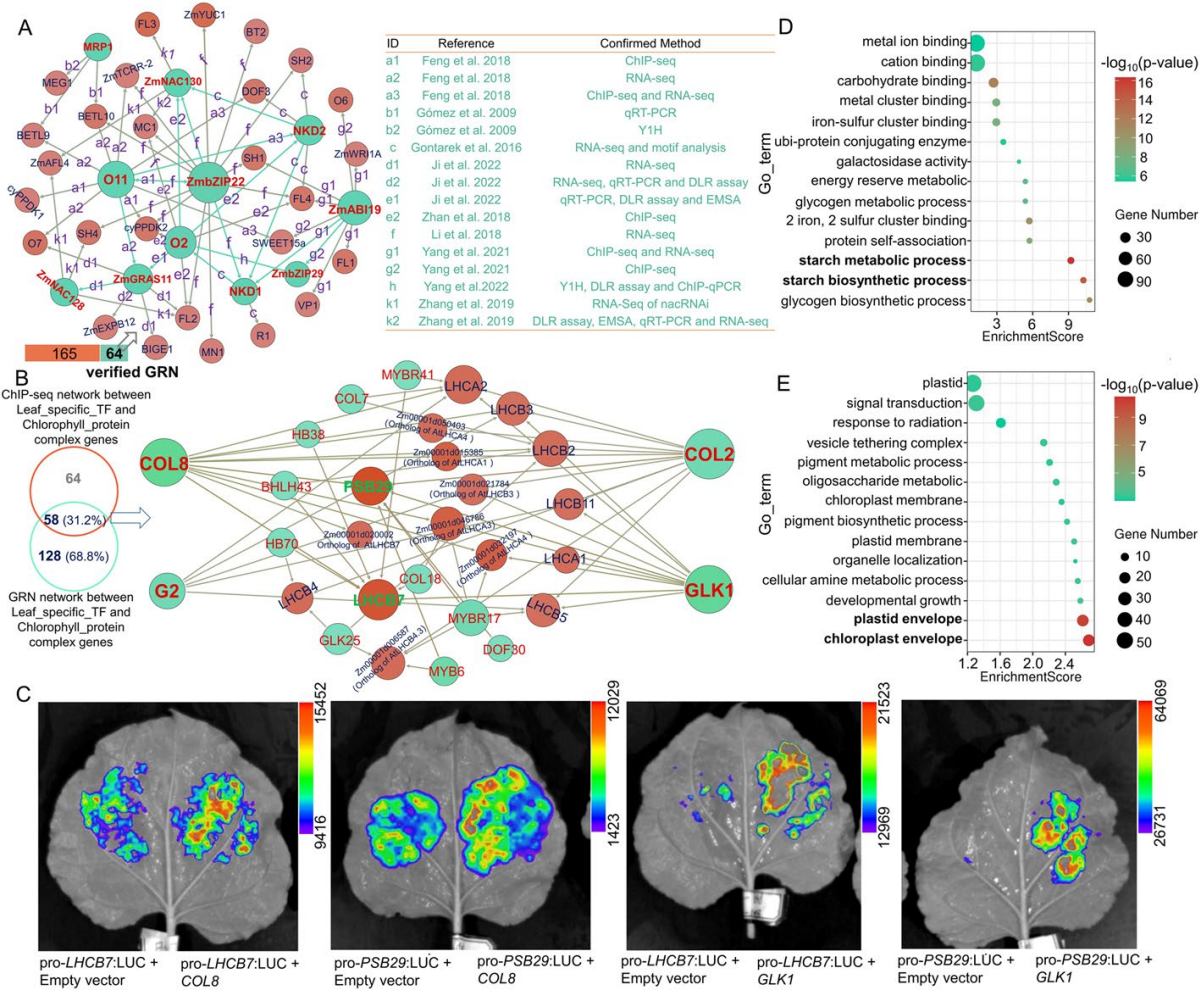
GRNs reconcile well-known regulatory networks. **A** Network of genes previously identified as related to kernel development re-built using GRNs. **B** GRN sub-network of leaf-specific TFs and chlorophyll a/b binding protein genes verified by ChIP-seq datasets. **C** Transient expression assay showing that the transcription output of *LHCB7* and *PSB29* promoters is upregulated by COL8 and GLK1 proteins. **D, E** GO enrichment for targets of kernel (**D**) and leaf related TFs (**E**)

To test whether the multi-omics GRNs convey biological meaning, we sought to validate our union GRN using some well-known genes and regulatory networks. Kernel size is an important agronomic trait with a complex regulatory mechanism [27, 28]. We extracted a GRN sub-network of 11 well-known kernel-related TFs from our union GRNs, the regulatory relationships of these 11 TFs had been systematically investigated in previous studies [29–37]. This kernel-related GRN sub-network contained 75 functional genes associated with kernel size that formed 229 gene-to-gene regulatory relationships [27, 29–90]. Of these 229 network edges, 64 (27.9%) were supported by previous studies based on different types of experiments, including ChIP-seq, RNA-seq, reverse transcription quantitative PCR (qRT-PCR), dual-luciferase reporter (DLR) assays, and yeast one-hybrid (Y1H) assays (Fig. 4A, Table S4), at a significantly higher (*χ*^2^ test, *P* value = 4.42×10^−7^) rate than by chance, suggesting the reliability of GRN predictions for biological inference.

Additionally, we used the translational abundance, as estimated from Ribo-seq data, to perform a hierarchical clustering analysis for 86 TFs with publicly available ChIP-seq data (Additional file 1: Fig. S13A) [26]; of which 29 were specifically translated in leaves (Additional file 1: Fig. S13A) [26] and might be associated with photosynthesis. A GRN sub-network of these 29 TFs revealed that they can target 23 LHC-like genes, which encode light-harvesting chlorophyll a/b binding proteins (Fig. 4B, Table S5). Notably, about 31.2% of the regulatory relationships for these TFs in GRNs could be also detected by ChIP-seq (Fig. 4B, Table S5), a number that is significantly higher (*χ*^2^ test, *P* value = 4.8×10^−4^) than random, suggesting the high accuracy of the GRNs. We noticed that TmGRN and TTGRN form more regulatory pairs for these TFs that could be confirmed by ChIP-seq compared to the mmGRN (Additional file 1: Fig. S14). Interestingly, four key TF genes including *CONSTANS 8* (*COL8)*, *G2, G2-like1 (GLK1)*, and *COL2* were shown to target 5-11 LHC-like genes detected by both GRNs and ChIP-seq, suggesting that their encoding proteins might have roles in photosynthesis (Fig. 4B). We selected two LHC-like genes (*LHCB7* and *PSB29*) regulated by *COL8* and *GLK1* and performed a transient luciferase reporter assay to verify their regulatory relationships. The results demonstrated that *COL8* and *GLK1* indeed appear to contribute to the transcription of *LHCB7* and *PSB29* (Fig. 4C)*.* Interestingly, we found that *COL8* and *GLK1* are likely to regulate the transcription of LHC-like genes independently (Additional file 1: Fig. S13B). Furthermore, we uncovered the 128 regulatory pairs that had not been previously identified by ChIP-seq (Fig. 4B and Fig. S15A). We selected two key TF genes (*BHLH145* and *HB38*) whose encoded proteins had more target genes, as well as two key target genes (Zm00001d020002, an ortholog of *AtLHCB7*; and Zm00001d024372, an ortholog of *AtLHCA1*) being targeted by more TFs for validation by luciferase reporter assays in maize protoplasts (Additional file 1: Fig. S15A). We determined that *BHLH145* could upregulate the transcription of Zm00001d020002 and Zm00001d024372, while *HB38* only induced that of Zm00001d020002 (Additional file 1: Fig. S15B and 15C), thus confirming the reliability of our constructed GRNs.

The characteristics of target genes reflect the potential biological functions of their upstream regulators. Here, we extracted the predicted targets of the 11 kernel-related TFs and the 29 TFs specifically expressed in leaves from union GRN, respectively. Accordingly, one thousand target genes were randomly selected from the two target sets to perform Gene Ontology (GO) enrichment. The target genes for TFs related to kernel development were significantly enriched in starch metabolic process (Fig. 4D). Similarly, the target genes of TFs specifically translated in leaves were significantly enriched in chloroplast envelope (Fig. 4E), suggesting that target prediction using GRNs is relatively accurate. Overall, the merged multi-omics GRNs constructed in our study appear to represent the potential functional landscape of maize with good accuracy. In addition, we identified specifically translated TFs and target genes as being simultaneously detected in specific tissues (Additional file 1: Fig. S16). Multiple sub-GRNs that more likely to exist in leaves, roots, SAM/embryo, and endosperm were extracted from the union GRN (Additional files 15, 16, 17 and 18, which were also deposited at http://zeasystemsbio.hzau.edu.cn/dataset.html). These specific regulatory networks may be helpful in investigating the functional landscape of these specific tissues in maize.

### Loss of ZmGRF6 function interrupts the expression of phytohormone-related genes and affects plant architecture

**Fig. 5:**
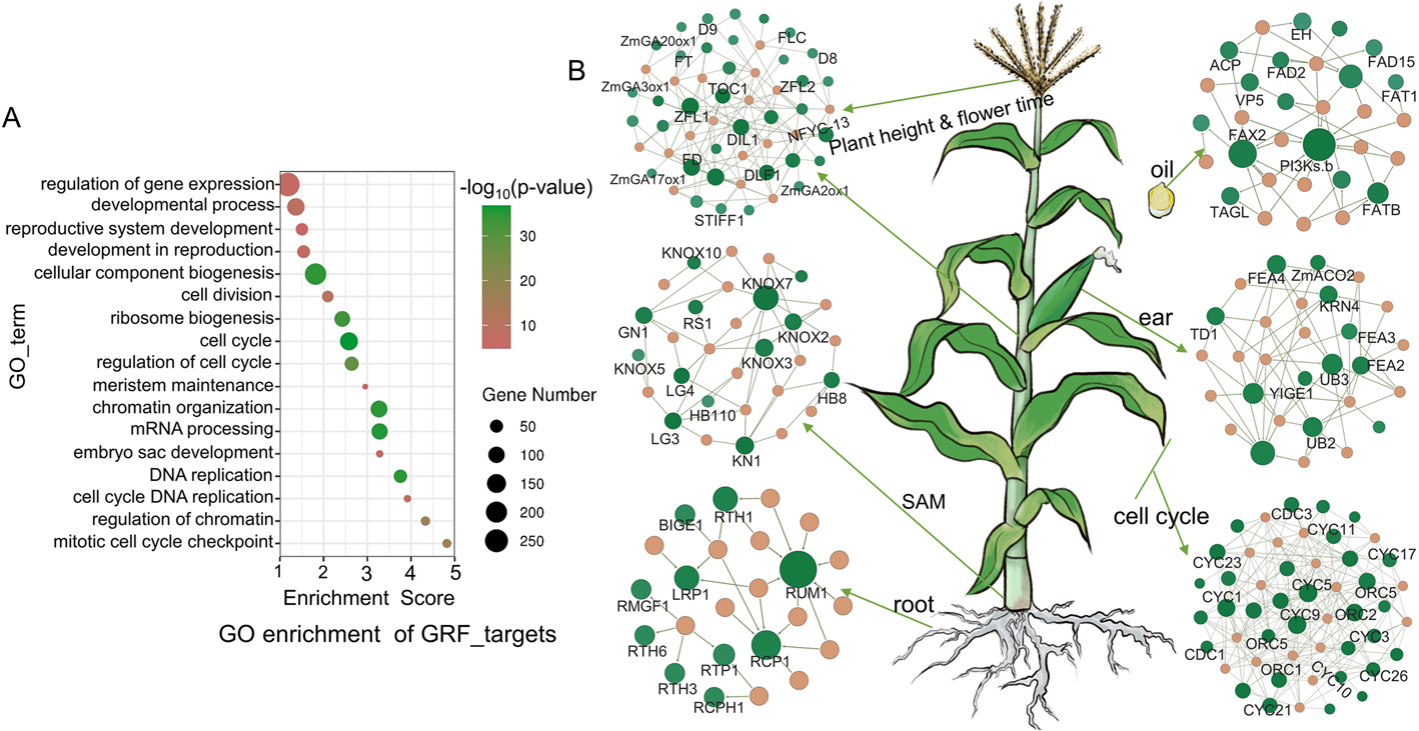
Regulatory landscape of the GRF family. **A** GO enrichment analysis for targets of the GRF family suggests that GRF TFs are associated with the cell cycle and development. **B** GRNs showing that members of the GRF family are likely related to the development of several organs in maize

The GROWTH-REGULATING FACTOR (GRF) family is a small, plant-specific family of TFs. However, understanding of their functions is limited, which promoted us to construct a GRF sub-network based on union GRN. GO enrichment of GRF targets in the GRN revealed that these targets are mainly associated with developmental processes and cell division (Fig. 5A). The regulatory landscape of the GRF family exhibited diverse growth-related functions in maize (Fig. 5B). GRF TFs target many genes with functional annotations associated with the development of roots, the SAM, ears, leaves, and plant height, as well as flowering and oil content (Fig. 5B). These GRF GRNs provide supporting evidence for us to hypothesize that GRFs function in the development of maize plant architecture.

**Fig. 6:**
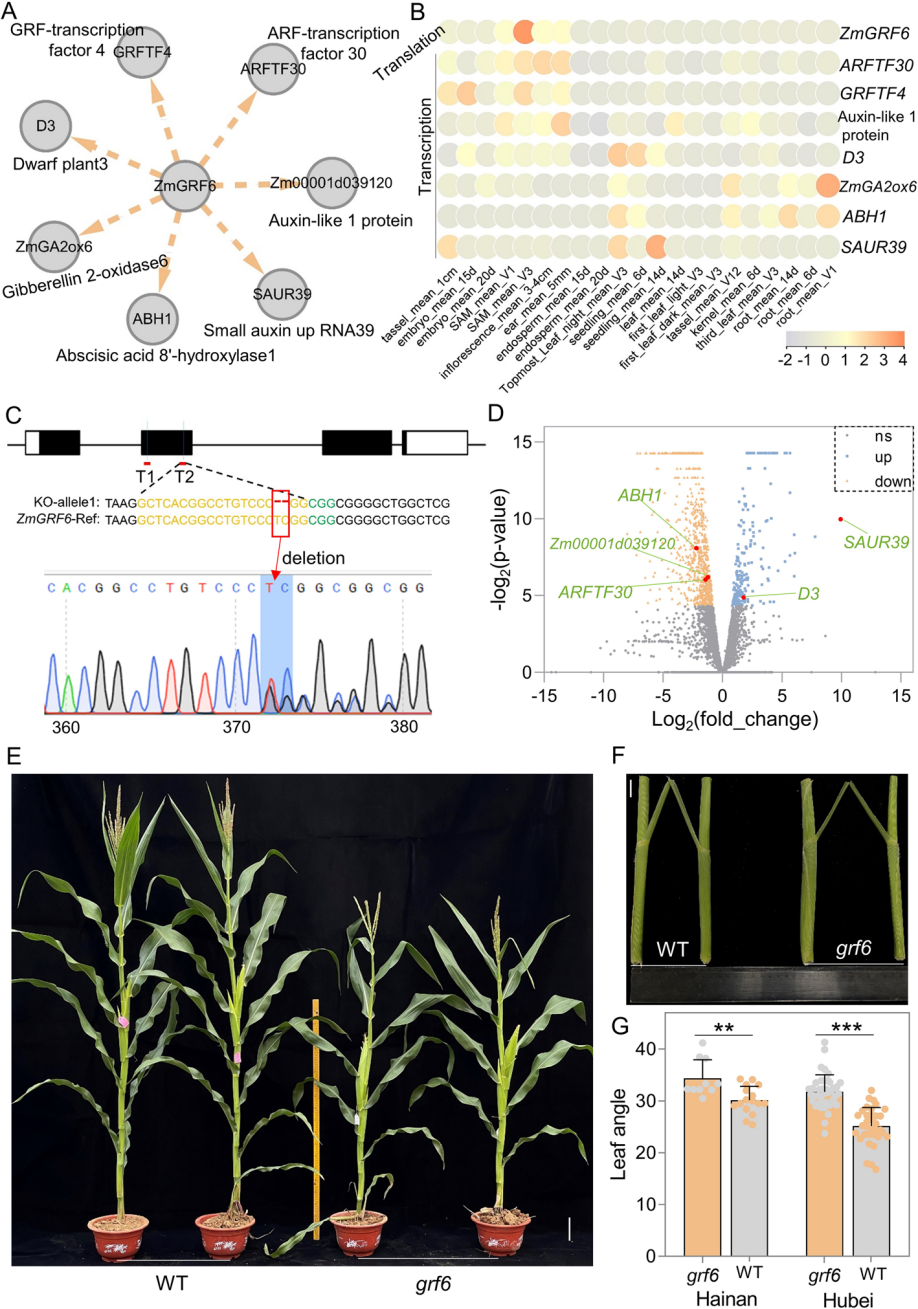
*ZmGRF6* likely regulates the expression of hormone-related genes and affects plant architecture of maize. **A** GRNs indicate that *ZmGRF6* targets some well-known phytohormone genes. **B** Translation pattern of *ZmGRF6* and transcription patterns of its potential targets across different tissues. **C** CRISPR knock-out mutant of *ZmGRF6*. **D** Differential expression analysis based on RNA-seq in the mutant and wildtype. **E, F** Phenotypes of WT (KN5585) and *grf6* mutants; scale bar: 10 cm (**E**) and 2 cm (**F**). **G** *grf6* mutant maize plants grown in Hainan (N_grf6_=10, N_WT_=16) and Hubei (N_grf6_=40, N_WT_=34), China, have significantly larger leaf angles than their WT (KN5585) counterparts. Significant differences were determined using the Student *t* test. “**” represents *P* < 0.01; “***” represents *P* < 0.001

To test the hypothesis inferred from the GRNs, we selected *GRF6* (*Zm00001d045533*), whose protein contains a conserved QLQ (glutamine, leucine, glutamine, IPR014978) domain and a WRC (tryptophan, arginine, cysteine, IPR014977) domain located within its N-terminus and targets multiple genes related to hormones such as auxin, gibberellin (GA), and abscisic acid (ABA) (Fig. 6A). Multiple targets predicted by GRN showed transcript patterns similar to the translation pattern of *ZmGRF6*, except the genes *Dwarf plant 3* (*D3*), *Gibberellin 2-oxidase6* (*ZmGA2ox6*), *Abscisic acid 8’-hydroxylase1* (*ABH1*), and *Small auxin up RNA39* (*SAUR39*), which might be related to the function of GENIE3 that can reveal non-linear relationships and both positive and negative linear relationships (Fig. 6B) [13]. We used the clustered regularly interspaced short palindromic repeat (CRISPR)/CRISPR-associated nuclease 9 (Cas9)-mediated genome editing to obtain a loss-of-function mutant of the *ZmGRF6* gene with a 2-bp deletion in the second exon, causing a frameshift mutation in the amino acid sequence (Fig. 6C). We performed RNA-seq on the wild type (KN5585) and mutant (*grf6*) and identified 1173 (*P* < 0.05) differentially expressed genes (DEGs) (Table S6). The overlap between DEGs and predicted GRF6 targets in union GRN was significantly higher than the random control (*χ*^2^ test, *P* = 0.041). Moreover, the overlap between DEGs and predicted GRF6 targets in TmGRNs and TTGRNs was more than that in mmGRNs (Additional file 1: Fig. S17). The transcript levels of *ARFTF30* and *Zm00001d039120* (encoding an auxin-like protein) were significantly downregulated in the *grf6* mutant (Fig. 6D, Additional file 1: Fig. S18B and 18C), suggestive of potentially decreased auxin contents in the mutant. Conversely, transcript abundance of *SAUR39* was promoted in the knock-out mutant (Fig. 6D, Additional file 1: Fig. S18B and 18C), suggesting that *SAUR39* may act as a negative regulator of auxin in maize. *D3*, a classic gene controlling GA biosynthesis, was upregulated in the mutant plants (Fig. 6D, Additional file 1: Fig. S18B and 18C), while *ZmGAox6*, controlling GA inactivation, was downregulated (Additional file 1: Fig. S18A and 18B), suggesting a potentially higher GA content in the mutant. In addition to genes related to auxin and GA, we also detected the downregulation of *ABH1*, a gene participating in ABA degradation (Fig. 6D, Additional file 1: Fig. S18B and 18C). Notably, we observed that the plant architecture of the *grf6* mutant was substantially different from that of its WT (KN5585) counterparts (Fig. 6E, Additional file 1: Fig. S19). Interestingly, the leaf angle in the *grf6* mutant was significantly larger than that in the WT (Fig. 6F, G). These results suggest that mutation of *ZmGRF6* likely perturbs phytohormone levels and affects the plant architecture of maize.

### ZmMYB31 may regulate the drought response through ABA and secondary metabolism pathways

MYB family members are among the most abundant and important TFs in plants [91], whose regulatory networks can be inferred from our GRNs. To dissect the functions of the MYB family, we constructed a GRN sub-network of MYB TFs to investigate their potential targets across the genome (Additional file 1: Fig. S20, Table S7). GO enrichment analysis of GRN targets (showing overlap in the three types of GRN, weight ≥ 0.05) for MYB TFs showed that they mainly participate in developmental processes, cell division, biosynthesis of microtubules and fibers, and abiotic stimulus (Additional file 1: Fig. S21).

**Fig. 7:**
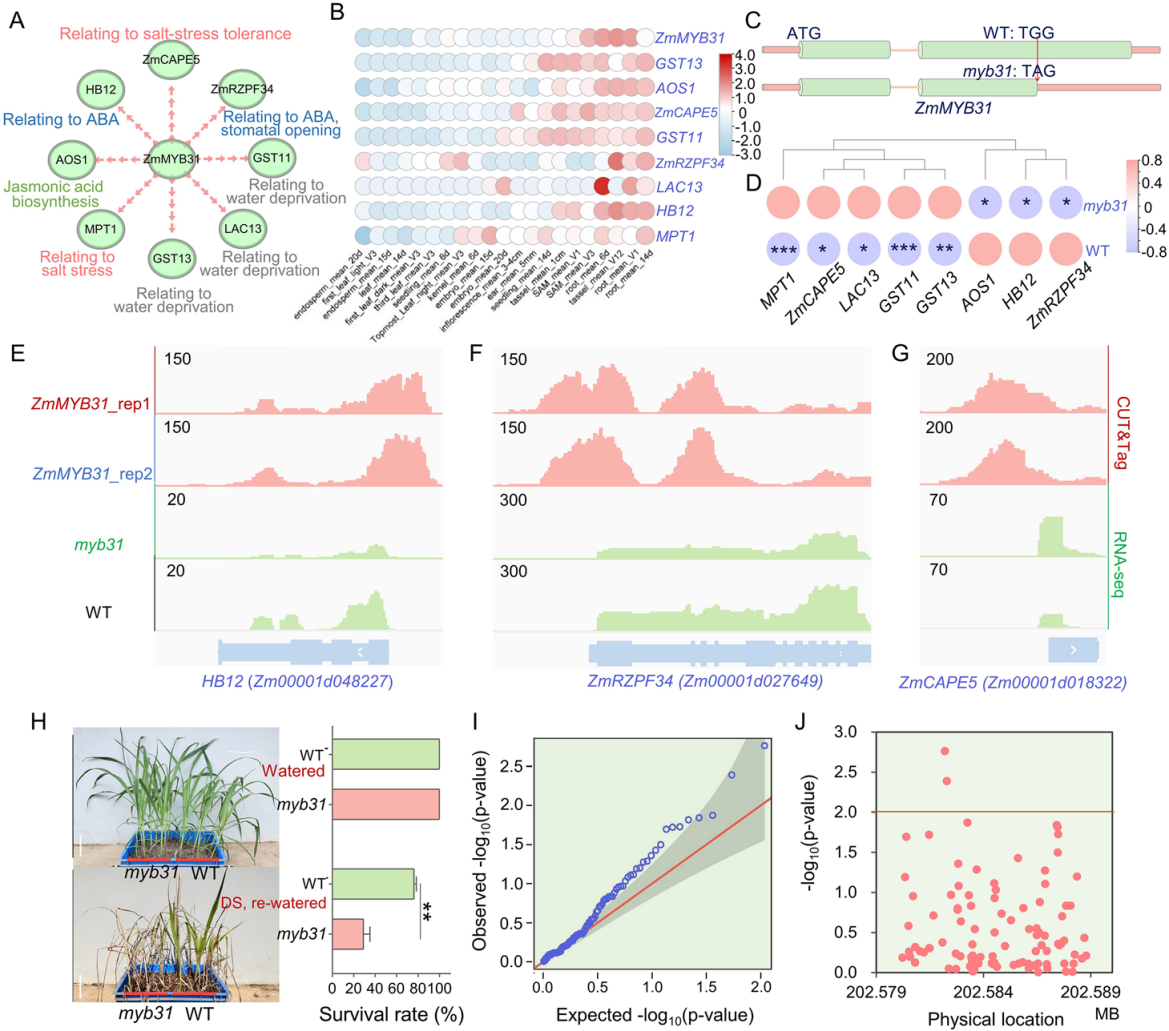
*ZmMYB31* regulates the expression of ABA-related genes, potentially affecting the maize drought response. **A** *ZmMYB31* targets multiple genes likely associated with the abiotic stress response. **B** *ZmMYB31* shows a similar expression pattern as the potential targets. **C** An EMS mutant of *ZmMYB31* with an early stop codon resulting in premature termination of translation. **D** Differential expression analysis of potential target genes in the mutant and WT determined by RNA-seq. **E–G** *ZmMYB31* targets *HB12* (**E**), *ZmRZPF12* (**F**), and *ZmCAPE5* (**G**). Both CUT&Tag and RNA-seq data support the regulatory relationships inferred from the GRNs. **H** The *myb31* mutant and WT exhibit significantly different drought responses, as determined by their survival rates (data are means ± SD; three replicates; paired Student’s *t* test; “**” represents *P* < 0.01); scale bar, 10 cm. **I, J** *ZmMYB31* is significantly associated with survival rate after drought treatment in maize inbred lines (association analysis in 347 maize lines using a general linear model [GLM])

Specifically, the GRN for *ZmMYB31* indicated that this TF regulates multiple genes related to jasmonic acid biosynthesis and the ABA pathway, suggesting a function in the responses to drought stress and salt stress (Fig. 7A). These target genes exhibited similar expression patterns to *ZmMYB31* (Fig. 7B). Fortunately, we obtained an ethyl methanesulfonate (EMS) mutant of *ZmMYB31* with an early stop codon in the coding region (Fig. 7C) [92]. RNA-seq of the WT and mutant (*myb31*) identified 3511 DEGs (*P* < 0.05) (Table S8), and the overlap between DEGs and GRN targets of *ZmMYB31* showed a significant difference compared with the random control (*χ*^2^ test, *P* = 0.046). The homologs of *Glutathione transferase11*(*GST11*), *GST13*, *Laccase13* (*LAC13*), and *mitochondrial phosphate transporter1* (*MPT1*) in Arabidopsis have been evidenced to be associated with oxidative stress, water deprivation, or salt stress [93]. Thus, we hypothesized that the *myb31* mutant is likely to be more sensitive to water deprivation or salt stress. Cleavage Under Target & Tagmentation (CUT&Tag), a new technology that reveals DNA-protein interactions, was used to identify the binding sites of MYB31 genome-wide. We established that the chromatin of the genes *HB12 (Homeobox-transcription factor 12)*, *ZmRZPF34 (Ring zinc-finger protein 34, Zm00001d027649)*, and *ZmCAPE5 (Zm00001d018322)* is directly bound by MYB31 (Fig. 7E–G and Additional file 1: Fig. S22; Table S9). Loss of MYB31 function increased the expression of *ZmCAPE5* (Fig. 7D), a gene homologous to *AtCAPE5* (*cysteine-rich secretory proteins, antigen 5*, and *pathogenesis-related 5*), which has been reported to negatively regulate salt stress tolerance in Arabidopsis [94]. Mutation in *ZmMYB31* inhibited the expression of *HB12* and *ZmRZPF34*, suggesting a direct positive regulation (Fig. 7D). In Arabidopsis, *ATHB5* (*HOMEOBOX PROTEIN 5*, homolog of the maize gene *HB12*) and *ATRZPF34* (homolog of *ZmRZPF34*) function in the ABA response [95]. Therefore, decreased expression of *HB12* and *ZmRZPF34* in the *myb31* mutant is likely to decrease the sensitivity of maize to ABA and inhibit stomatal closure during drought stress.

To validate the effect of *ZmMYB31* under drought stress conditions, we performed drought treatment and re-watering experiments. The *myb31* mutant was more sensitive to drought stress than its wild-type counterpart (Fig. 7H). Furthermore, we conducted association mapping to test for an association between genomic variation in *ZmMYB31* and the survival rate of seedlings of different inbred lines [96], which showed that multiple single-nucleotide polymorphisms (SNPs) distributed in the gene body of *ZmMYB31* are significantly associated with drought tolerance (Fig. 7I, J). These results support an important role for *ZmMYB31* in maize drought tolerance, confirming the hypothesis predicted by the GRNs.

**Fig. 8:**
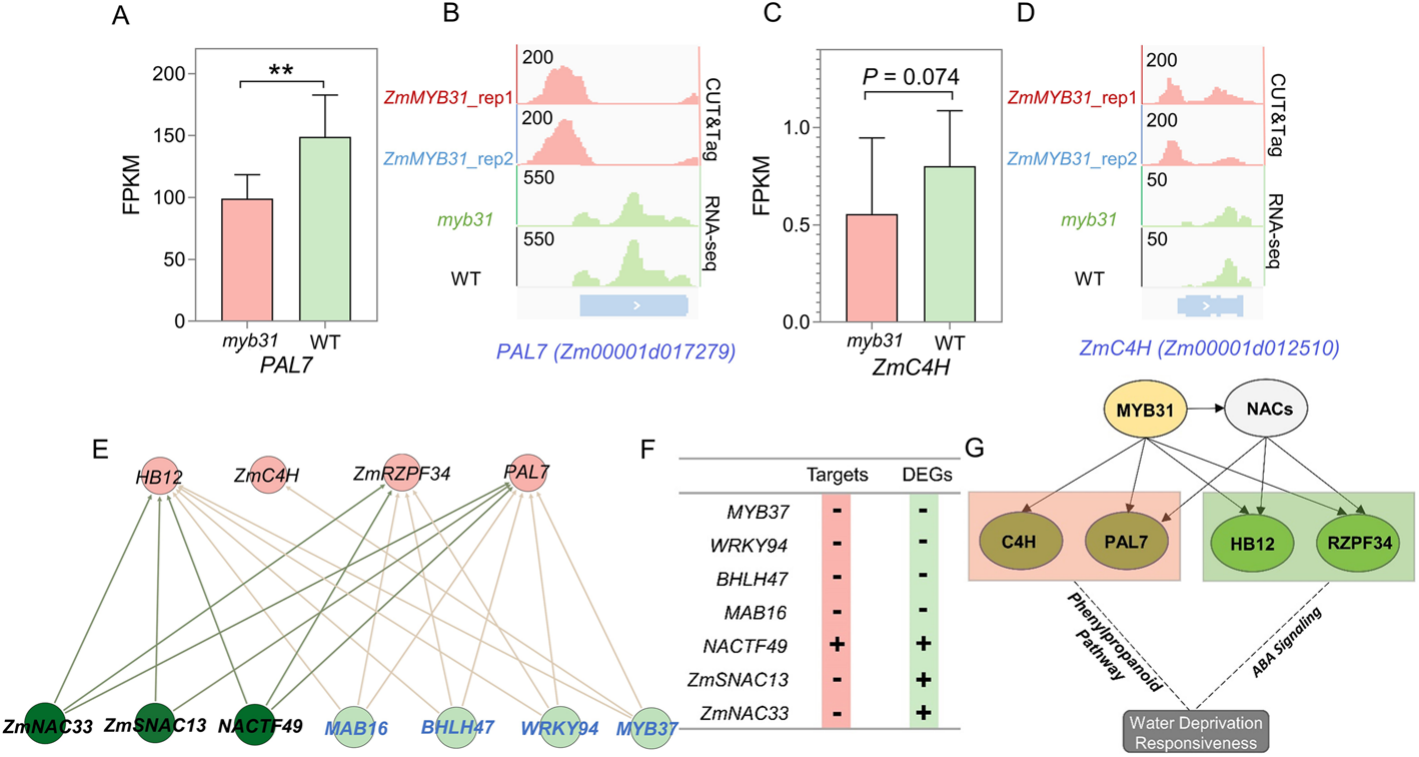
*ZmMYB31* modulates the phenylpropanoid pathway of maize drought response. **A, B** RNA-seq and CUT&Tag data showing that *Phenylalanine ammonia lyase7* (*PAL7*) is positively regulated by *ZmMYB31* (data are means ± SD; three replicates, “**” represents *P* < 0.01). **C, D** RNA-seq and CUT&Tag showing that *ZmC4H* (*Zm00001d012510*) is likely to be positively regulated by *ZmMYB31* (data are means ± SD; three replicates). **E** Putative regulatory network curated from public ChIP-seq data [26]. **F** Some TF genes in **E** are targeted by MYB31 and show differential expression between mutant and WT. **G** Proposed model of *ZmMYB31* regulated drought response through ABA signaling and the phenylpropanoid pathway

Flavonoids and lignin are two major metabolites of the phenylpropanoid pathway [97]. Increased accumulation of flavonoids can lead to enhanced tolerance against drought and oxidative stress in Arabidopsis [98, 99]. Transcriptome levels of *Phenylalanine ammonia lyase7* (*PAL7*) and *Cinnamate-4-Hydroxylase* (*ZmC4H*, Zm00001d012510), two key genes encoding enzymes from the upstream portion of the phenylpropanoid pathway, were likely downregulated in the *myb31* mutant (Fig. 8A, C). Importantly, ZmMYB31 directly bound to the promoter regions of *PAL7* and *ZmC4H*, suggesting that ZmMYB31 directly modulates *PAL7* and *ZmC4H* transcription (Fig. 8B, D). Therefore, we suspected that *ZmMYB31* would affect the drought tolerance of maize through effects on the phenylpropanoid pathway. By combining our GRNs with public ChIP-seq data for 104 TFs [26], we obtained a regulatory network for *PAL7*, *ZmC4H*, *HB12*, and *ZmRZPF34* (Fig. 8E). Three NAC (NAM, ATAF, and CUC) TF genes in the regulatory network showed differential expression between the *myb31* mutant and WT (Fig. 8F and Additional file 1: Fig. S23). Furthermore, the three TFs (*ZmNAC33, Stress-induced NAC 13* [*ZmSNAC13*] and *NACTF49*) all are known to positively regulate drought tolerance [100–102], and *NACTF49* also is targeted directly by MYB31. These results suggest that *ZmMYB31* likely regulates the drought tolerance of maize through ABA signaling and the phenylpropanoid pathway (Fig. 8G).

## Discussion

Although massive amounts of transcriptomic data have been generated in maize [12], an atlas at the translatomic level is still lacking, and inter-omics regulatory networks between the translatome and transcriptome have not been assembled in eukaryotes. In this study, we collected close-to-complete translatomic data spanning most tissues and growth stages of maize using Ribo-seq. Combining these datasets with transcriptomic data for the corresponding samples, we constructed a comprehensive transcriptome and translatome dataset of maize development. From this dataset, we identified 35,524 translated genes and 41,604 transcribed genes (FPKM >0) detectable in at least one tissue or sample.

TFs generally bind to specific DNA sequences in the genome to control chromatin status and transcription [25], which not only indicates a direct interaction between proteins and DNA, but also suggests a possible correlation between protein abundance of TFs and mRNA levels of their target genes. Several methods based on transcript abundance have been used to predict GRNs involved in plant growth and developmental processes [14–16]. However, the transcript abundance of TF genes is not always consistent with their protein abundance because the latter is buffered by multifaceted regulatory mechanisms in different biological processes [19], which may greatly reduce the accuracy of GRN prediction. Despite efforts to combine multiple omics datasets including mRNA, protein, and phosphoprotein [18], the low throughput of protein mass spectrometry (MS) restricts the breadth of GRNs. Here, we used a translatomic atlas, an important linker-by-proxy between mRNA and protein, to assess the protein levels of TFs and constructed inter-omics TmGRNs by combining these data with the transcript levels of target genes (Fig. 2B). We obtained 1,867 TFs and 26,738 potential target genes (maize V4 version genes, AGPv4) that were used to construct a genome-wide GRN containing 4,747,575 regulatory pairs (union GRN) and 130,500 high-confidence regulatory pairs, representing an almost complete multi-omics regulatome landscape of maize (Fig. 2C, D). We compared the union GRN to the existed GRN that constructed using the maize V3 version genes before [13, 18]. Most regulatory pairs (83.65%) in union GRN could be transformed into V3 version, and 8.79 and 36.48% of these pairs could be also detected by GRNs constructed by Huang et al. and Walley et al., respectively (Additional file 1: Fig. S24) [13, 18]. Although the overlapped rates are significantly higher than that of random control, they are relatively low. This might be affected by different tissue types, suggestive of the dynamic and complicated regulatome across maize.

TmGRNs and TTGRNs showed better performance compared to intra-omics mmGRNs based only on transcript abundance. In multiple scenarios, TmGRNs even showed better performance than TTGRNs. We predicted a greater number of regulatory pairs by the TmGRN compared to inter-omics pmGRNs (existed GRN constructed by Walley et al. previously) between protein levels and transcript levels [18], because there was more overlap between regulatory pairs identified in both mmGRNs and TmGRNs than between regulatory pairs identified in both mmGRNs and pmGRNs combining protein and mRNA levels. All these results demonstrate that translatome-related GRNs would be the better representative of regulatome.

GRNs are useful tools for investigating the relationships between TFs and other genes when RNA-seq (for mutant and WT) or ChIP-seq data are not available [12]. However, there remain some problems in the interpretation of their results. First, GRNs can present many false positives. In general, networks constructed using limited number of input samples tend to show lower performance and introduce more false positives. With a limited number of samples, networks tend to have lower weight values and reduced quality of prediction, particularly when more input genes are used during network construction. As an alleviation measure, the integration of multiple omics datasets greatly improves the predictive power of GRNs, and increasing sample size also has a positive effect on network performance [12, 18]. Therefore, we constructed mmGRNs (transcriptome level), TmGRNs (inter-omics between translatome and transcriptome), and TTGRNs (translatome level) based on different groups of data—the mean and bulked—to improve the accuracy of our predictions (Fig. 2A, B). Further overlapping different types of GRNs and improving the threshold of GRNs remains an effective way of increasing the rate of positive predictions. Incorporating other algorithms, such as weighted gene co-expression network analysis (WGCNA), is also helpful in reducing the number of false positives [103]. Another problem with GRNs is their inability to distinguish between direct and indirect targets. It is essential to verify direct regulatory relationships of high-confidence targets using molecular assays such as electrophoretic mobility shift assay (EMSA), DLR, and Y1H. An in silico tool called TDTHub (TF Binding Site-Discovery Tool Hub) was recently developed for the analysis of TF binding sites in plants, which may help distinguish between direct and indirect target genes [104].

GRNs can be utilized to help predict the function of TFs. For example, *MYB31* was reported to affect total lignin content and downregulate the expression of several genes related to monolignol biosynthesis when overexpressed in Arabidopsis, causing abnormal growth [105]. Further analysis suggested that overexpression of *MYB31* in Arabidopsis positively regulated the expression of C-repeat-binding-factor (CBF) genes and enhanced tolerance to chilling and oxidative stress [106]. In this study, we showed that maize MYB31 targets ABA-related genes (*HB12* and *ZmRZPF34*) and phenylpropanoid-related genes (*ZmC4H* and *PAL7*) through either direct or indirect means, suggesting a potentially important role of *ZmMYB31* in drought response. Multiple studies support the existence of ABA-signaling-mediated stomatal movement in response to drought stress [107, 108]. Flavonoids and lignin are metabolites produced from the phenylpropanoid pathway, both of which are associated with drought tolerance in plants [98, 99, 109]. Therefore, *ZmMYB31* may affect the drought response through two pathways. We noticed that *ZmMYB31* acts as a negative regulator to reduce major lignin-derived compounds but raised the contents of other lignin compounds, including ferulic acid and *p*-hydroxybenzaldehyde. Changes in expression of many metabolism-related genes during overexpression of *ZmMYB31* might therefore be responsible for changes in the drought response of maize plants [105]. In contrast to the negative regulation by MYB31 of the genes encoding 4-coumarate 3-hydroxylase (C3H), 4-coumarate-CoAligase (4CL), ferulate-5-hydroxylase (F5H), caffeic acid O-methyltransferase (COMT), caffeoyl shikimate esterase (CSE), and cinnamoyl-CoA reductase 3 (CCR3) [105, 110, 111], we found that *ZmC4H* and *PAL7* expression are positively regulated by MYB31. This difference is probably associated with the different functions of the encoded enzymes upstream of the phenylpropanoid pathway. Additionally, we utilized our multi-omics GRNs to predict and validate the function of the TF *GRF6*. Together, these results suggest that multi-omics GRNs can reveal the functional landscape of maize.

## Conclusions

In summary, we compiled an almost complete set of translatomic and transcriptomic datasets for the maize reference inbred line B73 and established a high-quality multi-omics GRN covering most tissues and developmental stages throughout the life cycle of the plant. Inter-omics GRNs constructed by combining translatome and transcriptome data showed better performance than those only based on single omics data in most cases. We identified the growth regulator *ZmGRF6* and broadened our understanding of the function of *ZmMYB31* in the drought response using the GRN. The molecular mechanisms of the two TFs interpreted in this study will be helpful for improvement of agronomic traits, especially for plant architecture and drought tolerance.

## Methods

### Plant materials

Twenty-one tissues were profiled previously as two biological replicates; detailed information for these samples is summarized in supplemental Table S1. In addition, twenty bulk samples were collected from maize plants grown in the greenhouse under a 12-h light/12-h dark photoperiod at 25 °C (Table S1). Three replicates were collected per tissue, and each replicate was from three individuals. Equal amounts of the three replicates were combined to make the bulk sample that was analyzed by Ribo-seq and RNA-seq. The translational levels of genes in the identical tissues between two times of sampling collection were used to measure the correlation coefficient (Pearson) to assess the data reliability.

### RNA extraction and sequencing

Total RNA was isolated from all tissues using a Direct-zol RNA Microprep kit (Zymo Research) following the manufacturer’s instructions. Reverse transcription was performed to synthesize first-strand cDNAs as templates for preparing mRNA-seq libraries. All RNA-seq libraries were constructed using a Ribo-Zero rRNA Removal kit followed by a TruSeq Stranded Total RNA Library Prep Plant (Illumina) and then sequenced on an Illumina NovaSeq platform with PE150.

### Ribosome profiling (Ribo-seq)

Tissue samples of at least 5 g each were pulverized and dissolved in extraction buffer (44 mM Tris-HCl, pH 7.5, 175 mM KCl, 13 mM MgCl_2_, 100 mg/mL cycloheximide, 15 mM 2-mercaptoethanol, 1% [v/v] Triton X-100, 10 units/mL DNase I). The supernatant was treated with RNase I (10 units/μg RNA) at room temperature for 1 h and then the reaction was terminated by adding RNase inhibitor (20 units/μL). The solution was immediately transferred into a MicroSpinS-400 column to enrich for RNA-ribosome complexes (monosomes). Ribosome-protected fragments were extracted from samples using a miRNeasy RNA isolation kit (Qiagen) according to the manufacturer’s instructions. After removing rRNA, the remaining RNAs were used to construct libraries using a strategy for small RNAs, which were sequenced on an Illumina HiSeq X Ten platform.

### Data analysis

For RNA-seq, clean data from all samples were mapped to the B73 AGPv4 reference genome using HISAT2 (hisat2/2.1.0) with default parameters [112]. Unique reads were used to measure expression levels of genes using StringTie (StringTie/1.3.0-foss-2016b) [113] with the parameter “-G -e -A”.

For Ribo-seq, quality control of reads was performed using FASTX_Toolkit-0.0.14 (http://hannonlab.cshl.edu/fastx_toolkit/index.html), and clean reads were then aligned to the rRNA reference sequences using Bowtie-1.1.2 [114] to remove rRNA sequences. The remaining reads were mapped to the maize AGPv4 reference genome using HISAT2 (hisat2/2.1.0) [112], and the expression levels of genes were measured using StringTie (StringTie/1.3.0-foss-2016b) [113] using the unique reads.

### Establishment of GRNs

The abundance of transcripts and ribosome-protected products of corresponding genes in the bulked samples were used to construct GRNs. In parallel, the mean expression values from rep1 and rep2 were also be used to construct GRNs. Genes with FPKM ≥ 1 in at least one tissue and non-zero values in at least three tissues were used to construct GRNs. These GRNs were constructed on the basis of the expression matrix using the classic software GENIE3 [115] with the random forest algorithm. Regulatory pairs under the top 1 million edges in GRNs were considered.

### Transient luciferase reporter assay

Transient expression assays were performed in *Nicotiana benthamiana* leaves, as previously described [116]. The promoters of *LHCB7* and *PSB29* were individually cloned upstream of the firefly luciferase (*LUC*) reporter gene via the *Kpn*I and *Pst*I sites of the pGreenII 0800-LUC vector. The full-length coding regions of *COL8* and *GLK1* were cloned into the pGreenII 62-SK vector downstream of the 35S promoter between the *EcoR*I and *Xho*I restriction sites as effector constructs. The two types of plasmids were then individually transformed into Agrobacterium (*Agrobacterium tumefaciens*) strain GV3101. Equal volumes of cells of different combinations of strains were mixed and co-infiltrated into *N. benthamiana* leaves. After 48–72 h, co-infiltrated leaves were collected and sprayed with 100 mM luciferin. After leaves were placed in darkness for 5 min, image acquisition was performed using a low-light, cooled, charge-coupled device imaging apparatus.

Transient expression assays were also performed in maize leaf protoplast. Protoplast isolation and transfection were performed as previously reported [26]. The promoters of *Zm00001d020002* and *Zm00001d024372* were individually cloned into pGreenII 0800-LUC vector using the same method as above*.* The full-length *BHLH145* and *HB38* coding sequence were also cloned into pGreenII 62-SK vector using the same method as above*.* The same amount of plasmid DNA from the appropriate pairs of constructs was mixed and transfected into protoplasts. After 14h (dark condition), the protoplasts were collected and processed using a Dual-Luciferase Reporter Gene Assay Kit (YEASEN) according to the manufacturer’s instructions. The LUC and REN activity levels in the resultant were detected in a microplate reader.

### CRISPR/Cas9-mediated gene editing

Two guide RNAs (gRNAs) targeting the second exon of *ZmGRF6* were designed using CRISPR-P [117]. The vector carrying the two gRNAs was introduced into immature maize “KN5585” embryos using Agrobacterium-mediated transformation by WIMI Biotechnology Co., Ltd. Heterozygous T_1_ plants were self-pollinated, and homozygous mutants were obtained from the segregating population (T_2_).

### CUT&Tag and bioinformatics analyses

The full-length coding sequence of *ZmMYB31* was cloned into the pM999 vector downstream of the cauliflower mosaic virus (CaMV) 35S promoter and upstream of the GFP sequence via the *Xba*I restriction site. Protoplast isolation and transfection with the resulting plasmid were performed as previously described [26]. Protoplast cells with successful expression (based on GFP fluorescence) of the plasmid were lysed, and the following steps were performed using a Hyperactive Universal CUT&Tag Assay Kit (Vazyme) based on the manufacturer’s guidelines. Successfully constructed libraries were sequenced on an Illumina NovaSeq platform. Clean reads were mapped to the B73 reference genome (AGPv4) using Bowtie2 software [118]. A search for high-confidence peaks (peaks *P* < 1×10^−5^) was performed using MACS with the parameters “callpeak -g 2.2e+9 -s 150 -B -p 1e-5 -f BAMPE” [119]. Distribution of peaks over the entire genome was analyzed using ChIPseeker [120]. If a peak was located within the range of 3 kb upstream to 3 kb downstream of a gene, then this gene was assumed to be the target of the protein (ZmMYB31-GFP in this case).

### RNA-seq for mutants and differential expression analysis

Leaf samples were collected as two replicates from the *grf6* mutant and the WT (V10 stage), while root samples were collected as three replicates from the *myb31* mutant and the WT (V3 stage) for sequencing. Extraction of total RNA and library construction were performed as described above. Clean data were mapped to the B73 AGPv4 reference genome using STAR software (version 2.7.3a) with default parameters [121]. Unique reads were used for analysis of differential expression using the Cufflinks (version 2.2.1) with package Cuffdiff [122].

### Real-time quantitative reverse transcription PCR (qRT-PCR)

RNA (500 ng) was used for synthesis of the first-strand complementary DNAs (cDNAs) in 10 μl of reaction mixture using the HiScript II Q RT SuperMix for qPCR (+gDNA wiper) (Vazyme, Nanjing, China). qRT-PCR was performed using the Taq Pro Universal SYBR qPCR Master Mix (Vazyme, Nanjing, China) on the CFX96 Real-time system (BIO-RAD, Hercules, the USA). The maize actin gene was used as internal reference. Three replicates of qRT-PCR were performed for the validation of RNA-seq data. The primer sequences are listed in Table S10.

### Drought treatment of the myb31 mutant and WT

Seeds of the mutant and WT were planted into soil (peat: perlite [70:30]) and grown to the V3 stage (growth room kept at 22–26 °C, with a 16-h light/8-h dark photoperiod). Watering was withheld from all seedlings until plants showed wilting (approximately 13–16 days). Watering was then resumed for 5 days before phenotypic changes were recorded.

### Motif enrichment analysis (SEA), GO enrichment, and association analysis

According to the ranked weights, the 1-kb sequences upstream of the start codon were extracted for the top 100 target genes. Motif enrichment analysis in these sequences was performed using the web software MEME with the function of Simple Enrichment Analysis (SEA) (https://meme-suite.org/meme/tools/sea). GO enrichment analysis was conducted using agriGO (http://systemsbiology.cau.edu.cn/agriGOv2/) [123]. A false discovery rate (FDR ≤ 0.05) was used to identify significant GO terms.

A natural population with 347 maize inbred lines was used to perform association analysis. The survival rate of seedlings from these maize lines under drought stress was investigated previously and used here [96]. The gene body of *ZmMYB31* contained 107 high-confidence SNPs. Association analysis was performed using the software GAPIT3 [124] with general linear model (GLM).

## Supplementary Information

**Additional file 1.** Supplementary figures S1-S24.

**Additional file 2: Table S1.** All samples used in this study.

**Additional file 3: Table S2.** Overlap between GRNs and CHIP-seq for single TF.

**Additional file 4: Table S3.** Motif enrichment for top 100 targets of 11 TFs.

**Additional file 5: Table S4.** Kernel related network predicted by GRN.

**Additional file 6: Table S5.** GRN network can be supported by CHIP-seq.

**Additional file 7: Table S6.** DEGs between grf6 mutant and its WT.

**Additional file 8: Table S7.** A sub-GRN for MYB related TFs.

**Additional file 9: Table S8.** DEGs between myb31 mutant and its WT.

**Additional file 10: Table S9.** Target genes of MYB31 identified by CUT&Tag.

**Additional file 11: Table S10.** Primer information used in the qRT-PCR.

**Additional file 12: Table S11.** Accessions for the data sets used in this study.

**Additional file 13.** Merged regulatory pairs predicted by Union GRN.

**Additional file 14.** Predicted regulatory pairs of high-confidence GRN.

**Additional file 15.** Predicted sub-GRN likely occurred in endosperm.

**Additional file 16.** Predicted sub-GRN likely occurred in leaf.

**Additional file 17.** Predicted sub-GRN likely occurred in root.

**Additional file 18.** Predicted sub-GRN likely occurred in SAM-embryo.

**Additional file 19.** Review history.

## Data Availability

The Ribo-seq and RNA-seq datasets of bulked samples are available at the Genome Sequence Archive (GSA) at the Big Data Center (National Genomics Data Center, NGDC) [125], the detailed accession number for each sample is in sheet1 of Table S11. The published Ribo-seq and RNA-seq datasets of samples with two replicates are available at the Genome Sequence Archive (GSA) at the Big Data Center (NGDC) and Sequence Read Archive (SRA) at National Center for Biotechnology Information (NCBI), the detailed accession numbers for each sample are in sheet2 and sheet3 of Table S11 [20, 23]. No custom scripts and software were used other than those mentioned in the “Methods” section.
