# Supplementary material for: A translatome-transcriptome multi-omics gene regulatory network reveals the complicated functional landscape of maize
Source: Genome Biol. 2023 Mar 29;24:60. doi: 10.1186/s13059-023-02890-4 (PMC10053466; doi:10.1186/s13059-023-02890-4)
Supplement: Supplementary file 1 — Additional file 1. Supplementary figures S1-S24. [file 13059_2023_2890_MOESM1_ESM.docx]

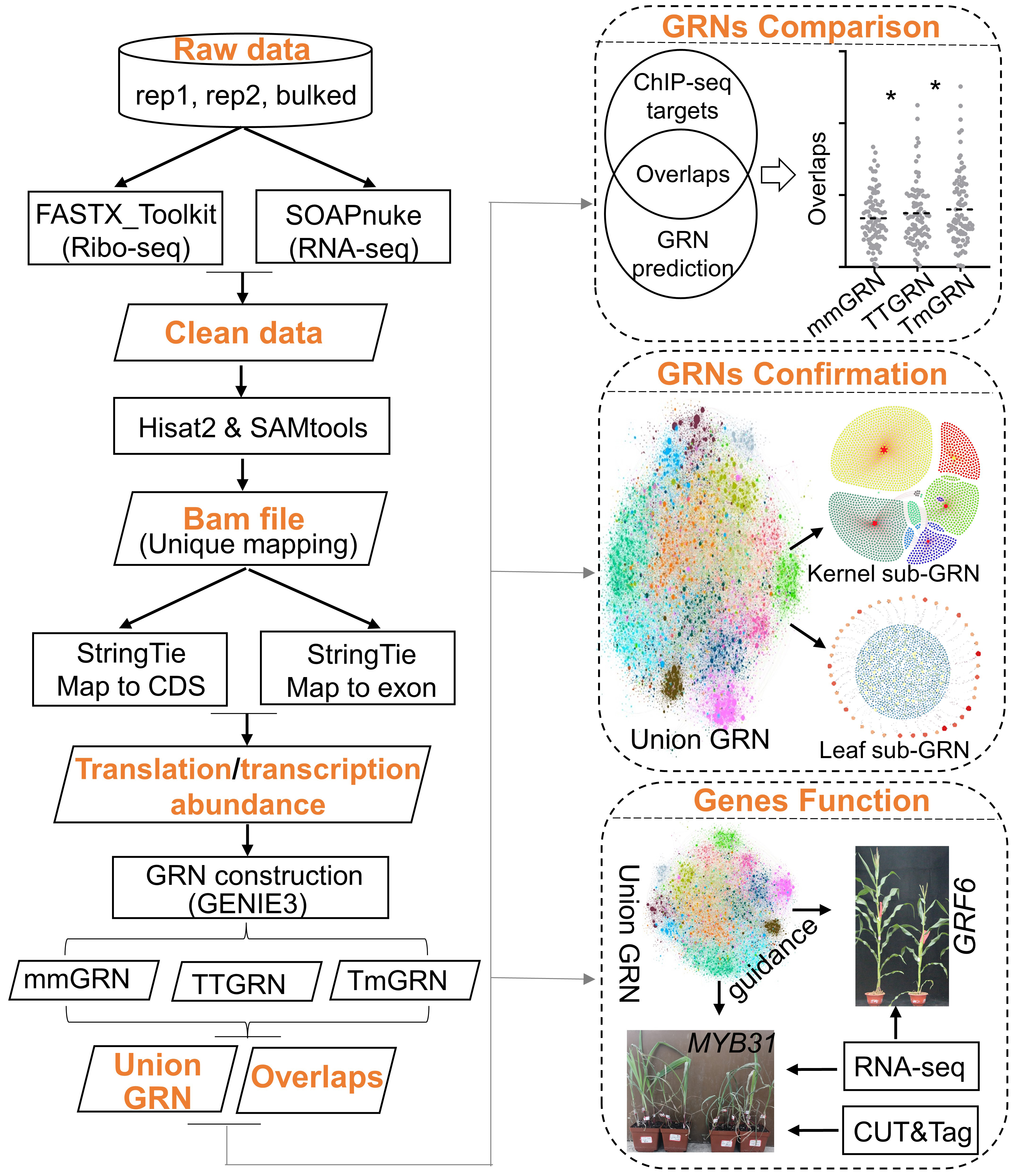


**Fig. S1 A flow chart of the study.**





**Fig. S2 High correlation between two replicates and bulked samples in both transcriptome and translatome.**

A. The identical tissues between two replicates and bulked replicate samples showed high correlation in transcriptome. B. The identical tissues between two replicates and bulked replicate samples showed high correlation in translatome.


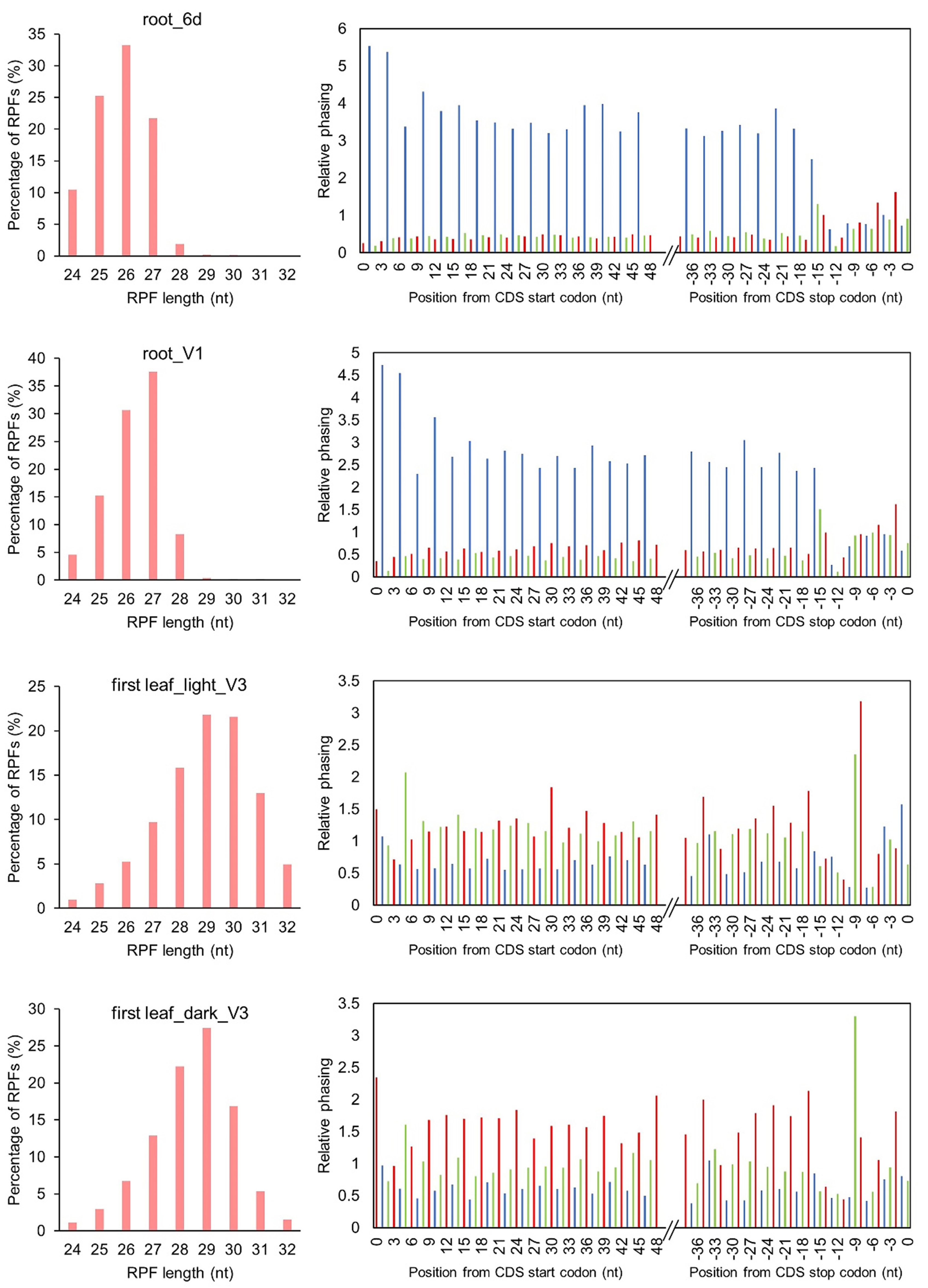


**Fig. S3** **Length distribution of ribosome-imprinted fragments (RPFs) and three-nucleotide periodicity within the first 50 nt and last 40 nt of CDSs for samples of root_6d, root_V1, first leaf_light_V3, first leaf_dark_V3.**


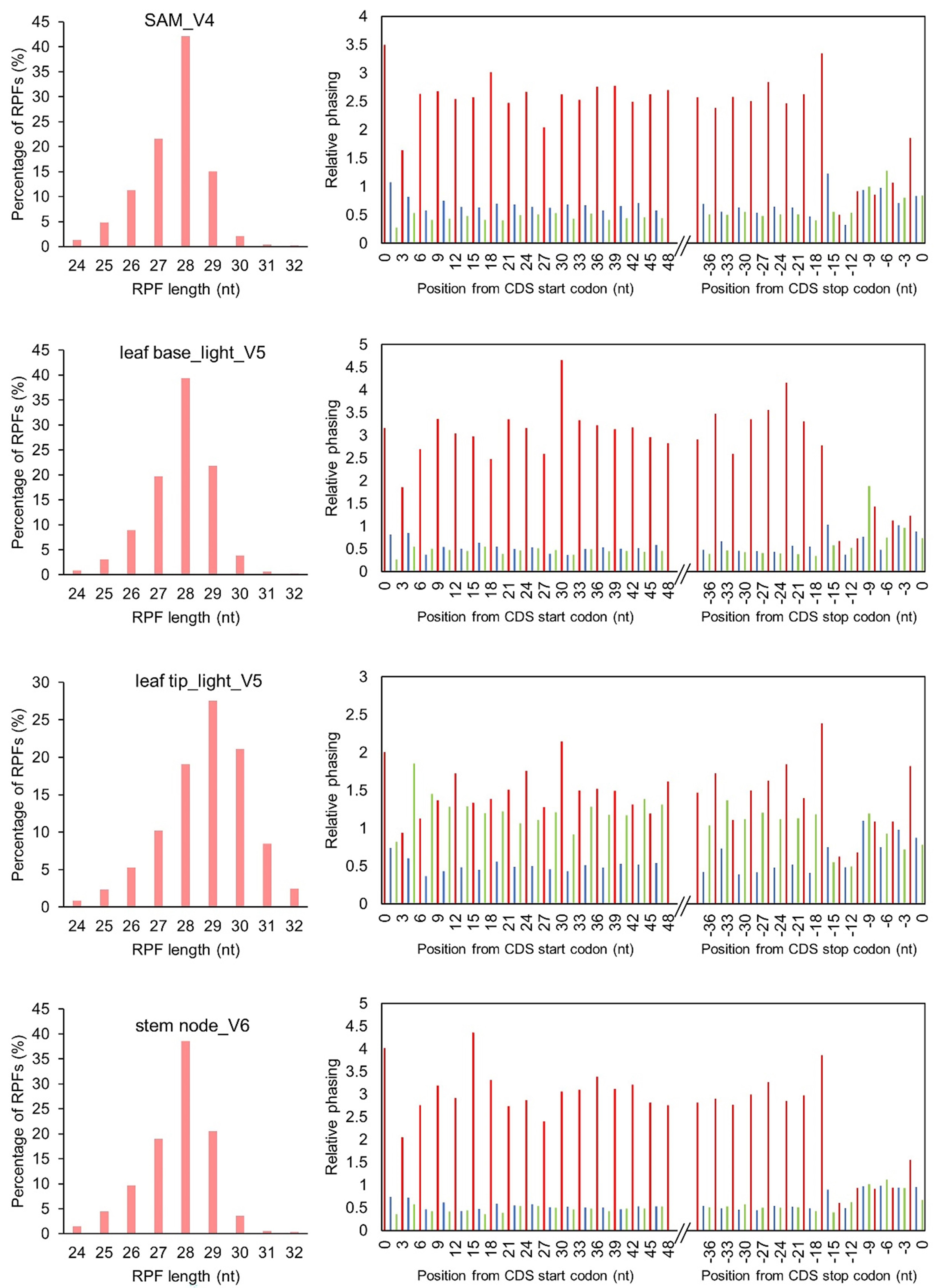


**Fig. S4** **Length distribution of ribosome-imprinted fragments (RPFs) and three-nucleotide periodicity within the first 50 nt and last 40 nt of CDSs for samples of SAM_V4, leaf base_light_V5, leaf tip_light_V5 and stem node_V6.**


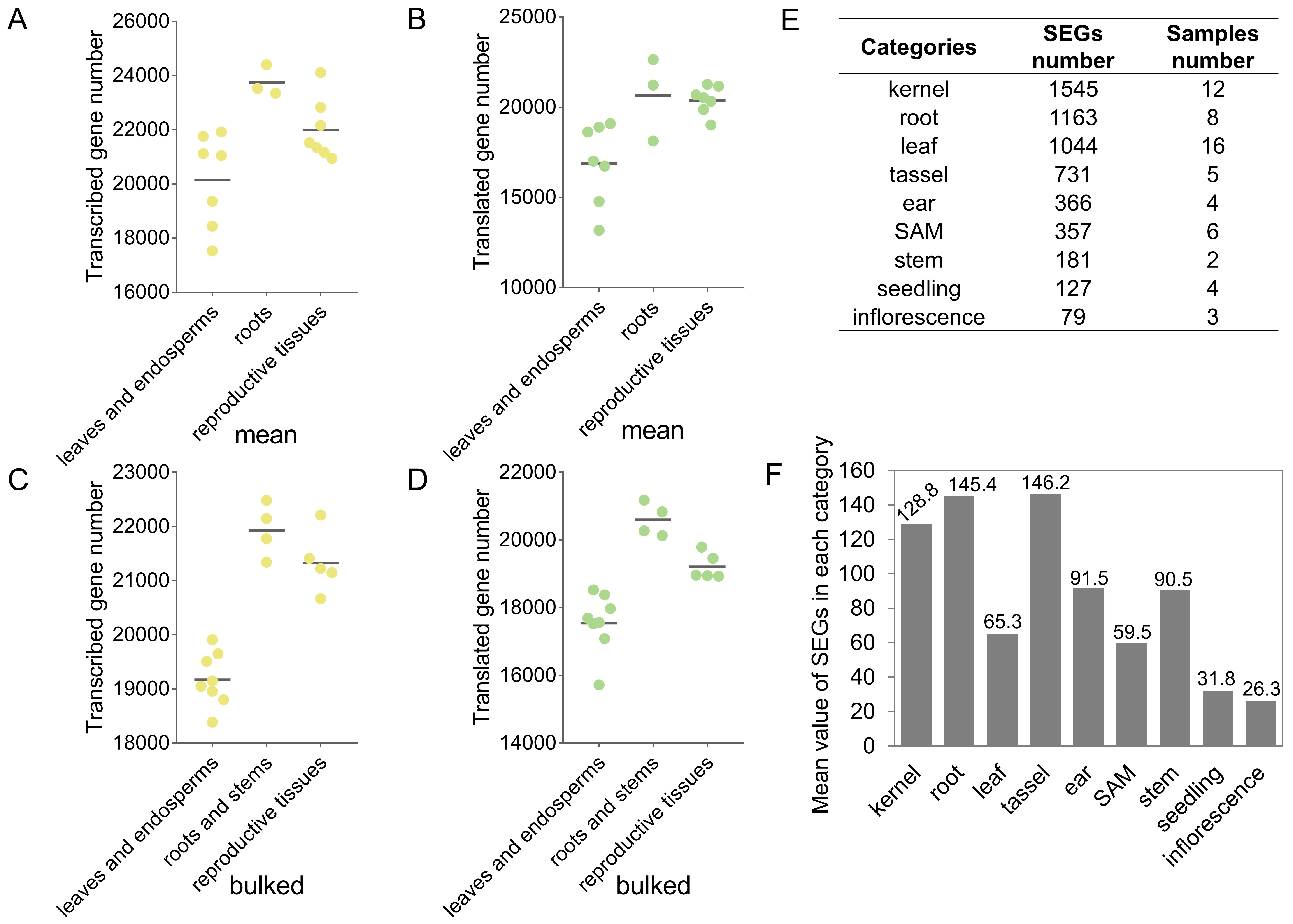


**Fig. S5** **Detected genes and SEGs in different tissue types.**

A-D, the detected gene number in leaves_endosperms (vegetative tissues), roots (vegetative tissues) and reproductive tissues including ear, tassel, inflorescence, embryo and kernel. E, SEG number and sample number in different categories. F, Mean SEGs in each category after divided by the sample number


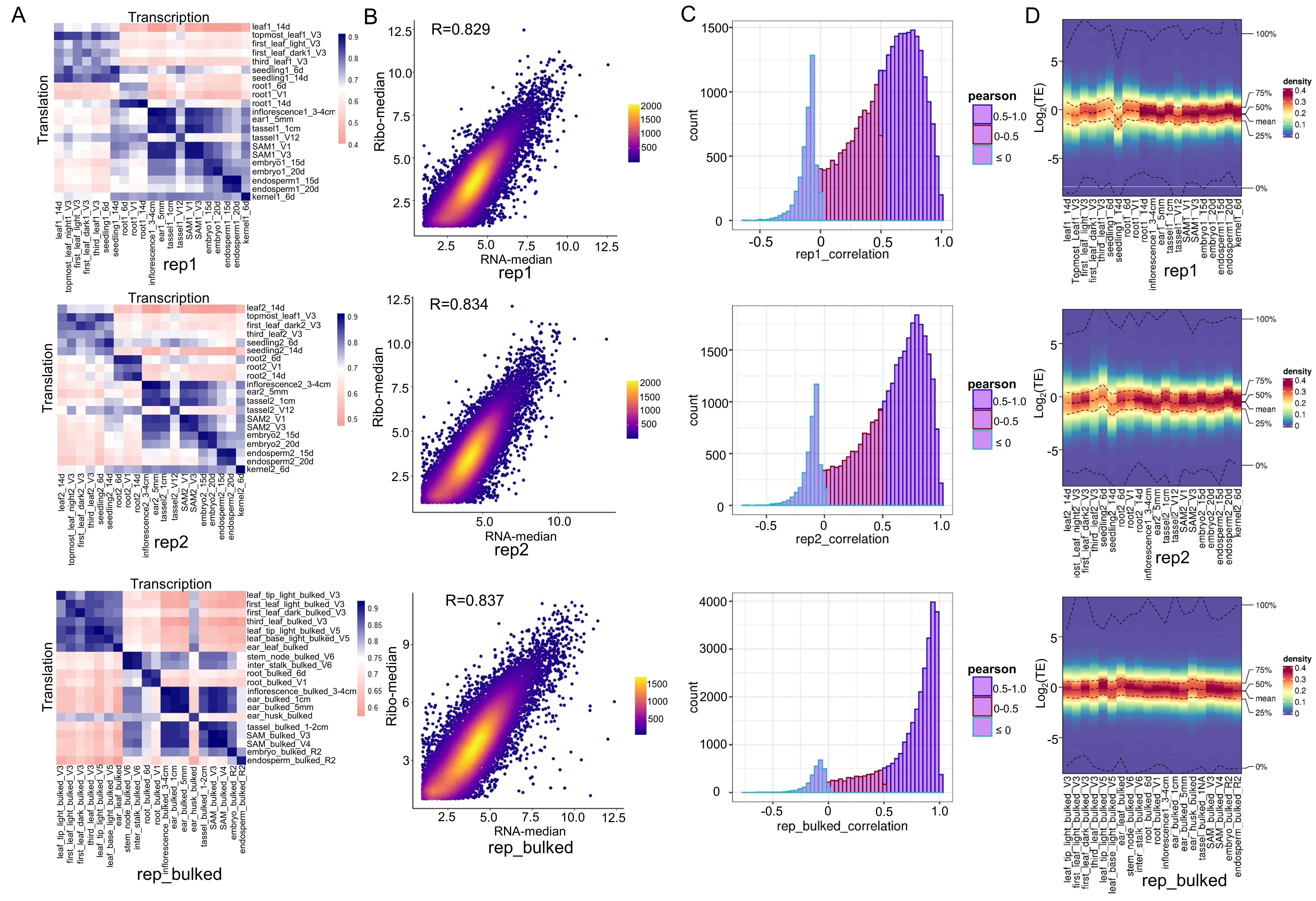


**Fig. S6 The comparison between translatome and transcriptome across all tissues or organs.**

A. Comparisons between translatome and transcriptome as performed in every two tissues using the Pearson correlation coefficient; B. Median values of translation level for each gene across all tissues were compared to the median values of transcript level for each gene across all tissues. C. Translation and transcription levels of each gene were extracted from every tissue. The comparison was performed between translation and transcript levels for each gene in the same tissues set using Pearson’s correlation coefficients. All correlation coefficients were plotted as a density plot. D. The translational efficiency (TE) in green samples showed difference comparing with other samples. Translational efficiency (TE), an important index for translation, was calculated by FPKM_Ribo-seq_ /FPKM_RNA-seq_ and used to measure the efficiency of RNA utilization


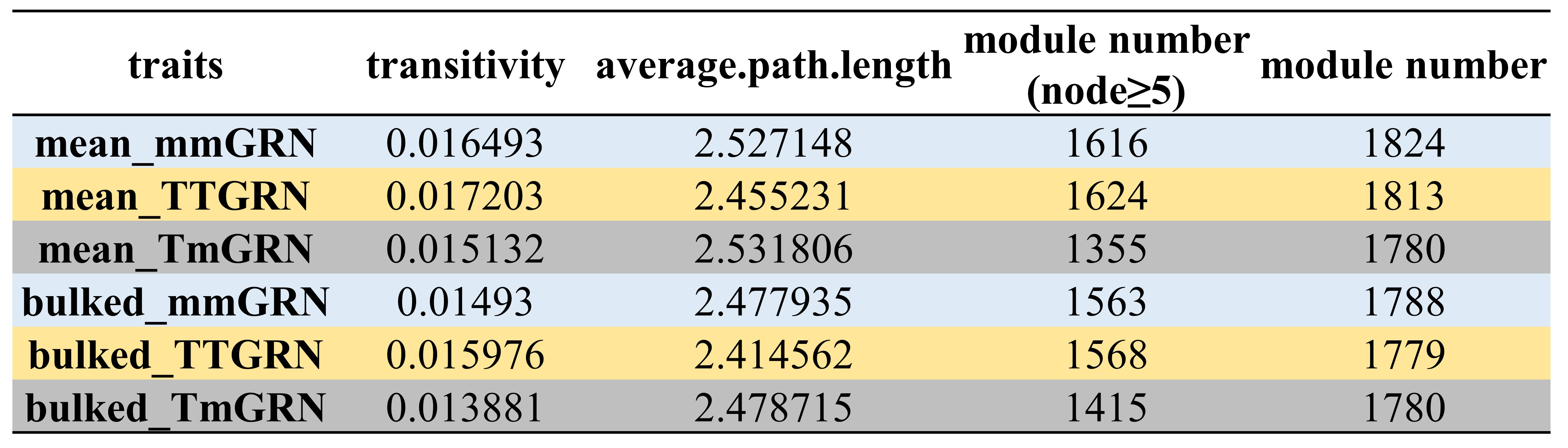


**Fig. S7 The attributes of networks including transitivity, average.path.length and module number.**


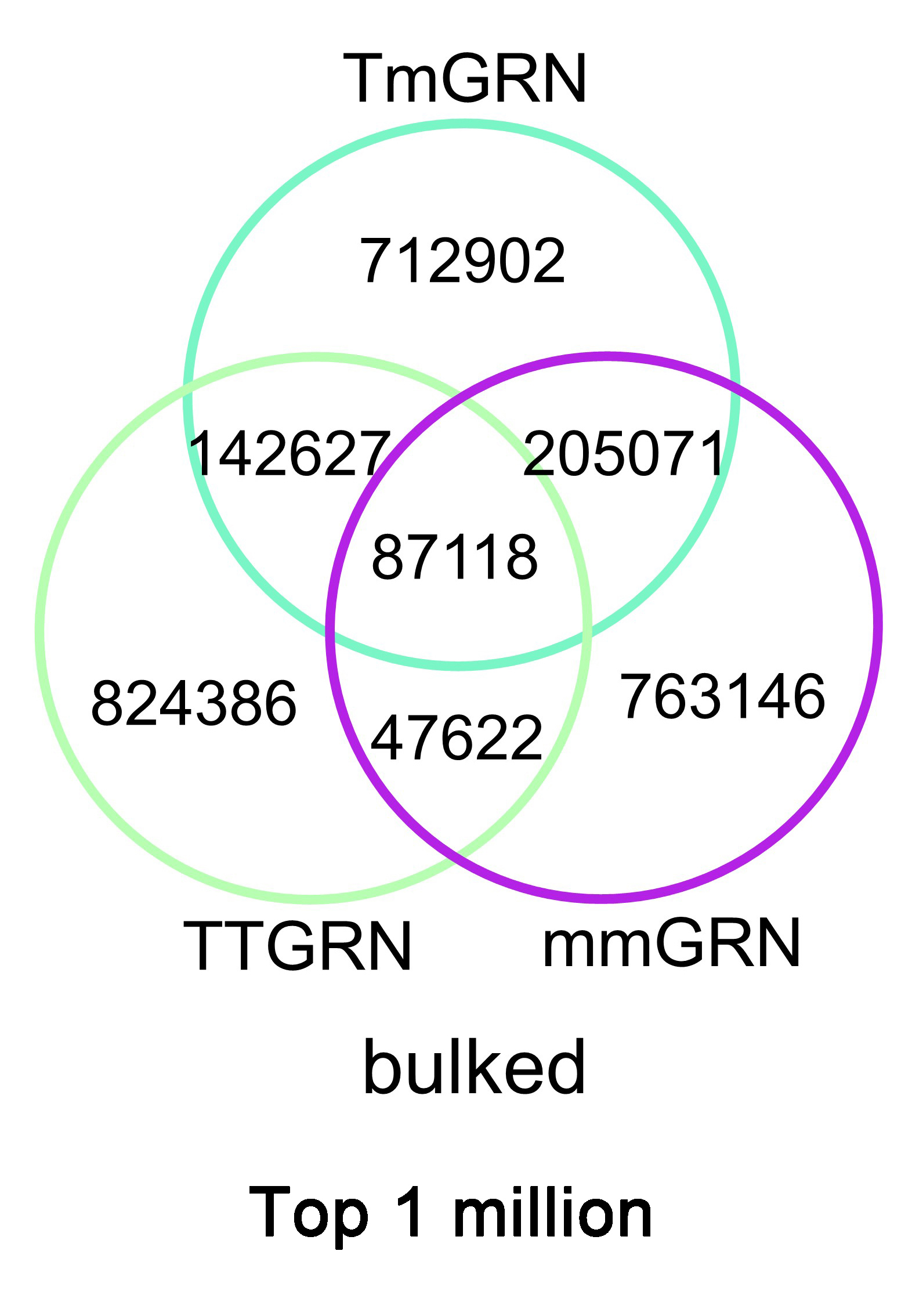


**Fig. S8 Comparison of mmGRN, TmGRN and TTGRN from the bulked data source for top 1 million edges.**


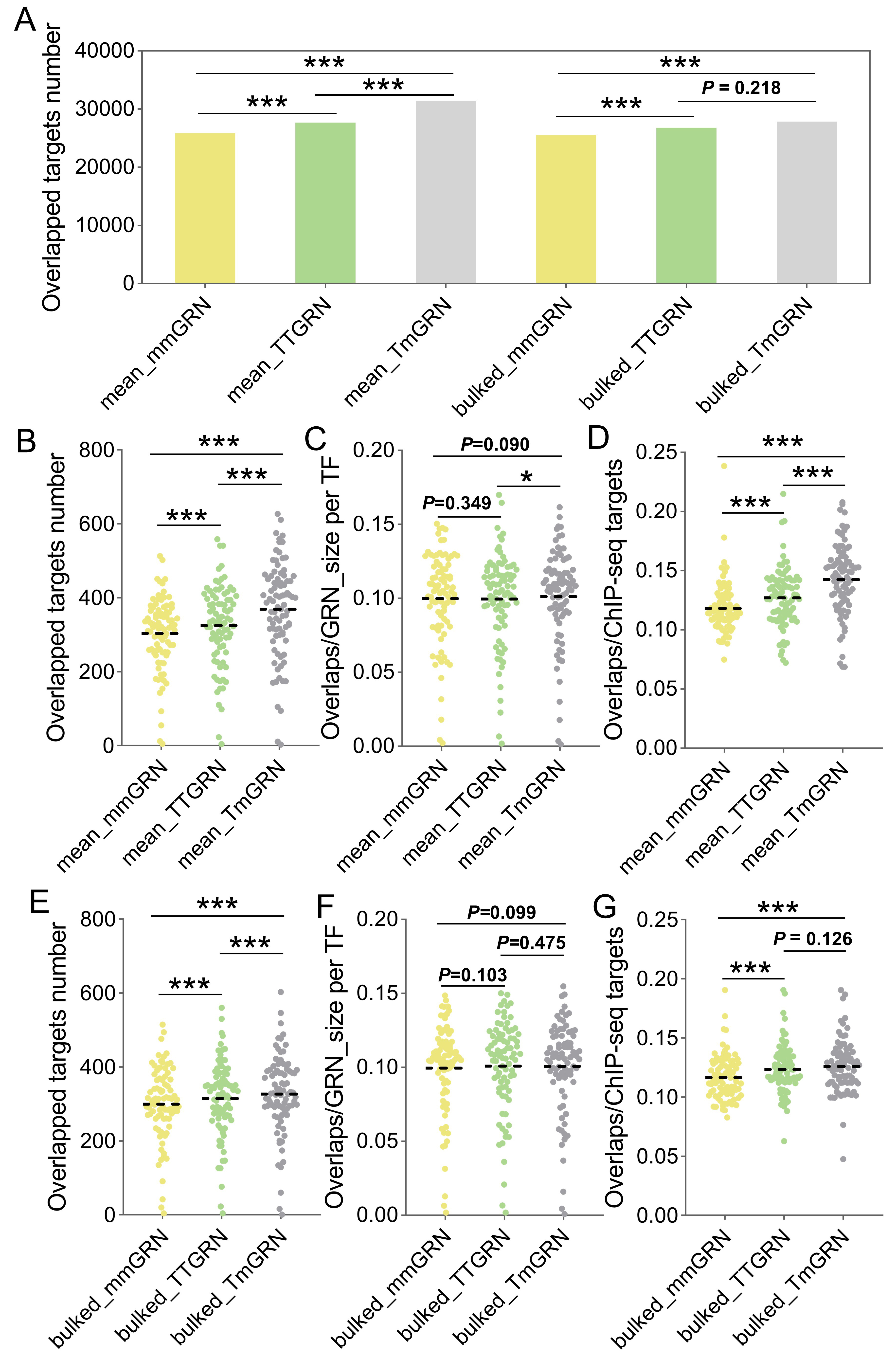


**Fig. S9 TmGRNs and TTGRNs have more overlapped targets than mmGRNs with ChIP-seq (weight ≥ 0.0003).**

A. Overlapped target number in 3 types of GRNs with ChIP-seq targtes of 86 TFs, the χ2 test was used in comparison of three types of GRNs. B and E. Overlapped target number for each TF in 3 types of GRNs across the mean and bulked data sources (Student paired *t*-test). C and F. Overlaps between GRN and ChIP-seq for each TF after normalization by GRN size of each TF (Student paired *t*-test). D and G. Overlaps between GRN and ChIP-seq for each TF after normalization by ChIP-seq target number (Student paired *t*-test). “*” represents *P* < 0.05, “***” represents *P* < 0.001.


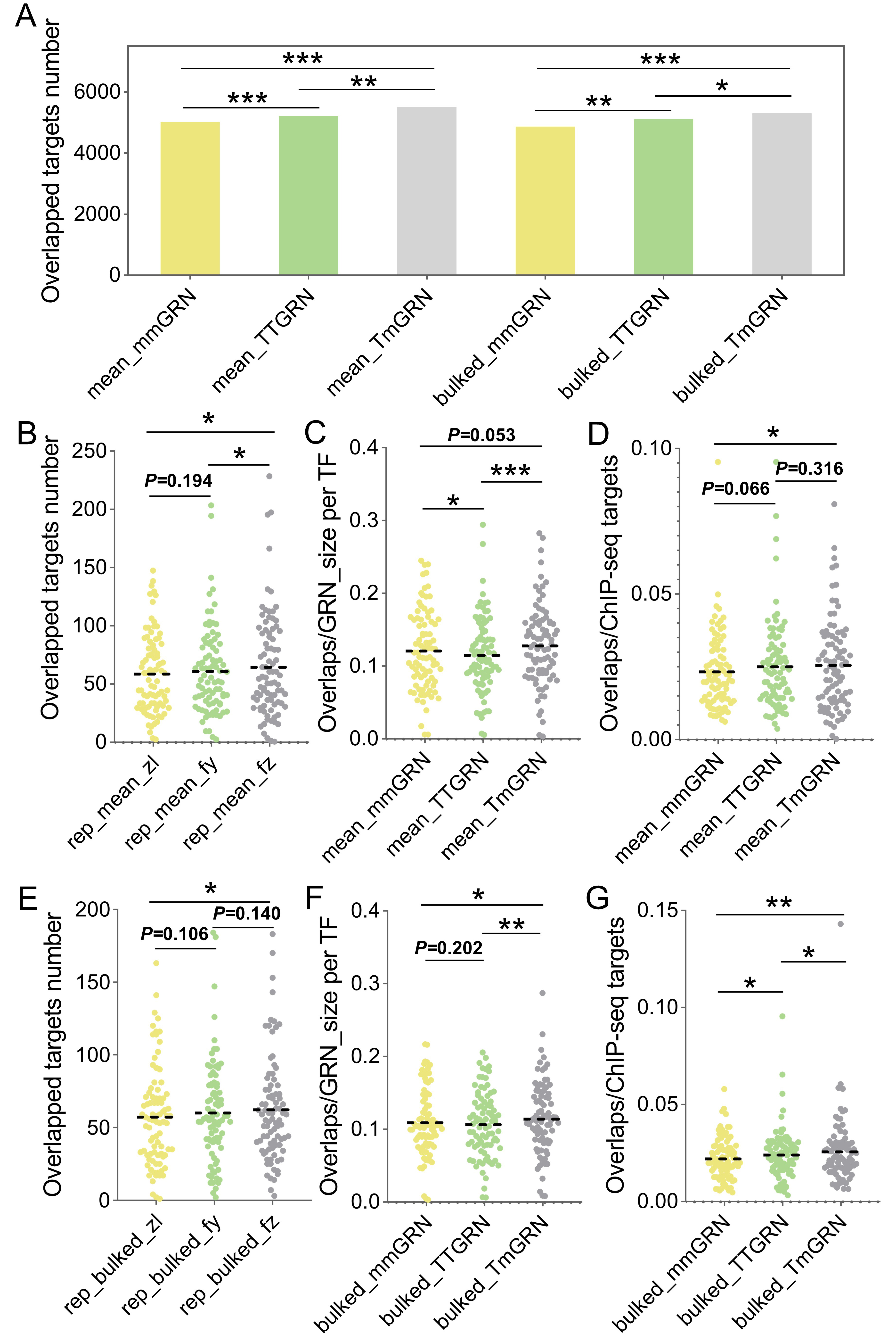


**Fig. S10 TmGRNs have more overlapped targets than other GRNs with ChIP-seq (weight≥0.01).**

A. Overlapped target number in 3 types of GRNs to ChIP-seq targtes of 86 TFs, the χ2 test was used in comparison of three types of GRNs. B and E. Overlapped target number for each TF in 3 types of GRNs across mean and bulked data sources (Student paired *t*-test). C and F. Overlaps between GRN and ChIP-seq for each TF after normalization by GRN size of each TF (Student paired *t*-test). D and G. Overlaps between GRN and ChIP-seq for each TF after normalization by ChIP-seq target number (Student paired *t*-test). “*” represents *P* < 0.05, “**” represents *P* < 0.01, “***” represents *P* < 0.001.


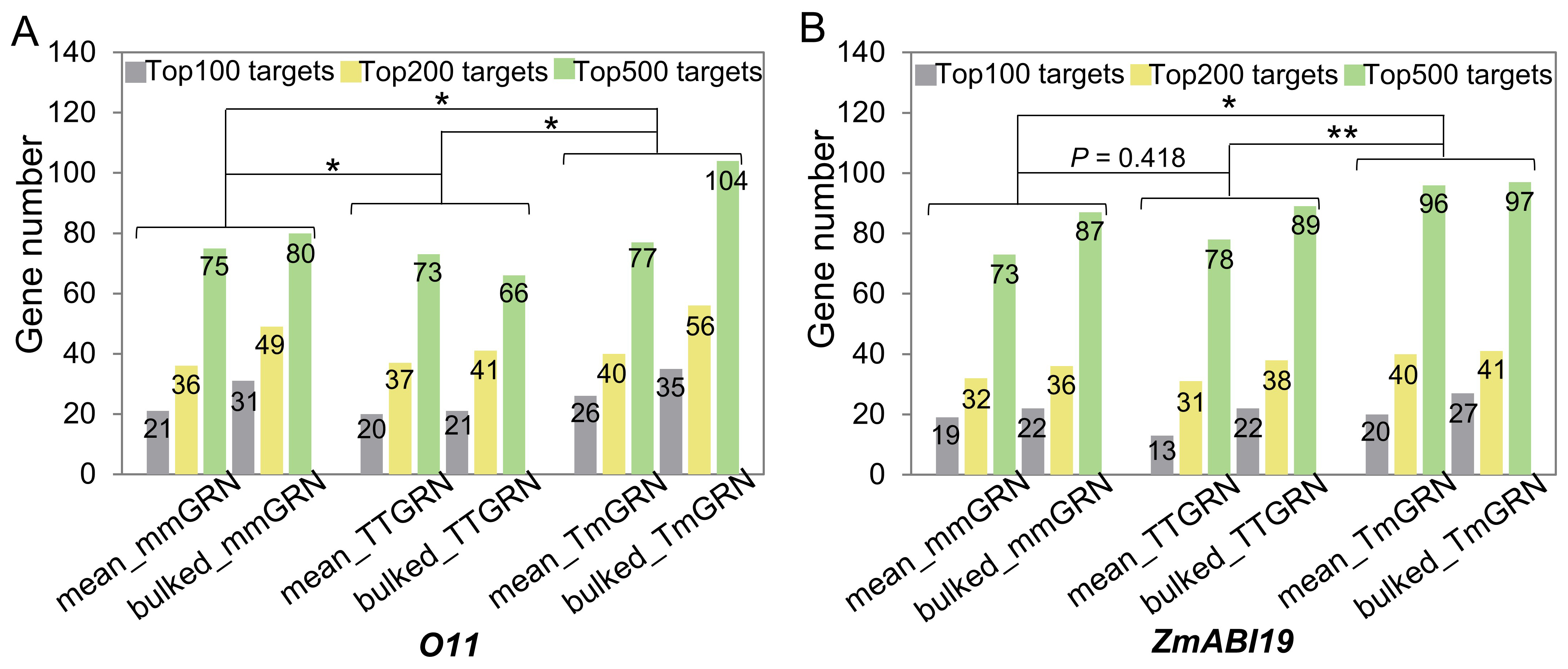


**Fig. S11 Target comparison between GRNs and ChIP-seq for *O11* (A) and *ZmABI19* (B).** The significance of difference was tested by paired Student's *t*-test, “*” represents *P* < 0.05, “**” represents *P* < 0.01.


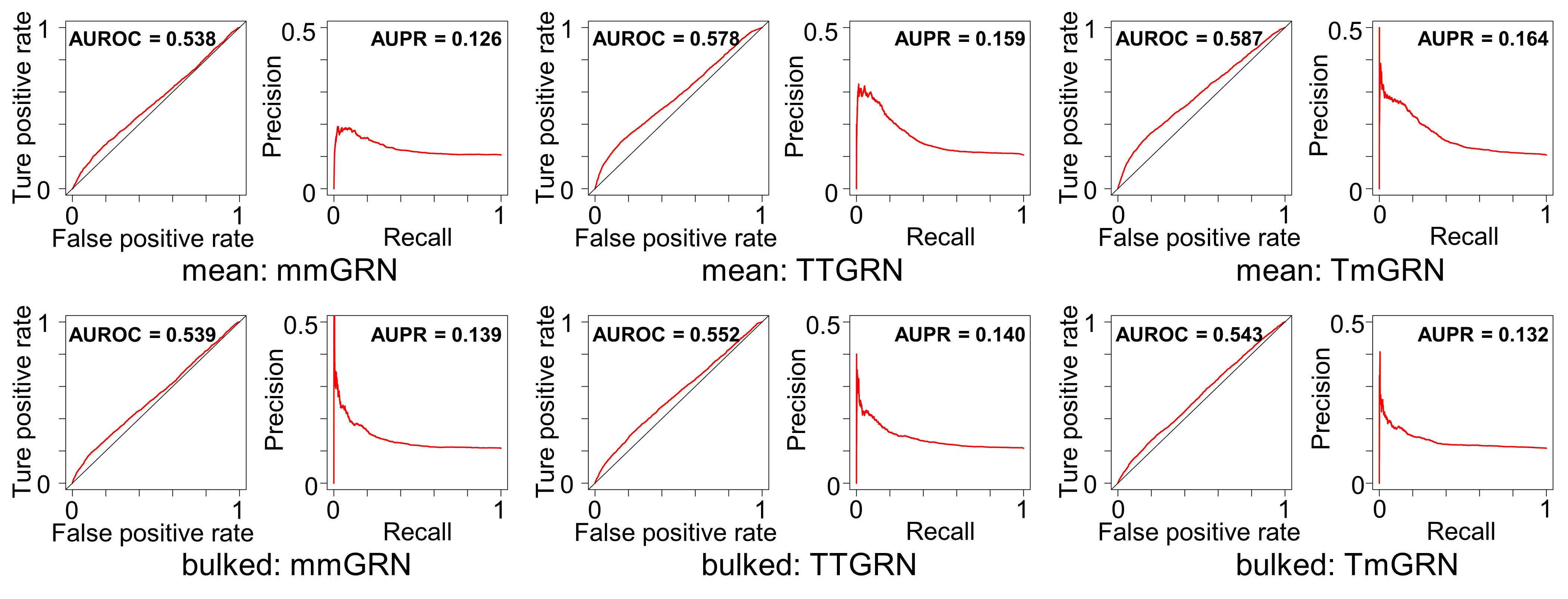


**Fig. S12 The ROC and PR values for each GRN based on the ChIP-seq targets (targets of 86 TFs as benchmark).**


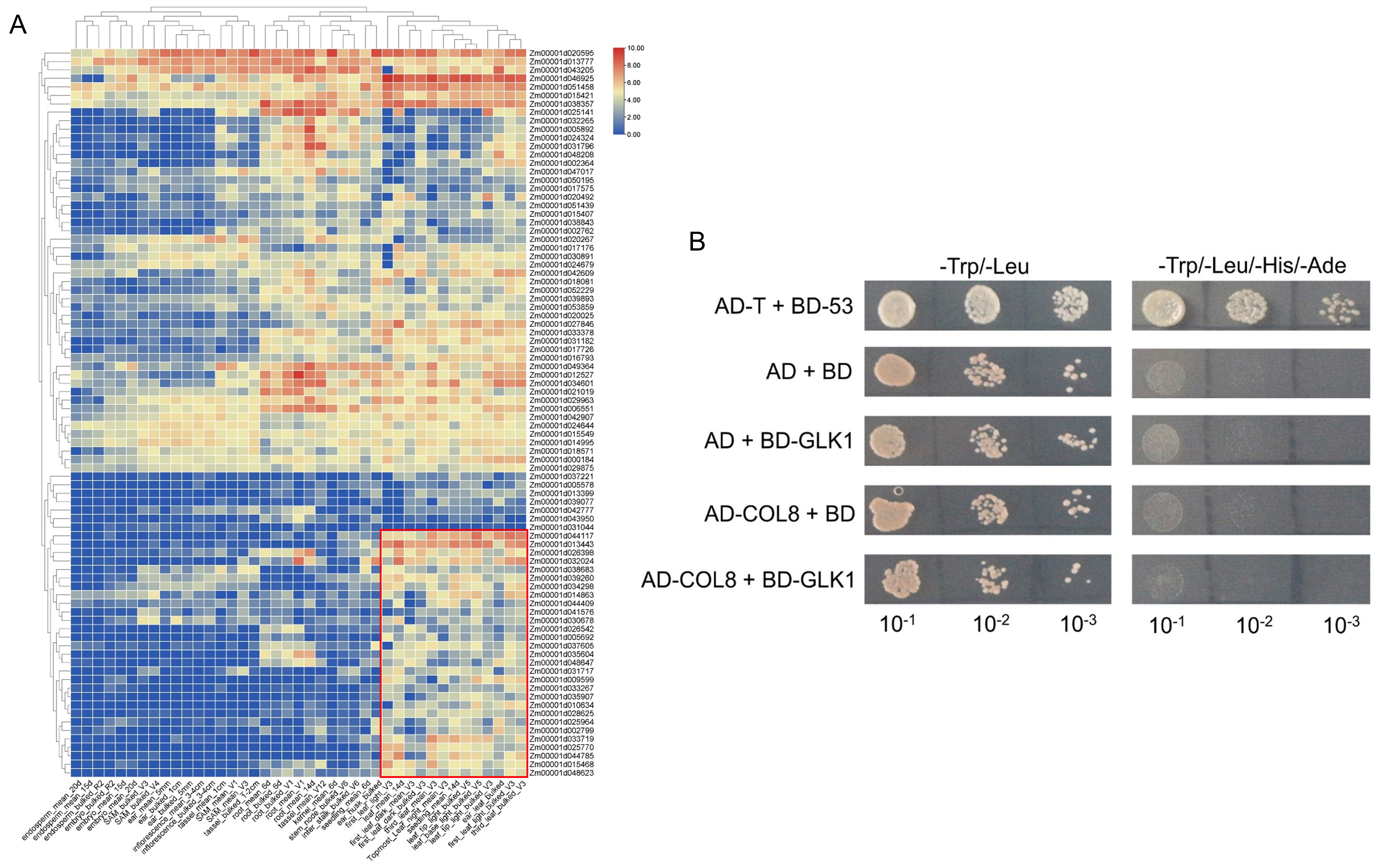


**Fig. S13 Identification of 29 TFs specifically translated in leaves (A) and detection of no interaction between *COL8* and *GLK1* (B).**


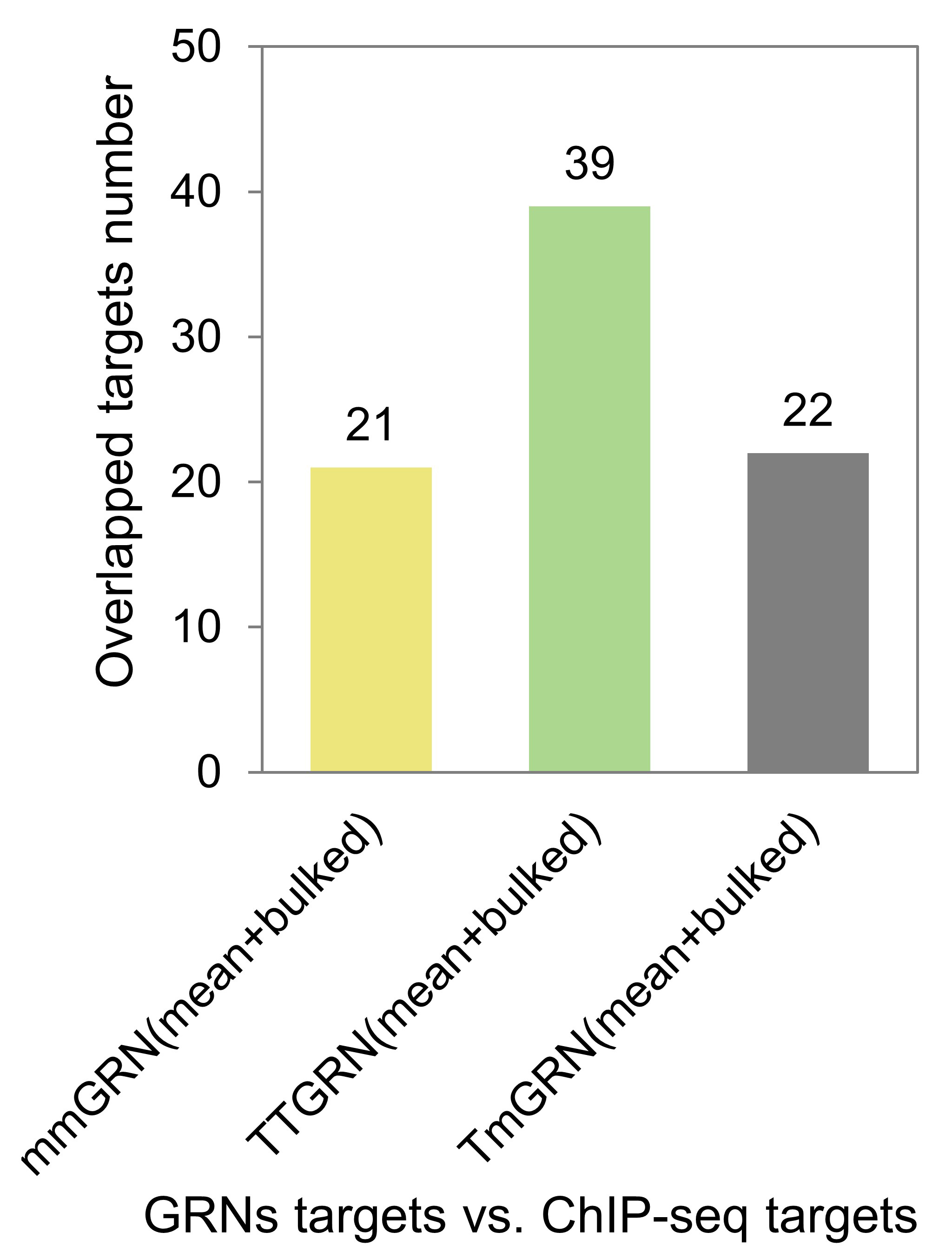


**Fig. S14 Overlap number between Union GRN and ChIP-seq targets.**


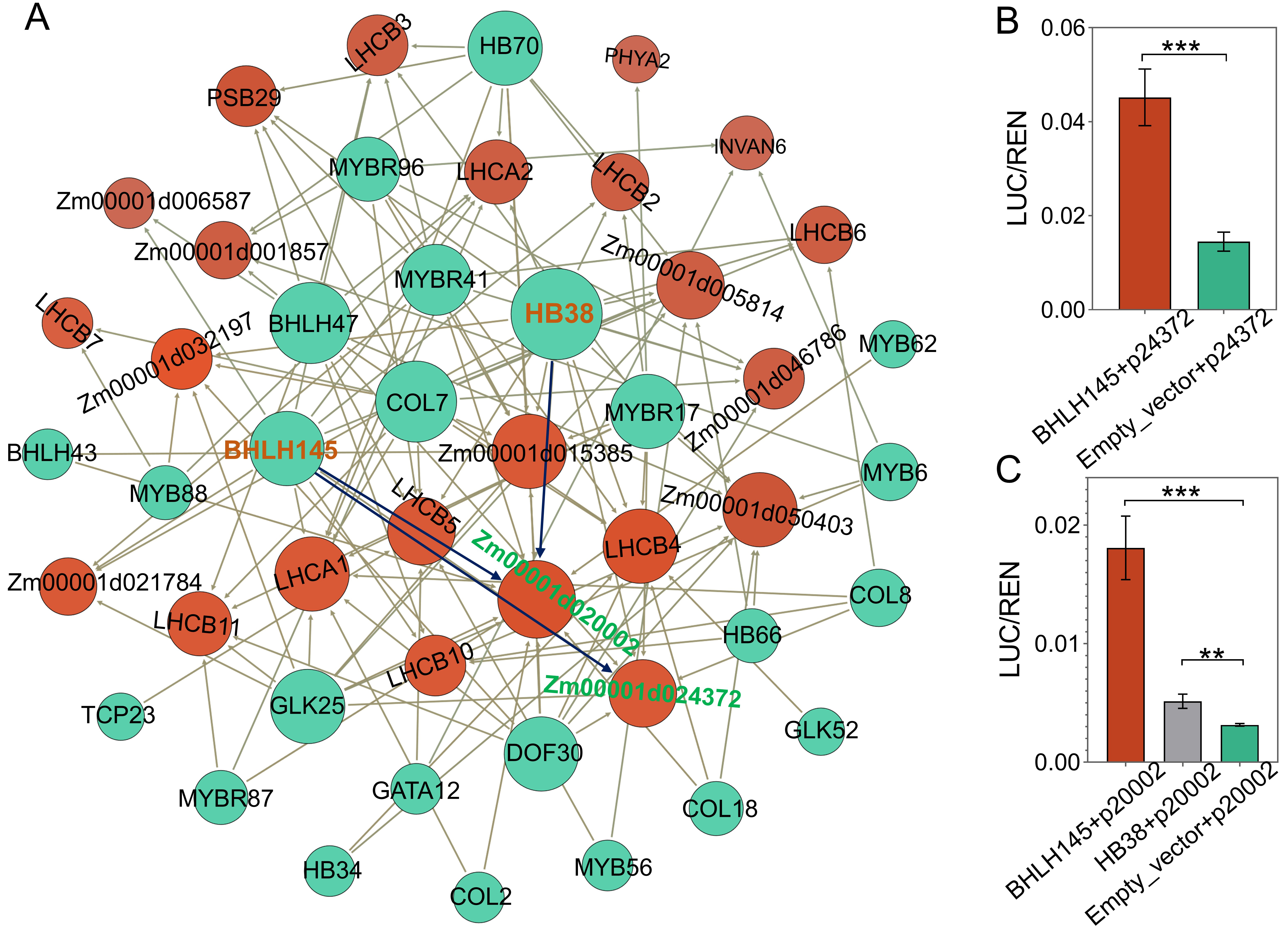


**Fig. S15 A total of 128 regulatory pairs (predicted only by GRNs) between photosynthesis genes and leaf TFs.**

A. 128 regulatory pairs predicted only by GRNs. B. A luciferase assay between *BHLH145* (TF) and *Zm00001d024372* (target). C. The luciferase assays between *BHLH145* (TF) and *Zm00001d020002* (target), and between *HB38* (TF) and *Zm00001d020002* (target). Significances of difference were calculated using the Student's *t*-test, “**” represents *P* < 0.01, “***” represents *P* < 0.001.


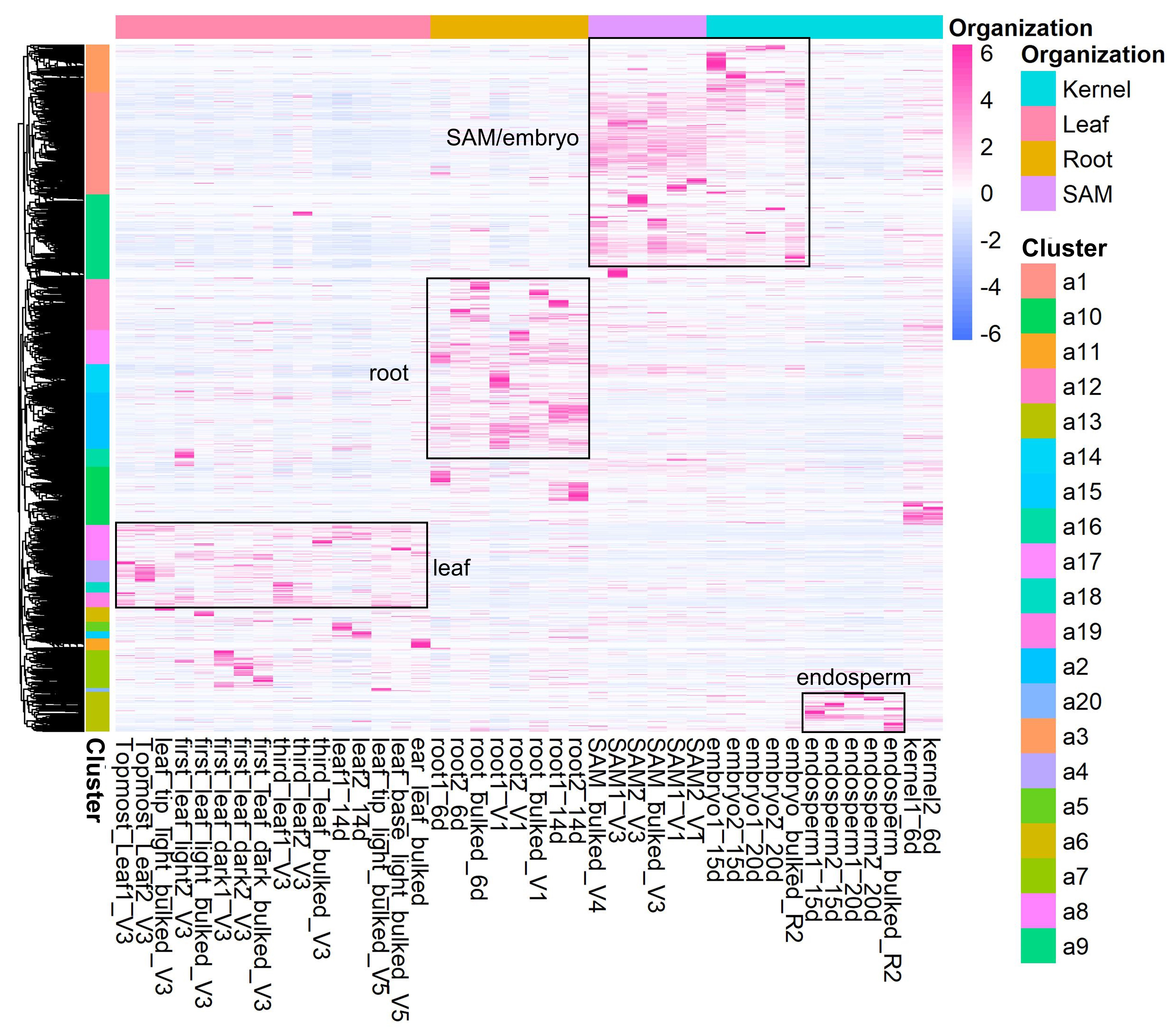


**Fig. S16 Genes specifically detected in leaf, root, SAM/embryo and endosperm at the translatome level.**


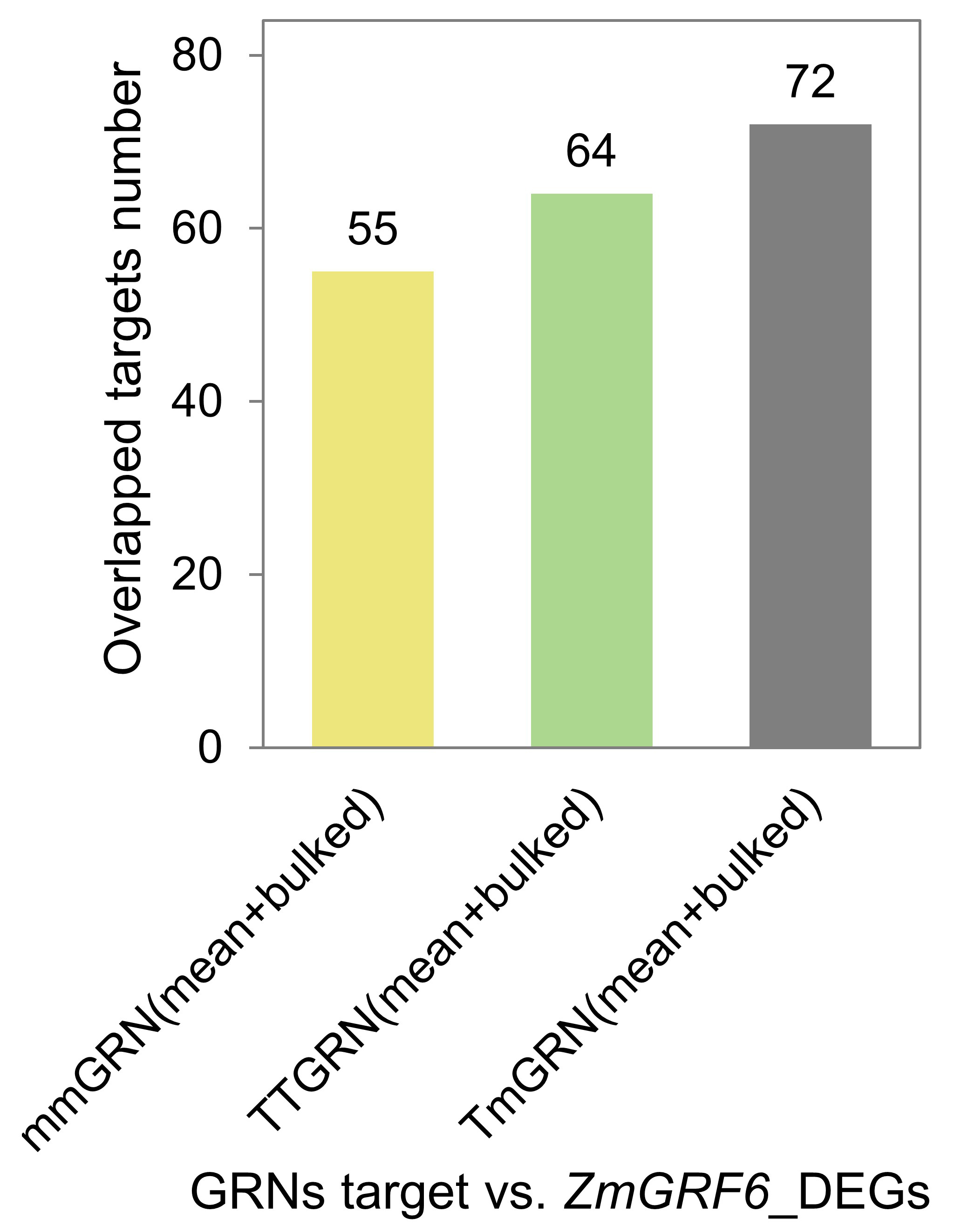


**Fig. S17 Overlapped target number between Union GRNs and DEGs.**


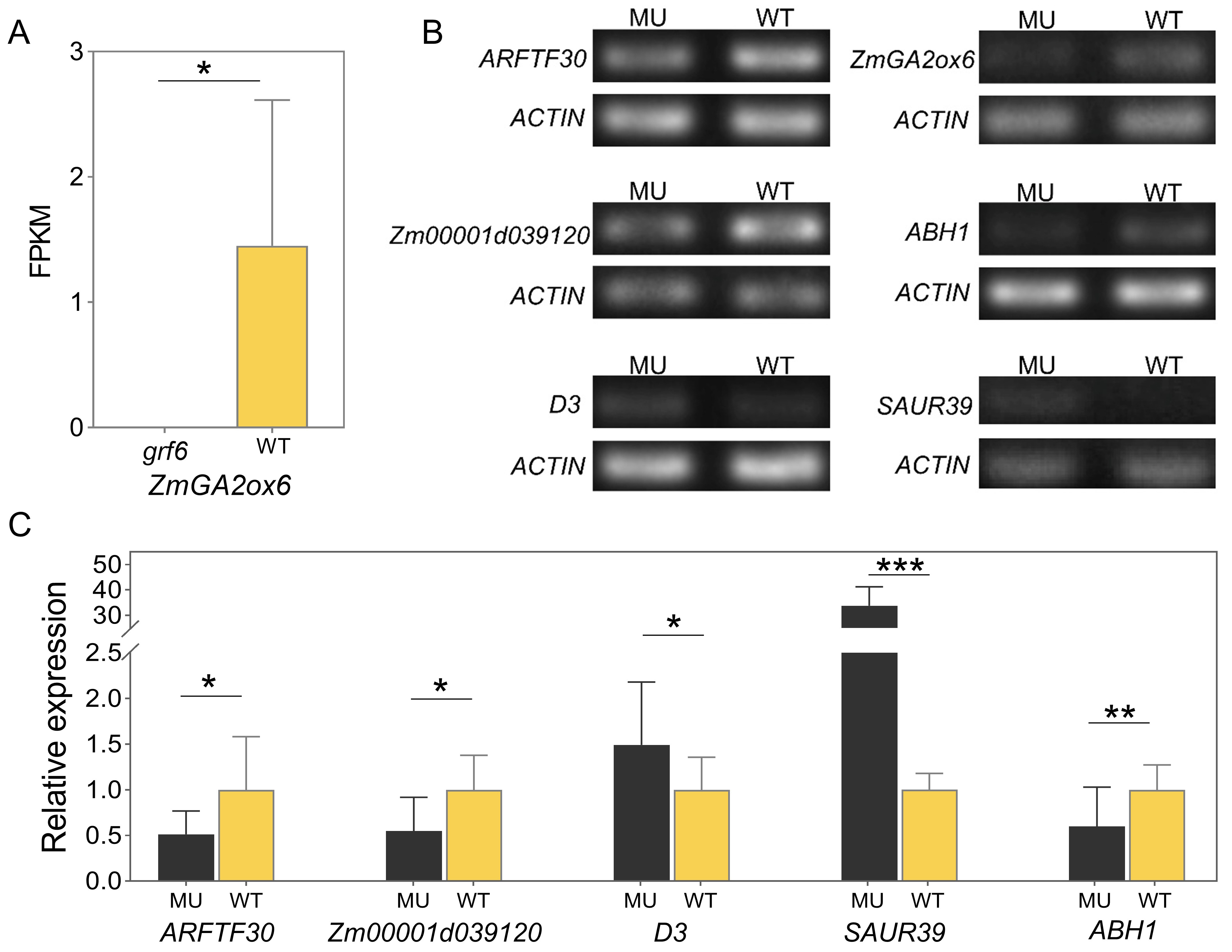


**Fig. S18 Expression Changes of the target genes in *grf6* mutant.**

A. Differential expression analysis using RNA-seq suggested that the expression of *ZmGA2ox6* was inhibited in mutant, “*” represents *P* < 0.05. B. The semi-quantitative RT-PCR was performed to verify the targets/DEGs, the same amount of RNAs (500 ng) was used to reverse transcription for mutant and WT, PCR products of maize actin gene were used to control for approximately equal concentrations of cDNA in the RT-PCR reactions. C. Real-time quantitative PCR (qRT-PCR) was performed to verify the targets/DEGs using the RNA of WT and MU.


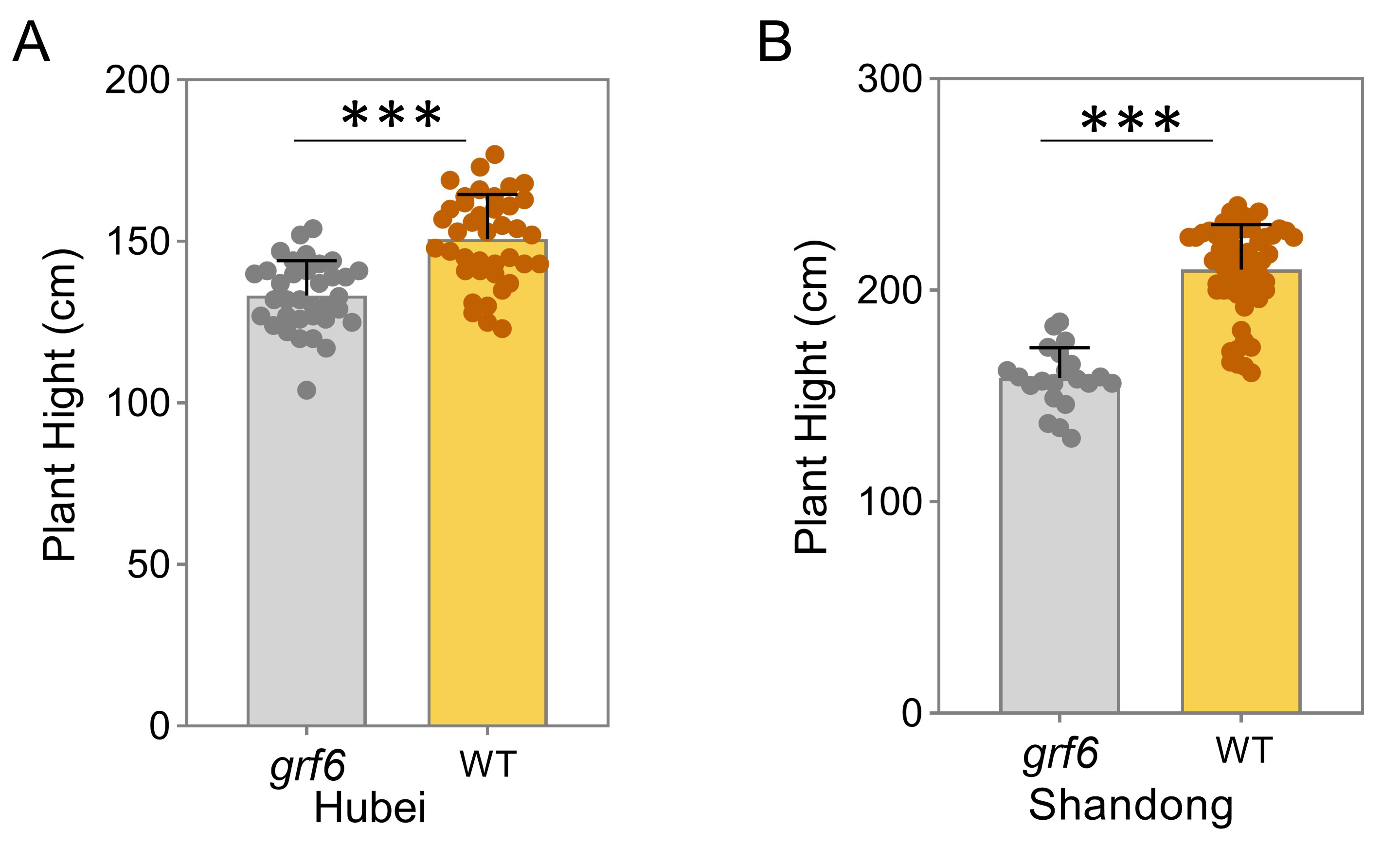


**Fig. S19 Significant difference of plant height between *grf6* mutant and WT in Hubei (A) and Shandong (B).** Significances of difference were calculated using the Student's *t*-test, “***” represents *P* < 0.001.


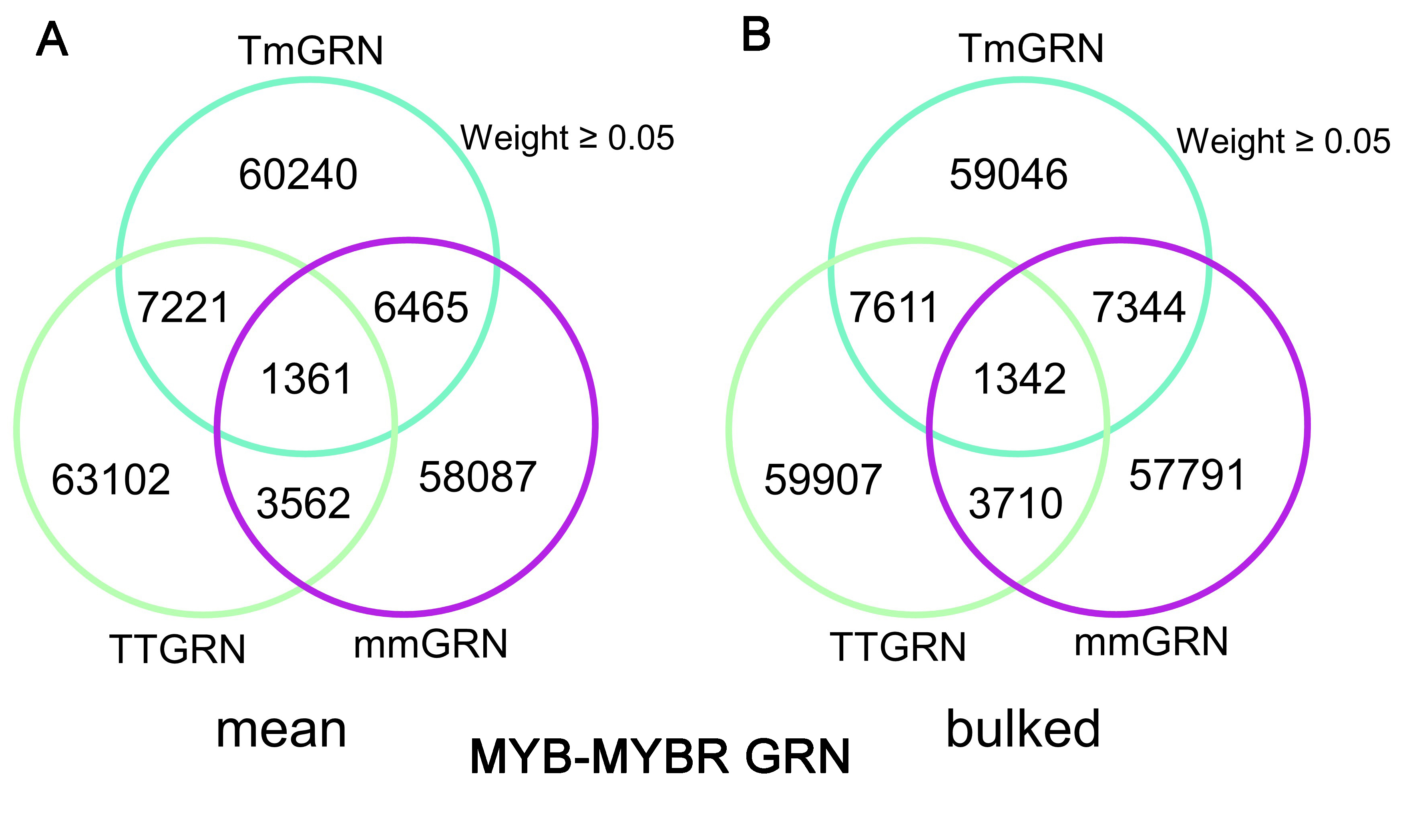


**Fig. S20 Target comparison of mmGRN, TmGRN and TTGRN for MYB-MYBR TFs across the mean (A) and the bulked (B) data sources (weight ≥ 0.05).**


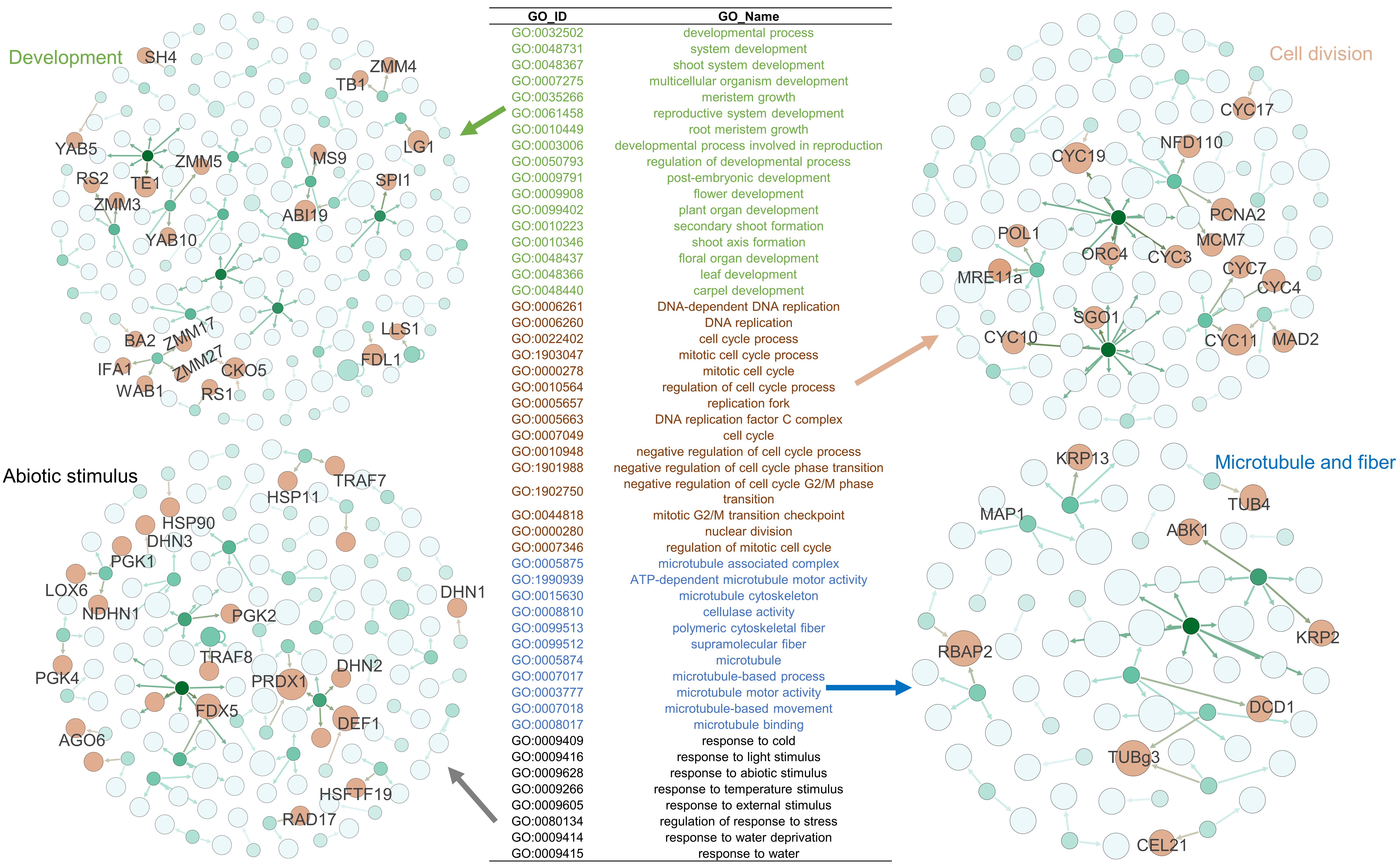


**Fig. S21 GO enrichment for the predicted targets of MYB-MYBR TFs.**


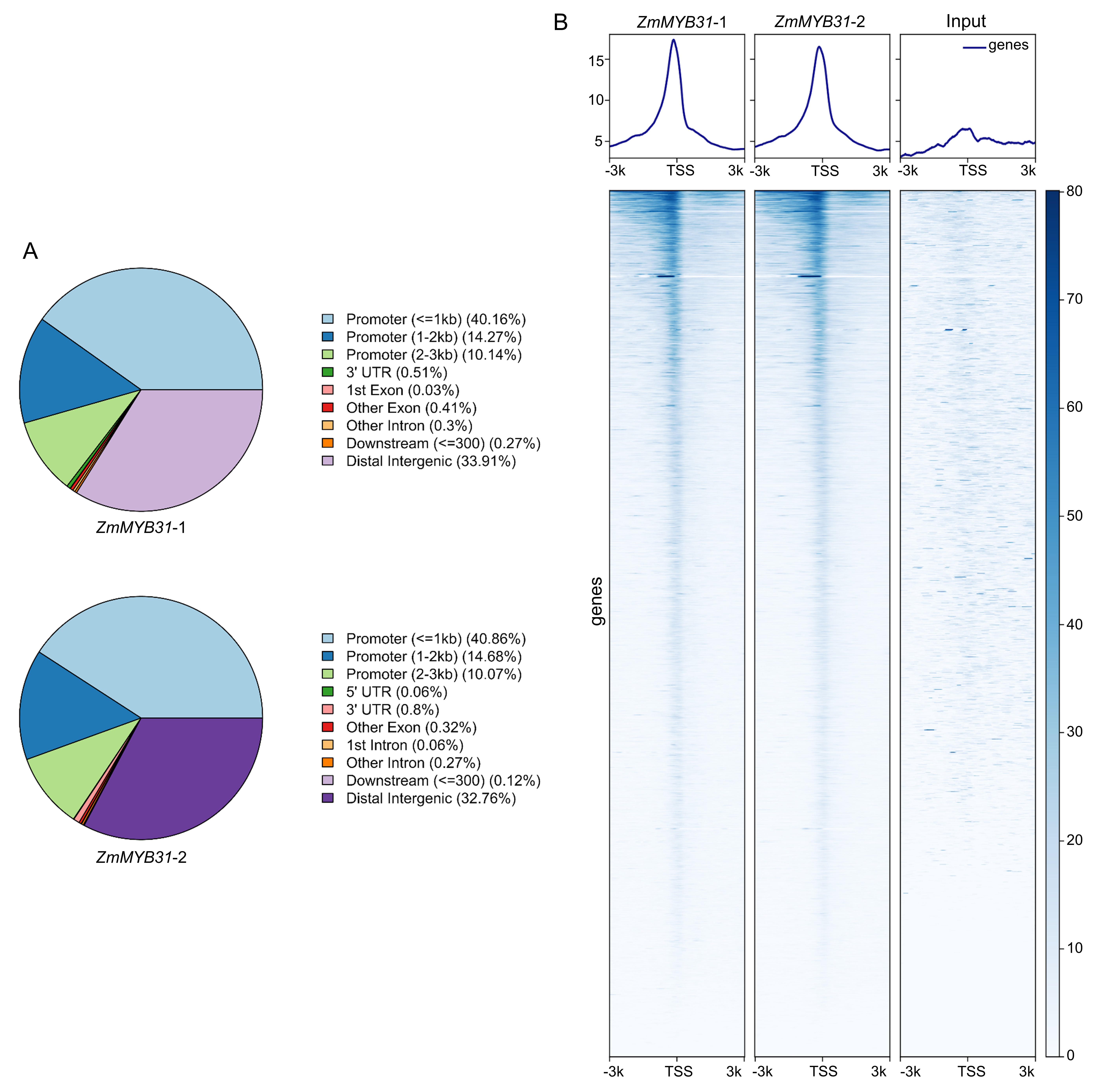


**Fig. S22 ChIP-seq was performed for the *myb31* TF.**

A. The binding site of MYB31 are mainly in promoter region. B. MYB31 proteins mainly bind to the sequence nearby TSS.


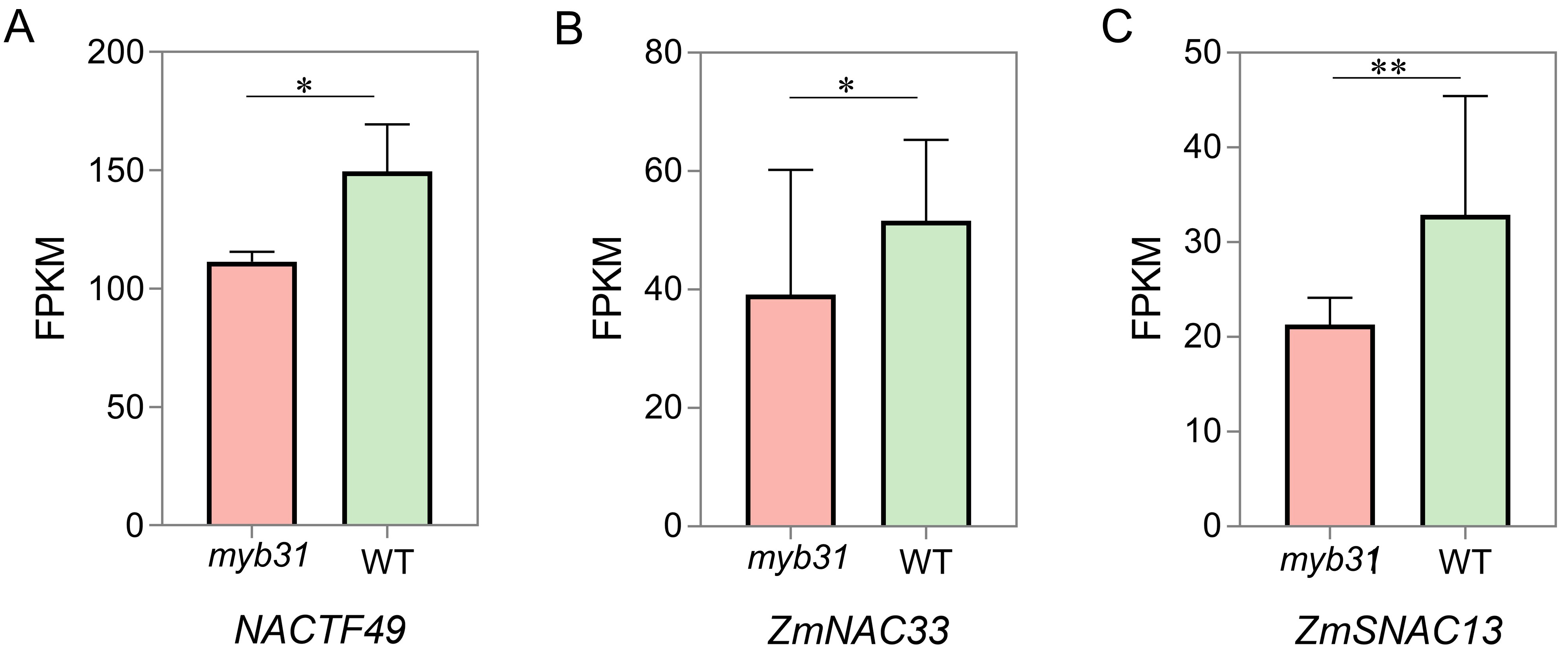


**Fig. S23 Expression-levels of *NACTF49* (A), *ZmNAC33* (B) and *ZmSNAC13* (C) were inhibited in *myb31* mutant. The three genes are all the differentially expressed genes detected by RNA-seq, “*****” represents *P* < 0.05, “******” represents *P* < 0.01.**


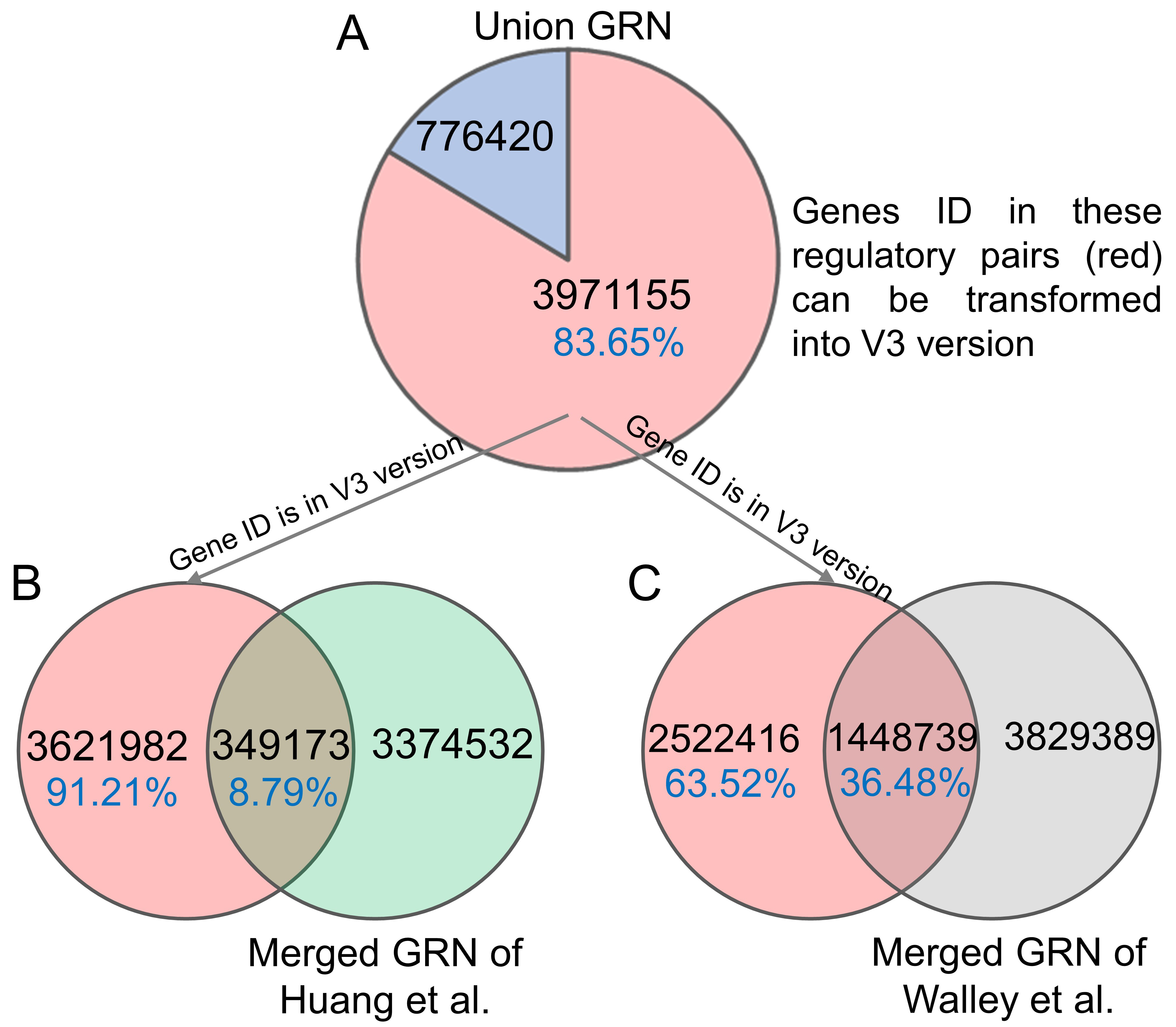


**Fig. S24 Comparisons of Union GRN in our study and the GRNs in previous studies.**

A. Genes ID in most predicted regulatory pairs (83.65%) in Union GRN can be transformed into V3 version. B. Comparison between the regulatory pairs with transformed V3 ID and the merged GRN constructed by Huang et al [1], the merged GRN was composed of four GRNs (leaf, root, SAM and seed GRN, each with top 1 million edges). C. Comparison between the regulatory pairs with transformed V3 ID and the merged GRN constructed by Walley et al [2], the merged GRN was composed of seven GRNs (each with top 1 million edges).

1. Huang J, Zheng J, Yuan H, McGinnis K. Distinct tissue-specific transcriptional regulation revealed by gene regulatory networks in maize. BMC Plant Biol. 2018;18:111.

2. Walley JW, Sartor RC, Shen ZX, Schmitz RJ, Wu KJ, Urich MA et al. Integration of omic networks in a developmental atlas of maize. Science. 2016;353:814-818.
