# Supplementary material for: A translatome-transcriptome multi-omics gene regulatory network reveals the complicated functional landscape of maize
Source: Genome Biol. 2023 Mar 29;24:60. doi: 10.1186/s13059-023-02890-4 (PMC10053466; doi:10.1186/s13059-023-02890-4)
Supplement: Supplementary file 19 — Additional file 19. Review history. [file 13059_2023_2890_MOESM19_ESM.docx]

Review History

**First round of review**

**Reviewer 1**

**Were you able to assess all statistics in the manuscript, including the appropriateness of statistical tests used?**

Yes, and I have assessed the statistics in my report.

**Comments to author:**

Zhu et al. constructed maize GRNs integrating translatome data for TF and transcriptome data for target gene expression, across many different developmental stages and tissue types, and validated some TFs using transgenic approaches. Although the approaches and information reported in the manuscript are valuable, I have major concerns regarding the data, approaches, and interpretation about the study, described as below.

Lack of replicates/statistical power: It seems like there is only one rep for the newly collected 20 tissues (Line 124). The good agreement between the previous two reps and the new bulked rep for a subset of the tissues does not remove the need of having multiple replicates to draw statistically solid conclusion. For the mutant study focused on single genes, only two reps are included, which also limited the statistical power of the study.

On a similar note, GENIE3 is most powerful when the input omics data used for training are in the number of hundreds to thousands, for it to predict the regulatory relationship between thousands of predictors (TFs) and the target genes. The input data here is on the low end (20-21 translatome/transcriptome), leading to the concern that the prediction might be low confidence. I am curious if we use the 86 TFs and their ChIP-seq target as benchmark, how does the GENIE3 perform (accurancy, PR/ROC curve, or F1 score etc)

Some interpretations are not well supported. For example, on Line 138: "As expected, differentiating tissues had more detectable genes than more highly differentiated tissues at both the translatome and transcriptome levels ".. needs better explanation and support. I see interspersed tissue types in Figure 1B. Line 150 "finding greater numbers of SEGs in most reproductive organs (tassel, kernel and ear) and a few vegetative organs (root and stem) than in other tissues…" also needs better explanation and support, as the least No. of SEGs is observed in reproductive organs (flower). And line 360, Figure 6B: it does not look like most target genes show similar expression pattern with GRF6. In Line 283, how do we know that the 101 functional genes are associated with kernel size?

In general, for a computational heavy study like this, the description of data analysis can be improved with providing more details. The manuscript needs language editing for clarity and accuracy, especially for such a complex data analysis. A data analysis flow chart might help too. In the result text, it is not explained how GRN are constructed. In line 200, it is not clear why the four datasets are used, other than merging the means of the rep1 and rep2 with the bulk data to form a bigger panel of input data. This also led to problems later in the manuscript that it is unclear which GRN (from which dataset) was used (for example line 282). In Figure 3B, how the results for 86 TFs are synthesized to form the plot? In Figure 3F, TP and FP refers to the motifs detected, or whether the target gene promoters have the motifs or not? In line 221, it was mentioned that union networks and intersected (overlapped) high confidence networks were created, how are these two networks used later? FigS3 A B and C: please explain in detail what was being used in the correlation.

One main point made in the manuscript is that the tmGRNs built with translatome data is better than the intraGRNs, and this needs to be better supported. It seems that the top 1 million edges from each GENIE3 output was used. Is the threshold edge weight/confidence comparable between different types of GRNs? It seems that the tmGRNs have better weight among the top 1 million edges, suggesting that the tmGRNs are higher confidence GRNs than other GRNs anyway. Therefore, it is less surprising that it has bigger number of overlap with the ChIP-seq results. If the same edge confidence threshold was used to trim different GRNs, will tmGRNs still have better overlap with the TF ChIP-seq results than the other GRNs (after normalized to GRN sizes)? In the current manuscript, is the difference in overlaps with ChIP-seq between TmGRNs and mmGRNs significant (lines 241-246, Figure 3B&3C)? The only significant difference between tmGRNs and mmGRNs of this part is shown for Fig 3D, limited to the four TFs, but how are these four TFs selected? In the last few sections validating the GRNs (with photosynthesis genes, MYBs and GRF), is the tmGRN outperforming the other GRNs?

To validate the GRN prediction, leaf TFs and photosynthetic genes were focused on. In Figure 4B, is this overlap significant considering the background is limited to the interactions between photosynthesis genes and leaf TFs? How are the four TFs selected (line 298)? LHCB7 and PSB29 are already shown to be the direct targets of TFs by previous ChIP-seq, therefore, the luciferase assay validating the gene activation is less impressive in proving the predicting power of the GRNs. To validate the predicting power of the GRNs, it is better to pick the genes from the 241 gene list in Figure 4B to show that GRNs can identify new regulatory relationship between TFs and photosynthesis target genes.

To validate the GRN prediction and to show that GRNs could be used to drive new discovery, GRF and MYB TFs were focused on. How are the specific GRF and MYB selected? Figure 6A and 7A: are these all the targets predicted by GRNs? Which GRNs (Tm, mm, and rep1, rep2, etc) was used? Line 366 on GRFs: is the overlap overall significant thus proving the power of the GRNs in predicting target genes? Similarly, for MYB, do the regulated targets of MYB31 and the bound targets detected by cut-and-tag overlap significantly with the GRN targets? Besides, it should be mentioned that the bound targets were performed in protoplasts not in whole plants.

Figure 8A and 8C: what is the significance level of the difference between WT and mutant?

Minor points:

Overall texts are too small to read in figure.

Line 166: please briefly explain the meaning of TE for audience who is not very familiar with the term.

The part on GRFs (line 331-342) goes better with the next result section.

**Reviewer 2**

**Were you able to assess all statistics in the manuscript, including the appropriateness of statistical tests used?**

Yes, and I have assessed the statistics in my report.

**Comments to author:**

Building gene regulatory networks with high accuracy will be very useful to the plant research community. In this ms, the authors collected the translatome and transcriptome datasets and constructed a set of multi-omics GRN, and tested its reliability with additional experiments. Overall, the ms is very interesting, and I have only several scientific comments.

1. The authors concluded that" inter-omics GRNs outperform intra-omics GRNs". And seems to emphasize that TmGRNs outplayed the mmGRNs in the following ms. I am wondering how TTGRN compared with TmGRN. From Figure 2D, the networks constructed from overlapping edges among mmGRNs, TmGRNs, and TTGRNs provided a better GRN, why the authors did not focus on this high-confidence network?

In line 275, "These results indicate that inter-omics GRNs outperform traditional intra-omics GRNs for demonstration of the regulatome in maize." I would suggest the authors take a look at the TTGRNs and give an explanation if it is not as good as TmGRNs.

2. Line 200-202, how did the authors identify the 1,764, 201 1,788, 1,824, and 1,787 TFs at both translatome and transcriptome levels as "regulators"? it is better to give a short description in the text.

3. Line 237-240, "We found that our different GRNs showed significantly more overlap with ChIP-seq data than a control GRN (randomly generated network with the same TFs and targets) (Figure 3B)." It is better to explain what is the "randomly generated network" and what kind of "comprehensive comparison" were done between GRNs and ChIP-seq results.

4. Line 279-283, the authors described using 11 well-known kernel-related TFs to extract the kernel-related GRN sub-networks. How many tissues/stages-specific edges/sub-networks did the authors identify? I suggest the authors provide a link to access these GRNs and all the GRNs that have been built in this ms, especially the edges overlapped among the three GRNs. In this way, this dataset could greatly help the whole maize community.

5. Line 294, what's the proportion of these 23 LHC-like genes in all LHC-like genes in maize?

**Authors Response**

**Point-by-point responses to the reviewers’ comments:**

**Reviewer #1:** Zhu et al. constructed maize GRNs integrating translatome data for TF and transcriptome data for target gene expression, across many different developmental stages and tissue types, and validated some TFs using transgenic approaches. Although the approaches and information reported in the manuscript are valuable, I have major concerns regarding the data, approaches, and interpretation about the study, described as below.

*Reply:* *We really appreciate your careful reading and evaluation of our work, and the valuable comments on our manuscript. We have revised the manuscript according to your suggestions. Please see our point-by-point answers below.*

1. Lack of replicates/statistical power: It seems like there is only one rep for the newly collected 20 tissues (Line 124). The good agreement between the previous two reps and the new bulked rep for a subset of the tissues does not remove the need of having multiple replicates to draw statistically solid conclusion. For the mutant study focused on single genes, only two reps are included, which also limited the statistical power of the study.

*Reply:* *We appreciate this comment. The translatome data for the two replicates and the newly collected tissues were all produced by us, making this dataset the largest translatome datasets in the maize community to date.*

*For the newly collected tissues: We collected three replicates per tissue. Because of the high cost of Ribo-seq and RNA-seq for massive samples, equal amounts of the three replicates were combined to obtain one bulk sample that was analyzed by Ribo-seq and RNA-seq. We hoped to reduce variation by mixing multiple replicates. We compared some analyzed results produced by bulk sample to those of the previous two replicates (including correlations in Fig. S2 and Fig. S6, network traits in Fig. S7, comparison in Figs. S9-S12), which showed similar tendency compared to the two reps. In addition, we also wished the variation from the bulked samples would be lower when combining the previous two replicates in GRN construction.*

*For the mutant study focused on single genes (GRF6): Indeed, we only collected and analyzed two replicates (RNA-seq) in our work on GRF6. To validate the statistical power and accuracy in identifying deferentially expressed genes (DEGs), we performed* ***semi-quantitative*** *RT-PCR for six targets/DEGs. The same amount of RNAs (500 ng) was used to reverse transcription for mutant and WT, PCR products of maize actin gene were used to control for approximately equal concentrations of cDNA in the RT-PCR reactions. ARFTF30, Zm00001d039120 (encoding an Auxin-like 1 protein), ZmGA2ox6 and ABH1 showed lower expression, while D3 and SAUR39 showed increased transcript levels in the mutant. These expression changes are consistent with those obtained by RNA-seq, suggesting the accuracy of DEGs from RNA-seq using the two replicates in our study. The semi-quantitative RT-PCR results are shown in* *Fig. S20B and below:*

Fig. S20 Validation by semi-quantitative RT-PCR of six targets/DEGs. The same amount of RNAs (500 ng) was used to reverse transcription for mutant and WT, PCR products of maize actin gene were used to control for approximately equal concentrations of cDNA in the RT-PCR reactions.

2. On a similar note, GENIE3 is most powerful when the input omics data used for training are in the number of hundreds to thousands, for it to predict the regulatory relationship between thousands of predictors (TFs) and the target genes. The input data here is on the low end (20-21 translatome/transcriptome), leading to the concern that the prediction might be low confidence. I am curious if we use the 86 TFs and their ChIP-seq target as benchmark, how does the GENIE3 perform (accuracy, PR/ROC curve, or F1 score etc)

*Reply:* *Thank you very much for the helpful suggestions.*

*Regarding your concern about sample size of the input data for GENIE3: Because of the high cost and sophisticated experimental procedure, we only collected translatomic data for 20 maize tissues, which still represents the largest Ribo-seq dataset in plants. The sample size (20/21 tissues) of input data in this study using GENIE3 might be acceptable and was close to that in a previous study that only used the transcriptome and proteome of 23 tissues for the construction of GRNs (**Walley et al, Science, 2016, PMID: 27540173). As the integration of multiple omics datasets was shown to greatly improve the predictive power of GRN (Walley et al, Science, 2016), we combined two omics (transcriptome and translatome) data types to improve our prediction power, reduce the false positive rate, and construct a more comprehensive regulatory network. In addition, we discussed how to improve the confidence of prediction in the Discussion section, providing some suggestions (Lines 570-583 in revised manuscript).*

*We used the 86 TFs and their ChIP-seq data as* *a benchmark to test GENIE3 performance, using the PR/ROC curve that are shown in Fig. S13 and Comments Table 1. The area under the ROC curve (AUROC) ranged from 0.538 to 0.588, compared to 0.500 for random predictions (Lines 263-270 in revised manuscript). These results are consistent with the results of a previous study that constructed multiple tissue-specific GRNs in maize (Huang et al, 2018, PMID: 29879919). The ChIP-seq data for 86 TFs as performed in protoplasts, which may affect the PR/ROC curve.*

Fig. S13 ROC and PR values for each GRN based on the ChIP-seq targets.

**Comments Table 1. ROC and PR values for all GRNs.**

|  | **mmGRN** | | **TTGRN** | | **mmGRN** | |
| --- | --- | --- | --- | --- | --- | --- |
|  | **AUROC** | **AUPR** | **AUROC** | **AUPR** | **AUROC** | **AUPR** |
| **Rep 1** | 0.543 | 0.134 | 0.574 | 0.165 | 0.579 | 0.166 |
| **Rep 2** | 0.558 | 0.153 | 0.588 | 0.175 | 0.586 | 0.168 |
| **mean** | 0.538 | 0.126 | 0.578 | 0.159 | 0.587 | 0.164 |
| **bulked** | 0.539 | 0.139 | 0.552 | 0.14 | 0.543 | 0.132 |

3. Some interpretations are not well supported. For example, on Line 138: "As expected, differentiating tissues had more detectable genes than more highly differentiated tissues at both the translatome and transcriptome levels ".. needs better explanation and support. I see interspersed tissue types in Figure 1B. Line 150 "finding greater numbers of SEGs in most reproductive organs (tassel, kernel and ear) and a few vegetative organs (root and stem) than in other tissues…" also needs better explanation and support, as the least No. of SEGs is observed in reproductive organs (flower). And line 360, Figure 6B: it does not look like most target genes show similar expression pattern with GRF6. In Line 283, how do we know that the 101 functional genes are associated with kernel size?

*Reply: Thank you for raising this concern.*

***Regarding concern in Line 138 in last version:*** *We have corrected our description and given a* *better explanation in lines 138 to 142 in the revised manuscript.*

*The new description is as follows:“In vegetative tissues, the roots and stems expressed more genes than leaves and endosperms at both the translatome and transcriptome levels (Fig. 1B, Additional file 1: Fig. S5A-5D). Reproductive tissues including ear, tassel, inflorescence, embryo and kernel expressed more genes than leaves, but fewer than roots (Fig. 1B, Additional file 1: Fig. S5A-5D).” To support these results, we now show the number of detectable genes in vegetative tissues including roots, leaves, endosperms and stems, and in reproductive tissues including ear, tassel, inflorescence, embryo and kernel in Fig. S5A-5D of the revised manuscript.*

***Regarding concern in Line 150 in last version:*** *We added more explanation and support for the details in the new manuscript. We have changed the legend of Fig. 1D to “Bar plot, number of genes specifically transcribed (yellow), specifically translated (green), or both transcribed and translated (light green) in a single tissue type across different tissue categories; the circular diagram refers to the mean value of specifically expressed genes (SEGs) after dividing by the sample number in each tissue category.” (Lines 179-182 in the revised manuscript). The SEG numbers are shown in the bar plot of Fig. 1D. The detailed information for the mean numbers of SEGs in each category is summarized in Fig. S5E-5F.*

Fig. S5 Detected genes and SEGs in different tissue types. **A**-**D**, Number of detected genes in leaves and endosperm (vegetative tissues), roots and stems (vegetative tissues) and reproductive tissues including ear, tassel, inflorescence, embryo and kernel. **E**, Number of SEGs and samples in different categories. **F**, Mean number of SEGs in each category after dividing by the sample size.

***Regarding the question about line 360 and Figure 6B in last version:*** *Some target genes showed different expression pattern, which may be associated with the function of GENIE3 that can detect non-linear relationships and both positive and negative relationships (Huang et al, BMC Plant Biol, 2018, PMID: 29879919; Huynh-Thu et al, Plos ONE, 2010, PMID: 20927193). We added this interpretation on lines 414-419 in the new manuscript. In addition, some target genes, such as D3 and SAUR39, showed different expression changes (upregulation) in the grf6 mutant. The added interpretation is as follows:*

*“Multiple targets predicted by GRN showed transcript patterns similar to the translation pattern of ZmGRF6, except the genes Dwarf plant 3 (D3), Gibberellin 2-oxidase6 (ZmGA2ox6), Abscisic acid 8’-hydroxylase1 (ABH1) and Small auxin up RNA39 (SAUR39), which might be related to the function of GENIE3 that can reveal non-linear relationships and both positive and negative linear relationships (Fig. 6B) [13].”*

*The 101 functional genes were all classic genes that had been confirmed to be associated with kernel development. We added a reference (article list) for these genes in Sheet 2 of Supplemental Table S4.*

4. In general, for a computational heavy study like this, the description of data analysis can be improved with providing more details. The manuscript needs language editing for clarity and accuracy, especially for such a complex data analysis. A data analysis flow chart might help too.

*Reply:* *Thanks for your helpful suggestions on the description/written. We've made a more comprehensive language editing and polish to improve the clarity and accuracy by a native English Scholar. We also added a flow chart of the data analyses according to your suggestion (Fig. S1).*

Fig. S1 The flow chart of this study.

5. In the result text, it is not explained how GRN are constructed. In line 200, it is not clear why the four datasets are used, other than merging the means of the rep1 and rep2 with the bulk data to form a bigger panel of input data. This also led to problems later in the manuscript that it is unclear which GRN (from which dataset) was used (for example line 282).

*Reply:* *Thank you for these helpful comments and suggestions.*

*We collected information on all maize TFs from PlantTFDB (http://planttfdb.gao-lab.org/tf.php?sp=Ppe&did=Prupe.I004500.1.p). We set 1,764, 1,788, 1,824, and 1,787 TFs detected at both the translatome and transcriptome levels as “regulators” in groups rep1, rep2, mean, and bulked, respectively (Fig. 2A). We also set 25,546, 25,714, 26,158, and 25,291 detected genes as potential “targets” across different datasets (Fig. 2A). We constructed putative GRNs based upon the expression patterns of TF genes and their target genes using the classic software GENIE3 with the random forest algorithm in rep1, rep2, mean, and bulked datasets. The mmGRNs were generated by combining the transcript levels of TF genes and target genes; the TTGRNs represented the combination of TF and target translation levels, while the TmGRNs were obtained by combining TF translation levels and target transcript levels. We describe how we generated the GRNs in results and Methods (Lines 204-217, and Lines 672-679).*

*We considered to merge the means of the rep1 and rep2 with the bulk data before, however, the batch effect between the bulk data and two reps might introduce more errors and offset the advantages of bigger panel of the input data. Furthermore, the predicted regulatory relationships are usually non-saturated based on a single dataset. In the original manuscript, we had used* *four datasets (transcriptome and corresponding translatome in rep1, rep2, mean and bulked) to generate multiple GRNs. Merging these GRNs allowed us to increase the prediction of regulatory relationships previously missed by the single dataset, although some false positives were likely also introduced. We hoped to obtain more useful information from the enlarged network and provide more helpful information for the maize community. Moreover, we looked at the overlap between these GRNs to further improve prediction accuracy. We also discuss how to reduce the false positive rate on lines 570-583 in the revised manuscript.*

*The integrated multi-omics GRNs (Union GRN) were used to further analysis. The multi-omics GRNs are the union of all inter-GRNs and intra-GRNs from the four datasets. To clarify this, we summarized the related information in the flow chart (Fig. S1) in the newly revised manuscript.*

6. In Figure 3B, how the results for 86 TFs are synthesized to form the plot?

*Reply:* *A regulatory network of 104 transcription factors of maize had previously been constructed (Tu et al, Nat Commun, 2020, PMID: 33037196). These 86 TFs were analyzed in both the previous study and our study. The regulatory network of these 86 TFs (from ChIP-seq data) was compared to the predictions of each GRN. We also generated a control with randomly selected TF-target pairs using the same TFs set and targets set for each dataset. The random selection of TF-target pairs was performed 1,000 times for each GRN in each dataset. The average value of the overlaps between random targets and ChIP-seq targets was compared to the overlaps between GRN predictions and ChIP-seq targets using the χ2 test.* *In addition, because mmGRNs and TmGRNs were constructed using the same TFs set and targets set in each dataset, the comparison of overlapping targets in mmGRN and TmGRN was also performed using the χ2 test (lines 274-280 in new manuscript).*

7. In Figure 3F, TP and FP refers to the motifs detected, or whether the target gene promoters have the motifs or not?

*Reply:* *Thank you for the comment. TP and FP refer to the percentage of promoters matching the motif (Table S3).*

*TP: Number of primary sequences matching the motif / number of primary sequences (percentage of primary sequences matching the motif); primary sequences are from the predicted targets.*

*FP: Number of control sequences matching the motif / number of control sequences (percentage of control sequences matching the motif); control sequences are from the genes that randomly selected in the set of expressed genes.*

*We deposited this information into the legend of Figure 3F (Lines 285-289 in new manuscript).*

8. In line 221, it was mentioned that union networks and intersected (overlapped) high confidence networks were created, how are these two networks used later?

*Reply:* *The Union network were mainly used later, we clarified this point in our revised manuscript in Fig.S1. The Union network was used to the confirmation of GRNs and the construction of the potential maize functional landscape of Fig. 4 and Fig. 5. The Union GRN also provided a guidance for regulatory relationships in the functional analysis of single genes. The high-confidence networks provided reliable target predictions for the functional analysis of specific genes: for example, ARFTF30 (Zm00001d026590) and Auxin-like 1 protein (Zm00001d039120), which were validated by RNA-seq (Fig. 6D), are the predicted targets of GRF6 in high-confidence networks (In Additional File 2).*

9. FigS3 A B and C: please explain in detail what was being used in the correlation.

*Reply: Fig. S3 from the original manuscript is now Fig. S6 in the revised manuscript.*

*Fig. S6A shows the correlations between translatome and transcriptome across all tissues:* *Comparisons between translatome and transcriptome as performed in every two tissues using the Pearson correlation coefficient.*

*Fig. S6B shows the correlation between the median translation level and transcript level of genes****:*** *Median value of translation level for each gene across all tissues, and* *median value of transcript level for each gene across all tissues. All median values at the translatome level were compared to the median values at the transcriptome level using Pearson’s correlation coefficients.*

*Fig. S6C Distribution of correlations between translation level and transcript level for genes across all tissues****:*** *Translation and transcription levels of each gene were* *extracted from every tissue. The comparison was performed between translation and transcript levels for each gene in the same tissues set using Pearson’s correlation coefficients. All correlation coefficients were plotted as a density plot.*

*In the revised manuscript, we added more details in the figure legend of Fig. S6 in Additional file 1.*

10. One main point made in the manuscript is that the tmGRNs built with translatome data is better than the intraGRNs, and this needs to be better supported. It seems that the top 1 million edges from each GENIE3 output was used. Is the threshold edge weight/confidence comparable between different types of GRNs? It seems that the tmGRNs have better weight among the top 1 million edges, suggesting that the tmGRNs are higher confidence GRNs than other GRNs anyway. Therefore, it is less surprising that it has bigger number of overlap with the ChIP-seq results. If the same edge confidence threshold was used to trim different GRNs, will tmGRNs still have better overlap with the TF ChIP-seq results than the other GRNs (after normalized to GRN sizes)?

*Reply:* *Thanks for your helpful comments and suggestions.*

*Regarding the question “Is the threshold edge weight/confidence comparable between different types of GRNs?”****:*** *We think that you are referring to Fig. 3G. We agree that the edge weights of top 1 million in different GRNs are not comparable. We should have clarified this point for Fig. 3G in the previous version of the manuscript. Fig. 3G does not show edge weights of the top 1 million but rather the overlapping relationships from the three different GRNs shown in Fig 3A and Fig. S8. The edge weights are from the overlapping GRN predictions (high-confidence), therefore, the edge weights for predictions that exist in all three types of GRNs are comparable. The TmGRNs had significantly higher weights than mmGRNs at each edge level. We modified this point as “In addition, we compared weights of overlapping GRN predictions (overlaps of the three types of GRNs) in mmGRNs and TmGRNs across different thresholds (top 0.2 million edges, 0.6 million edges, and 1 million edges) and observed that TmGRNs have significantly higher weights than mmGRNs at each edge level (Fig. 3G).” (Lines 305-309 in the revised manuscript).*

*Regarding the question “If the same edge confidence threshold was used to trim different GRNs, will tmGRNs still have better overlap with the TF ChIP-seq results than the other GRNs (after normalized to GRN sizes)?”****:***

*In the dataset rep1, three types of GRNs (mmGRN, TmGRN and TTGRN) were constructed using the same regulator set (1,764 TFs) and target set (25,546 genes) (Fig. 2A). The same TFs list and same targets list with the same order were used to construct the three types of GRNs. Therefore, the size of each type of GRN is the same or very close. The different transcript/translation combinations of ‘TF vs. target’ (translation vs. translation, transcription vs. transcription, translation vs. transcription) determined the prediction of regulatory relationships between TFs and targets. The ‘top one million edges’ can represent the same* *ratio of GRN predictions under the same GRN size. The ‘TFs vs. targets’ in rep2, mean and bulked were ‘1788 vs. 25714’, ‘1824 vs. 26158’ and ‘1787 vs. 25291’, respectively (Fig. 2A). We performed the same analyses consistently to construct the three types of GRNs using these datasets.*

*In order to better assess whether tmGRNs have better overlap with the TF ChIP-seq results than other GRNs, we used the same edge weights to trim different GRNs. The ChIP-seq target numbers of 86 TFs mainly ranged from 2,000 to 3,000. When the edge weight was≥0.0003, the size of each type of GRN was close to the maximum number of 5 million, which detected more than 2,000 GRN targets for each TF (about 1,800 TFs in total in each dataset). The GRN targets number approximates to the ChIP-seq target number for single TF. We compared each type of GRN to the ChIP-seq of 86 TFs and found that the overlapping targets in TmGRNs are significantly more than in the mmGRNs and TTGRNs (**Fig. S9A and 9B). After normalization by GRN size and ChIP-seq target number, the probability of overlap for TmGRNs was still higher than that for mmGRNs and TTGRNs with ChIP-seq data (Fig. S9C and 9D).* *Overall, TmGRNs have better performance than mmGRNs and TTGRNs.*

Fig. S9 TmGRNs have more overlapping targets with ChIP-seq (weight ≥ 0.0003). **A.** The Number of overlapping targets for the three types of GRN with ChIP-seq for the comparison of targets from 86 TFs; the χ2 test was performed for the comparison of the three types of GRN with ChIP-seq; **B**. Number of overlapping targets between the three types of GRN with ChIP-seq data for each TF gene (paired Student’s *t*-test). **C and D.** Probability of overlaps between GRN and ChIP-seq for each TF gene after normalization by GRN size and the number of ChIP-seq targets (paired Student’s *t*-test). “*” represents *P* < 0.05, “***” represents *P* < 0.001.

*To further consolidate this conclusion, we further raised the threshold to edge weight to≥0.01. We obtained similar results: that is, the overlaps for TmGRNs with ChIP-seq were more than those for mmGRNs and TTGRNs (Fig. S10A-10D). These results are reflected in lines 253-257 and lines 261-263 in the revised manuscript.*

Fig. S10 TmGRNs have more overlapping targets with ChIP-seq (weight≥0.01). **A.** Number of overlapping targets for the three types of GRN with ChIP-seq for the comparison of targets of 86 TF genes; the χ2 test was performed in the comparison of the three types of GRN with ChIP-seq; **B**. Number of overlapping targets between the three types of GRN with ChIP-seq data for each TF gene (paired Student’s *t*-test); **C and D.** Probability of overlaps between GRN and ChIP-seq for each TF gene after normalization by GRN size and the number of ChIP-seq targets (paired Student’s *t*-test). “*” represents *P* < 0.05, “***” represents *P* < 0.001.

11. In the current manuscript, is the difference in overlaps with ChIP-seq between TmGRNs and mmGRNs significant (lines 241-246, Figure 3B&3C)? The only significant difference between tmGRNs and mmGRNs of this part is shown for Fig 3D, limited to the four TFs, but how are these four TFs selected?

*Reply:* *In Figure 3B, the difference in overlap with ChIP-seq between TmGRNs and mmGRNs is significant (χ2 test, rep1: P < 0.001, rep2: P < 0.001, mean: P < 0.001, bulked: P < 0.007). We added the significance information into Fig. 3B.*

*In Fig. 3C, the difference in overlap with ChIP-seq between TmGRNs and mmGRNs was significant overall (violin plot) (paired t-test, rep1: P < 0.00021, rep2: P < 0.0005, mean: P < 0.0009, bulked: P < 0.101). We added the significance information into Fig. 3C and Fig. S11. Most TFs out of the 86 TFs had more overlaps with ChIP-seq for TmGRNs than for mmGRNs.*

*In Fig. 3D, 3E, and Fig. S7, the four TFs are classic genes with high-quality ChIP-seq data. The regulatome of FEA4 and KN1 had been parsed thoroughly in multiple previous studies. The ChIP-seq experiments of KN1, O11 and ZmABI19 were performed using the KN1-specific antibody (Bolduc et al, Genes Dev, 2012, PMID: 22855831), O11-specific antibody (Feng et al, The Plant Cell, 2018, PMID: 29436476) and ZmABI19-specific antibody (Yang et al, The Plant Cell, 2021, PMID: 33751093) in native maize plants, respectively. The targets of these TFs are more accurate, which is why we chose these TFs for the comparison.*

12. In the last few sections validating the GRNs (with photosynthesis genes, MYBs and GRF), is the tmGRN outperforming the other GRNs?

*Reply: The TmGRNs did outperform mmGRNs in most datasets for photosynthesis genes, except for the bulked GRNs (Fig. S15A). However, the overlapping targets for TmGRNs were more than those for mmGRNs overall, after merging the same type of GRNs in the four datasets (Fig. S15B). (Lines 336-338 in the revised manuscript).*

Fig. S15 Comparison between mmGRNs and TmGRNs in overlapping targets with ChIP-seq.

**A.** Overlap number between mmGRN and ChIP-seq targets, TmGRN and ChIP-seq targets across rep1, rep2, the mean of two replicates, and the bulked datasets; **B.** Overlap number between GRN and ChIP-seq targets when merging each type of GRN in rep1, rep2, the mean, and the bulked.

*In the section on GRF6, TmGRNs outperformed mmGRNs in all four datasets (Fig. S19A). Moreover, the overlapping targets for TmGRNs were more than those for mmGRNs overall, after merging the same type of GRNs in the four datasets (Fig. S19B). (Lines 426-428 in the revised manuscript)*

Fig. S19 Comparison between mmGRNs and TmGRNs in overlapping targets with DEGs.

A. Number of overlapping targets between mmGRN and DEGs, TmGRN and DEGs in rep1, rep2, the mean, and the bulked datasets; B. Number of overlapping targets between GRN and DEGs when merging each type of GRNs in rep1, rep2, the mean, and the bulked datasets.

*In the section on MYBs, we did not perform this comparison because the expression of MYB31 was only detected by transcriptome, but not the translatome, due to the low translation level of MYB31 or the technical limitation of Ribo-seq.*

13. To validate the GRN prediction, leaf TFs and photosynthetic genes were focused on. In Figure 4B, is this overlap significant considering the background is limited to the interactions between photosynthesis genes and leaf TFs? How are the four TFs selected (line 298)? LHCB7 and PSB29 are already shown to be the direct targets of TFs by previous ChIP-seq, therefore, the luciferase assay validating the gene activation is less impressive in proving the predicting power of the GRNs. To validate the predicting power of the GRNs, it is better to pick the genes from the 241 gene list in Figure 4B to show that GRNs can identify new regulatory relationship between TFs and photosynthesis target genes.

*Reply: Thank you for your helpful comments.*

*In Figure 4B, the overlap was significant. We generated the random control with the same number of pairs, using the same leaf TFs and photosynthesis genes (1,000 randomizations). The average values of the overlaps between random control and ChIP-seq regulatory pairs were used to perform a χ2 test (combining the overlaps between GRN predictions and ChIP-seq regulatory pairs). The P-value was <0.0007, supporting significance. We added these details on lines 334 -335 in the revised manuscript.*

*The four TFs were selected because they could target more photosynthetic genes. Although LHCB7 and PSB29 are known to be the direct targets of COL8 and GLK1 based on previous ChIP-seq, the luciferase assay confirmed these relationships and suggested the positive role of the two TFs in regulating the expression of LHCB7 and PSB29.*

*In addition, we selected another two key TFs (BHLH145 and HB38) that can target more genes and two key targets (**Zm00001d020002 [ortholog of AtLHCB7] and* *Zm00001d024372 [ortholog of AtLHCA1]) that were targeted by more TFs from the 241 regulatory pairs predicted only by GRN (Fig. S16A), to perform a transient luciferase assay to verify their regulatory relationships. We determined that* *BHLH145 can positively regulate the transcription of Zm00001d020002 and Zm00001d024372, while HB38 could positively regulate the expression of Zm00001d020002 (Fig. S16B and 16C). This result demonstrates that GRNs can identify new regulatory relationship between TFs and photosynthesis target genes. We added this into the revised manuscript on lines 347-355.*

Fig. S16 The 241 regulatory pairs (predicted only by GRN) between photosynthesis genes and leaf TFs. **A.** The 241 regulatory pairs predicted only by GRN; **B.** Transient luciferase assay between BHLH145 (TF) and Zm00001d024372 (target); **C.** Transient luciferase assays between BHLH145 (TF) and Zm00001d020002 (target), and between HB38 (TF) and Zm00001d020002 (target).

14. To validate the GRN prediction and to show that GRNs could be used to drive new discovery, GRF and MYB TFs were focused on. How are the specific GRF and MYB selected? Figure 6A and 7A: are these all the targets predicted by GRNs? Which GRNs (Tm, mm, and rep1, rep2, etc) was used? Line 366 on GRFs: is the overlap overall significant thus proving the power of the GRNs in predicting target genes? Similarly, for MYB, do the regulated targets of MYB31 and the bound targets detected by cut-and-tag overlap significantly with the GRN targets? Besides, it should be mentioned that the bound targets were performed in protoplasts not in whole plants.

*Reply:* *Thank you for your helpful comments.*

*GRF family plays important roles in plant growth and development, while the MYB family (one of the largest TF families) functions in multiple biological process including development, cell division, cell wall, secondary metabolism and abiotic stimulus. The two TF families are hot topics of research. We predicted the targets for GRFs and MYBs using our constructed GRNs. We found the ZmGRF6 might be a key TF that can target multiple other GRF TF genes and could itself be targeted by multiple other GRF TFs (Comments Figure 1A). Our lab had also previously isolated a mutant in ZmGRF6 that was generated by CRISPR/Cas9-mediated editing, which is one reason we selected GRF6 for follow-up studies* *here. We exemplified the case of ZmGRF6 for the utilization of the GRNs. Additionally, we performed a GO enrichment analysis for the targets of MYB31 and found that the expected function as similar to the known functions of this family (MYBs). ZmMYB31 was shown to have important roles in development, cell wall and secondary metabolism (Fornale´ et al, Plant J. 2010, PMID: 21070416). Here, we found that this TF also likely play a role in abiotic stimulus in addition to development, cell wall and secondary metabolism (Comments Figure1B). We also obtained one mutant (introducing a premature stop codon in the coding sequence) of* *ZmMYB31. We hope to uncover new functions for this known TF under the guidance of GRNs.*

Comments Figure1. Predicted regulatory relationships between *ZmGRF6* and its homologous genes (**A**), and functional prediction for *ZmMYB31* based on its GRN targets (**B**).

*Using the same set of TFs and targets to randomly generate the TF-target pairs that have the same rows compared with the Union GRN, we extracted the targets of GRF6 in the random TF-target pairs and overlapped them with the DEGs. We repeated these analyses 1000 times and collected the average values as the random control. The probability of overlaps between DEGs (regulated targets) and* *our GRN targets for GRF6 was significantly higher than that of the random control (χ2 test, P < 0.0024). We added this on lines 425-426 in the revised manuscript.*

*The random control for MYB31 was generated using a similar method. The overlaps between DEGs (regulated targets) and GRN targets of MYB31 showed significant differences compared to the random control (χ2 test, P < 0.0194). The probability of overlaps between the bound targets detected by CUT&tag and GRN targets of MYB31 was higher than that of the overlap between random control and CUT&tag targets (χ2 test, P < 0.05). The CUT&tag performed in protoplasts might induce some bias in detecting the bound targets of ZmMYB31, which is likely the reason why the P-value only approximates to significance (0.05).*

15. Figure 8A and 8C: what is the significance level of the difference between WT and mutant?

*Reply: Thank you for pointing out this important result was missing. The significance level of PAL7 was 0.004 (P-value). The significance level of ZmC4H was <0.074 (P-value), which implies a trend toward significance. We have added the significance and P-values to Fig. 8A and 8C.*

16. Minor points:
Overall texts are too small to read in figure.

*Reply: We apologize. We have increased font size for most of the text in all figures in the revised manuscript.*

Line 166: please briefly explain the meaning of TE for audience who is not very familiar with the term.

*Reply:* *Excellent advice. Translational efficiency (TE), an important index for translation, was calculated by FPKM_Ribo-seq_ /FPKM_RNA-seq_ and used to measure the efficiency of RNA utilization (Lei et al, Plant J. 2010, PMID: 26568274). We have added a brief explanation of TE to the figure legend of Fig. S6D in the revised manuscript.*

The part on GRFs (line 331-342) goes better with the next result section.

*Reply: Thank you for this suggestion. Now done (Lines 389 to 400 in the revised manuscript).*

**Reviewer #2:** Building gene regulatory networks with high accuracy will be very useful to the plant research community. In this ms, the authors collected the translatome and transcriptome datasets and constructed a set of multi-omics GRN, and tested its reliability with additional experiments. Overall, the ms is very interesting, and I have only several scientific comments.

*Reply:* *We appreciate your careful reading, evaluation, and positive comments on our manuscript. We have revised the manuscript according to your suggestions. Please see our point-by-point answers below.*

1. The authors concluded that" inter-omics GRNs outperform intra-omics GRNs". And seems to emphasize that TmGRNs outplayed the mmGRNs in the following ms. I am wondering how TTGRN compared with TmGRN. From Figure 2D, the networks constructed from overlapping edges among mmGRNs, TmGRNs, and TTGRNs provided a better GRN, why the authors did not focus on this high-confidence network?
In line 275, "These results indicate that inter-omics GRNs outperform traditional intra-omics GRNs for demonstration of the regulatome in maize." I would suggest the authors take a look at the TTGRNs and give an explanation if it is not as good as TmGRNs.

*Reply:* *Thank you for your helpful comments and suggestions.*

*As requested, we performed additional comparisons among mmGRNs, TTGRNs and TmGRNs, shown in Fig. S9 and Fig. S10. Considering that the number of ChIP-seq targets for the 86 TFs mainly ranged between 2000 and 3000, there might be 5 million regulatory relationships for all ~1,800 maize TFs (keeping more than 2000 GRN targets that approximated to the ChIP-seq target number for each TF). With GRN edge weight≥0.0003, the size of the GRNs was close to this number of 5 million, which is the theoretical number of predicted targets for the 1,800 maize TFs. We compared each type of GRNs to the ChIP-seq data from 86 TFs, and found that the numbers of overlapping targets of TmGRNs were significantly higher than those of mmGRNs and TTGRNs (Fig. S9 A and 9B). After normalization by GRN size and ChIP-seq target number, the overlaps in TmGRNs were still greater than those from mmGRNs and TTGRNs (Fig. S9C and 9D). Together, these results suggested that TmGRNs not only showed better performance than mmGRNs, but also showed better performance than TTGRNs.*

Fig. S9 TmGRNs have more overlapping targets with ChIP-seq (weight ≥ 0.0003). **A.** Number of overlapping targets for the three types of GRN with ChIP-seq for the comparison of targets from 86 TFs; the χ2 test was performed in the comparison of the three types of GRN with ChIP-seq; **B**. Number of overlapping targets between the three types of GRN with ChIP-seq data for each TF (paired Student’s *t*-test); **C and D.** Probability of overlaps between GRN and ChIP-seq for each TF after normalization by GRN size and number of ChIP-seq targets (paired Student’s *t*-test). “*” represents *P* < 0.05, “***” represents *P* < 0.001.

*To consolidate this conclusion, we further raised the threshold to edge weight≥0.01. We obtained similar results that the overlaps for TmGRNs with ChIP-seq are more than that for mmGRNs and TTGRNs (Fig. S10A-10D). These results are reflected in lines 253-257 and lines 261-263 in the revised manuscript.*

Fig.S10 TmGRNs have more overlapping targets with ChIP-seq (weight≥0.01). **A.** Number of overlapping targets for the three types of GRN with ChIP-seq for the comparison of targets from 86 TFs; a χ2 test was performed in the comparison of the three types of GRN with ChIP-seq. **B**. Number of overlapping targets between the three types of GRN with ChIP-seq data for each TF (paired Student’s *t*-test). **C and D.** Probability of overlaps between GRN and ChIP-seq for each TF after normalization by GRN size and number of ChIP-seq targets (paired Student’s *t*-test). “*” represents *P* < 0.05, “***” represents *P* < 0.001.

Figure 2D illustrates a high-confidence GRN network, which was generated by the overlap between the three types of GRNs. Most TFs had far fewer predicted targets (Comments Figure 2), which is significantly biased from the theoretical number deduced from 102 TF ChIP-seq data (*Tu et al, Nat Commun, 2020*). Such a high-confidence GRN was not comprehensive or not sufficient to be compared to the GRNs in this study, or for GRN verification and TF functional confirmation/exploration. It is also limited or not enough to profile the potential functional landscape of maize. However, the high-confidence GRN will be a useful resource for exploring the function of individual genes in Functional Genomics.

Comments Figure 2. Distribution of edge number for each TF in the high-confidence network.

2. Line 200-202, how did the authors identify the 1,764, 201 1,788, 1,824, and 1,787 TFs at both translatome and transcriptome levels as "regulators"? it is better to give a short description in the text.

*Reply:* *Thank you for giving us the opportunity to make this point clearer.* *We collected information on all maize TFs from PlantTFDB (Jin et al, Nucleic Acids Research, 2017). We then looked for evidence of transcripts in our RNA-seq and Ribo-seq datasets for all TF genes, and selected those expressed TFs, yielding the reported numbers of 1,764, 1,788, 1,824, and 1,787 TFs at both the translatome and transcriptome levels in rep1, rep2, mean and bulked, respectively. We have added a detailed description on lines 204-206 in the revised manuscript.*

3. Line 237-240, "We found that our different GRNs showed significantly more overlap with ChIP-seq data than a control GRN (randomly generated network with the same TFs and targets) (Figure 3B)." It is better to explain what is the "randomly generated network" and what kind of "comprehensive comparison" were done between GRNs and ChIP-seq results.

*Reply:* *Indeed, thank you for pointing this out.*

*A regulatory network of 104 transcription factors of maize had previously been constructed in a previous study (Tu et al, Nat Commun, 2020). There were 86 TFs (in the 104 TFs) were also detected in our study. The regulatory network of these 86 TF genes (ChIP-seq data) was overlapped with the predictions of different GRNs in our study.* *The control consisting of TF-target pairs was generated randomly using the same TF sets and target sets for each dataset. The random TF-target pairs were generated 1,000 times for each GRN in each dataset. The average value of the overlaps between random targets and ChIP-seq targets was compared to the overlaps between GRN predictions and ChIP-seq targets using the χ2 test. We added the explanation to the figure legend of Fig. 3B (Lines 274-278).*

*Comparisons between GRNs and ChIP-seq results were centered on the overlapping targets for the overall 86 TFs with ChIP-seq data from protoplasts and four well-known classical TFs with published ChIP-seq data. We used three types of edge thresholds (top 1 million, edge weight ≥ 0.0003 and edge weight ≥ 0.01) and generated GRNs of different sizes to compare to the ChIP-seq targets of the 86 TFs. Moreover, we also analyzed the matching rate of binding motifs of 11 TFs for the promoter region of the top100 predicted targets (Figure 3F and Table S3).*

4. Line 279-283, the authors described using 11 well-known kernel-related TFs to extract the kernel-related GRN sub-networks. How many tissues/stages-specific edges/sub-networks did the authors identify? I suggest the authors provide a link to access these GRNs and all the GRNs that have been built in this ms, especially the edges overlapped among the three GRNs. In this way, this dataset could greatly help the whole maize community.

*Reply:* *Thank you for your valuable comments and suggestions.*

*We identified four tissue-specific sub-networks based on the specific expression of genes (Fig. S18). We found that some genes showed specific translation in leaves, roots, the SAM/embryos and endosperm. The TFs and targets were extracted from these gene sets in different tissues, and their regulatory relationships were also extracted from the Union GRN. Multiple sub-GRNs were generated and the predicted regulatory pairs are more likely to have occurred in these tissues, respectively. These sub-GRNs are available in Additional Files 4-7. (Lines 374-381 in the revised manuscript).*

Fig. S18 Tissue/stage-specific translated genes in leaves, roots, the SAM/embryos and endosperm.

*We have deposited the Union GRN, High-confidence-GRN and specific sub-GRNs to our lab website (*[*http://zeasystemsbio.hzau.edu.cn/dataset.html*](http://zeasystemsbio.hzau.edu.cn/dataset.html)*), which is freely accessible for the research community.*

5. Line 294, what's the proportion of these 23 LHC-like genes in all LHC-like genes in maize?

*Reply:* *Thanks for your valuable comments. We performed an annotation using reference genome sequences (V4) of B73 in PlabiPD (https://www.plabipd.de/portal/web/guest/mercator-sequence-annotation) with Mercator v.3.6 (Mapman). We found 35 LHC or LHC-like genes through the mapman annotation. The 23 LHC-like genes accounted about 65.7% in these identified LHC related genes. The other genes either showed no expression in our collected tissues or were not detected in the ChIP-seq data of the 29 leaf-specific-TFs.*

.

**Second round of review**

**Reviewer 1**

The manuscript is much improved. Thank you for responding to the comments. I still have some concerns including what has been previously stated.

1) The lack of replicates and statistical power is still a concern for me. The author compared to Walley et al 2016, but that dataset has three biological replicates for 23 tissues for RNAseq and more biological replicates for 33 tissues for proteomics. What we have here for each GENIE3 is 20 tissues type without replicate. The input data, due to uneven replicates and batch, is separated into a few parts thus undermining the power of big data analysis.

2) For singe TF follow up RNA-seq, two replicates are limited but okay since cuffdiff seems to perform well enough with two replicates. Semi quantitative PCR as validation is outdated, and still no replicates. See similar journal like Plant cell outlined the expectations for replicates and quantitative measurement of RNA (https://academic.oup.com/plcell/pages/General-Instructions#Data_Availability)

3) About the four different data sources: please clarify the set of “mean” - “in silico data derived from calculation of mean values of the two separately sequenced replicates”, does it mean the “mean” set is the mean of rep1 and rep2, or the mean of rep1+rep2 and the bulked for the overlapped tissues type?

4) Please clarify how the union and overlap network of Figure 2 C & D are created. It is stated that “We also used the overlap among the top one million edges of the mmGRNs, TTGRNs, and TmGRNs from each dataset to create a network comprising 189,271 high-confidence regulatory pairs”. Was the union (or merge) occurring at both network type levels (TT, TM, MM) and at data input levels (rep1, rep2, mean and bulked)? How about the overlap network? Was the overlap happening at network type level first (TT, TM, MM), and then union (or intersect) among data sources? The author should be aware that since the data sources are not completely independent (the “mean” set has overlap with the other sets), if the summary network is generated by intersection between different GRN generated from different data sources, the process is biased.

5) To compare inter vs intra-omics: Only gene number without considering the background of the overlap is not a good indication of the significance of overlap (Figure 3). The data actually looks like ttGRN performs best among three. Based on the AUPR and AUROC in Figure S13 (btw, the comment table 1 was mis-labeled, there are two mmGRNs), for half of the cases (rep 2, bulked), ttGRN performs best, in one case (rep 1) ttRNA is rather comparable to Tm GRN, only in “mean” set, TmGRN performs better to ttGRN and mmGRN. Therefore, it is hard to support that tmGRN performs better than intra networks. In many places the tmGRN and mmGRN were compared, but the ttGRNs were ignored. Another logic problem is that if the author argues that tm network is the best, why is the summery GRN (union or intersection of tt, tm and mm GRNs) are used for the later validations. In later validations, different GRNs were used for different TF/steps, please explain why. For example, for the kernel network, it was first stated that union GRN is used (across network types, line 316) but later it was said that tmGRNs was used (line 319). Sometimes, the overlap of the three types of GRNs was used (line 367).

6) Please explain how the TFs are picked in the manuscripts, this include the “86 TFs” (in author response it is explained. Please include it also in the manuscript), the 11 TFs (line 297), and the 11 TFs related to kernel (line 319).

7) Overall, I would like to see the explanation provided in response to reviewer to be included in the main manuscript to be clear for the audience. For example, the reference about the 101 functional genes in kernel.

8) As a community resource, how does this network compare to previously published maize networks from big data analysis (like huang et al plant physiology and Walley et al science)?

Minor: Figure 2A: label the columns better

Line 372 “suggesting that target prediction using GRNs is highly accurate. “ this is an overstatement based on functional enrichment.

**Reviewer 2**

The revised ms addressed most of my questions. I only have several minor points want to discuss.

1. In lines 564 and 567, the authors mentioned pmTGN. However, this term has not been introduced in the results part. Please check.

2. Since TFs from translatome regulate the transcription of genes, it is reasonable that TmGRN outperforms mmGRN in recapitulating ChIP-seq networks. Does TTGRN also outperform mmGRN?

**Authors Response**

**Point-by-point responses to the reviewers’ comments:**

**Reviewer #1:**

The manuscript is much improved. Thank you for responding to the comments. I still have some concerns including what has been previously stated.

*Reply:* *Thank you very much for your careful reading, evaluation, and the valuable comments on our manuscript. We have revised the manuscript according to your suggestions. Please see our point-by-point answers below.*

1) The lack of replicates and statistical power is still a concern for me. The author compared to Walley et al 2016, but that dataset has three biological replicates for 23 tissues for RNAseq and more biological replicates for 33 tissues for proteomics. What we have here for each GENIE3 is 20 tissues type without replicate. The input data, due to uneven replicates and batch, is separated into a few parts thus undermining the power of big data analysis.

*Reply:* *We appreciate this comment. We should have described this more accurately in our previous manuscript. We did have replicates for the construction of GRN. Given that GENIES3 allows expression values from one replicate or mean value from different replicates, we hypothesized the union or intersection of GRNs from individual replicates would be more robust than that from mean values of different replicates. So, we analyzed our data from five replicates separately for the construction of GRN and then conducted the following analyses based on the union or intersection of GRNs.*

*To make it clear, we removed the constructed GRNs in rep1 group and rep2 group, these GRNs were constructed only based on single replicate (rep1 or rep2). The mean version from two biological replicates and the bulked one from three biological replicates were retained in the revised manuscript. The GRNs of mean version were constructed using the average expression values of genes in rep1 and rep2, while the GRNs of the bulked version were constructed using the expression values of genes produced by sequencing on the bulked samples from three independent biological replicates (Figure 2A). Each bulked sample was composed of equal amount of tissue sample from three biological replicates. Due to the high-cost of the Ribo-seq, the bulked samples were subject to Ribo-seq, which can reduce the random bias. Importantly, all the data were generated from our own lab at the same grown condition, which ensure the power comparison between TTGRN, TmGRN, and mmGRN in terms of the recapitulation of the ChIP-seq network for transcription factors. We believe that the comparison of GRNs from different omics layers is novel and interesting to the research community of maize and even the other wide range of species.*

**Figure 2A.** The mean expression values of two replicates and the expression values produced in bulked samples were used for GRN construction.

2) For singe TF follow up RNA-seq, two replicates are limited but okay since cuffdiff seems to perform well enough with two replicates. Semi quantitative PCR as validation is outdated, and still no replicates. See similar journal like Plant cell outlined the expectations for replicates and quantitative measurement of RNA (https://academic.oup.com/plcell/pages/General-Instructions#Data_Availability)

*Reply:* *Thank you very much for your pointing-out on this. According to your guide, we performed the* ***quantitative RT-PCR (qRT-PCR)*** *assays, which showed accordant results for the DEGs detected by RNA-seq (Figure S18C). The results were added into the Additional file1--- Figure S18C in the revised manuscript, the detailed information of qRT-PCR was added into line 785-791, and the primer sequences were listed in Table S10.*

**Figure S18C.** The quantitative RT-PCR (qRT-PCR) was performed to verify several focused targets/DEGs using the RNA of WT and MU.

3) About the four different data sources: please clarify the set of “mean” - “in silico data derived from calculation of mean values of the two separately sequenced replicates”, does it mean the “mean” set is the mean of rep1 and rep2, or the mean of rep1+rep2 and the bulked for the overlapped tissues type?

*Reply:* *Thank you for this helpful comment. The set of “mean” is the mean of rep1 and rep2. We clarified this in line 208-209 in the revised manuscript.*

4) Please clarify how the union and overlap network of Figure 2 C & D are created. It is stated that “We also used the overlap among the top one million edges of the mmGRNs, TTGRNs, and TmGRNs from each dataset to create a network comprising 189,271 high-confidence regulatory pairs”. Was the union (or merge) occurring at both network type levels (TT, TM, MM) and at data input levels (rep1, rep2, mean and bulked)? How about the overlap network? Was the overlap happening at network type level first (TT, TM, MM), and then union (or intersect) among data sources? The author should be aware that since the data sources are not completely independent (the “mean” set has overlap with the other sets), if the summary network is generated by intersection between different GRN generated from different data sources, the process is biased.

*Reply:* *Thank you for the helpful comments and suggestions. Yes, we should have clarified these datasets and different GRNs in our previous manuscript. To remove the potential bias, the GRNs constructed from the single replicate of either rep1 or rep2 have been deleted in our revised manuscript. The union network was created by merging all GRNs at both network type levels (TT, TM, MM) and at data input levels (mean and bulked). The high-confidence regulatory network was generated by overlapping GRNs at network type level firstly (TT, TM, MM) and then merging the overlapped GRNs from different data sources (mean and bulked). We added the detailed descriptions about this in line 199-202 of our revised manuscript. Since “mean” is the mean of rep1 and rep2 replicates and “bulked” is from the other three replicates, the dataset “mean” and “bulked” are independent. Therefore, the subsequential analyses had no biases.*

5) To compare inter vs intra-omics: Only gene number without considering the background of the overlap is not a good indication of the significance of overlap (Figure 3). The data actually looks like ttGRN performs best among three. Based on the AUPR and AUROC in Figure S13 (btw, the comment table 1 was mis-labeled, there are two mmGRNs), for half of the cases (rep 2, bulked), ttGRN performs best, in one case (rep 1) ttRNA is rather comparable to Tm GRN, only in “mean” set, TmGRN performs better to ttGRN and mmGRN. Therefore, it is hard to support that tmGRN performs better than intra networks. In many places the tmGRN and mmGRN were compared, but the ttGRNs were ignored. Another logic problem is that if the author argues that tm network is the best, why is the summery GRN (union or intersection of tt, tm and mm GRNs) are used for the later validations. In later validations, different GRNs were used for different TF/steps, please explain why. For example, for the kernel network, it was first stated that union GRN is used (across network types, line 316) but later it was said that tmGRNs was used (line 319). Sometimes, the overlap of the three types of GRNs was used (line 367).

*Reply:* *Thank you very much for the constructive comments and suggestions. In the revised manuscript, we only retained the GRNs of mean and bulked, and removed the GRNs constructed using the single replicate of either rep1 or rep2 according to your above helpful comments and suggestions.*

***For your concern about the factor of the background in the overlap comparison in Fig. 3****: We did more comprehensive analyses in our revised manuscript, regulatory networks in overall. We overall compared the overlaps between three types of GRNs (mmGRN, TTGRN and TmGRN) from two datasets (mean and bulked) and random sampling (control) with the ChIP-seq results from 86 TFs using the χ2 test (Figure 3B). The Figure 3C showed the comparisons of overlapped targets number of each TF among three types of GRNs. To eliminate the* *background effect, we considered both the predicted GRN size (using the threshold of top one million) and ChIP-seq target number of each TF and used the ratios of overlapped targets to do the comparisons in Figure 3D (considering the factor of the predicted GRN size) and 3E (considering the factor of ChIP-seq target number). Overall, TmGRNs and TTGRNs showed better performance than mmGRNs. Generally speaking, TmGRNs are best. Specifically, it is consistent for some classical TFs (Fea4 and Kn1) (Figure 3F and 3G)* *using Paired t-test across three different thresholds (top 100, top 200 and top 500) for each TF.*

**Figure 3A-3I.** **A**, Overlap of mmGRNs, TmGRNs, and TTGRNs in mean group for the top 1 million edges. **B**, The overlapped targets number in 3 types of GRN by comparing to ChIP-seq targtes of 86 TFs, the χ2 test was used in comparison of three types of GRN. **C,** The overlapped targets number for each TF in 3 types of GRN in the group mean and bulked (Student paired *t*-test). **D,** The overlaps between GRN and ChIP-seq for each TF after normalization by GRN size of each TF (Student paired *t*-test). **E**, The overlaps between GRN and ChIP-seq for each TF after normalization by ChIP-seq targets number (Student paired *t*-test). **F** and **G**, The targets comparison between GRNs and ChIP-seq for FEA4 and KN1. **H**, Simple Enrichment Analysis (SEA) for the 1-kb sequences upstream for the start codons of the top 100 targets of 11 TFs in three types of GRN. **I**, Comparison of GRN weights of three types of GRN at different thresholds, significant differences were determined using the paired Student's *t*-test. “*” represents P < 0.05; “**” represents P < 0.01; “***” represents P < 0.001.

***For your concern about AUPR, AUROC and outperformance of TmGRN in Figure S13 and Figure 3:*** *Thank you for pointing out on this, it was our writing typo about TmGRN. In the mean dataset, the ROC of TmGRN is higher than the TTGRN, while it is opposite in the bulked dataset. Both TmGRN and TTGRN are higher than the mmGRN in accordance with that of Figure 3B~Figure 3G. Additionally, we performed the comparison between GRN and* ChIP-*seq for targets, motif analysis (Figure 3H) and weight (Figure 3I), we found that TmGRN and TTGRN outperform the mmGRN, the TmGRN also showed better performance than the TTGRN in most cases.*

***For the ignorance of TTGRNs in many places:*** *We added the TTGRN in Figure 3, Fig S9, Fig S10, Fig S11, Fig S12, Fig S14 and Fig S17 and accordingly added more description about TTGRNs in our revised manuscript.*

***For why is the summery GRN (union or intersection of tt, tm and mm GRNs) are used for the later validations:*** *Although the TmGRN showed better performance than other GRNs in many cases, there are still much useful information in mmGRN and TTGRN. Moreover, it is also limited or not enough to profile the potential functional landscape of maize only based on single omics layer. It may be more comprehensive or more accurate when consider three types of GRN for functional dissection of TFs. However, it’s novel and important to discriminate the power of different omics GRNs for the recapitulation the real regulatory targets (ChIP-seq or CUT&Tag) as described in our study.*

***In the revised manuscript, we only used the union networks in the subsequential analyses.*** *For the kernel network, we always used the Union network in fact, we are sorry that there was a writing mistake in the line 319 of our previous manuscript. For line 367, we have changed to use the Union network to detect the target genes in overlap of three types of GRNs to perform GO analysis (Line 394-404 in new manuscript). All the results showed consistent.*

6) Please explain how the TFs are picked in the manuscripts, this include the “86 TFs” (in author response it is explained. Please include it also in the manuscript), the 11 TFs (line 297), and the 11 TFs related to kernel (line 319).

*Reply:* *Thank you for your suggestions.*

*We added* ***the description of why chose these 86 TFs*** *in line 257-258 of our revised manuscript.*

***The 11 TFs (line 297)*** *were picked from different gene family, the TF from different gene family has more divergent binding motif on the target genes. Moreover, their binding motifs are solid or well-known in maize. The information of these TFs was added into line 323-325 of our revised manuscript.*

***The 11 kernel-related TFs (line 319)*** *are well-known and very famous genes, their roles and regulatory relationships were clear because many experiments (ChIP-seq, RNA-seq, qRT-PCR, Y1H, EMSA or DLR et al) had been performed on them. We added more detailed information about these kernel related TFs into line 344-345 of our revised manuscript.*

7) Overall, I would like to see the explanation provided in response to reviewer to be included in the main manuscript to be clear for the audience. For example, the reference about the 101 functional genes in kernel.

*Reply:* *Thank you very much again. According to your suggestions, we added more explanation that was provided in response into the main manuscript, including the reference about the functional genes in kernel (line 347, reference 27 and 29-90).*

8) As a community resource, how does this network compare to previously published maize networks from big data analysis (like huang et al plant physiology and Walley et al science)?

*Reply:* *Thank you for your question on this. We compared our Union GRN to the GRNs constructed by Huang et al (PMID: 29879919) and Walley et al (PMID: 27540173). These previous GRNs were constructed using maize V3 version genes, while our GRNs were constructed using V4 version genes. Therefore, we transformed the gene IDs of our GRN, and gene IDs of most* *regulatory pairs (83.65%) can be transformed into V3 version, successfully (Fig S24A). These regulatory pairs (83.65%) with V3 ID were compared to the merged GRN (four GRNs including leaf, root, SAM and seed, each with top 1 million edges) constructed by Huang et al (Fig S24B) and merged GRN (seven GRNs, each with top 1 million edges) constructed by Walley et al (Fig S24C). The overlapped GRNs accounted for 8.79% and 36.48%, respectively. The coincidence rate might be affected by different tissues, different regulator (TFs) set and target gene set during GRN construction given the dramatic dynamic landscape of TFs across different tissues or development stages. We added these comparisons into* ***Discussion*** *of our revised manuscript in line 599-605, and Additional file 1: Fig. S24.*

Fig. S24 Comparisons of Union GRN in our study and the GRNs in previous studies.

A. Genes ID in most predicted regulatory pairs (83.65%) in Union GRN can be transformed into V3 version. B. Comparison between the regulatory pairs with transformed V3 ID and the merged GRN constructed by Huang et al, the merged GRN was composed of four GRNs (leaf, root, SAM and seed GRNs, each with top 1 million edges). C. Comparison between the regulatory pairs with transformed V3 ID and the merged GRN constructed by Walley et al, the merged GRN was composed of seven GRNs (each with top 1 million edges).

Minor: Figure 2A: label the columns better

*Reply:* *Thank you for this suggestion, we have changed this figure in the new manuscript.*

Line 372 “suggesting that target prediction using GRNs is highly accurate. “this is an overstatement based on functional enrichment.

*Reply:* *Thank you for pointing this out, we have changed the sentence into “suggesting that target prediction using GRNs is* ***relatively*** *accurate” in line 405.*

**Reviewer #2:** The revised ms addressed most of my questions. I only have several minor points want to discuss.

*Reply:* *Really appreciate your careful reading, evaluation, and positive comments on our manuscript. We revised our manuscript according to your suggestions. Please see our point-by-point response below.*

1. In lines 564 and 567, the authors mentioned pmTGN. However, this term has not been introduced in the results part. Please check.

*Reply:* *Thank you very much for your pointing-out on this. The pmGRN was constructed in a previous study (Walley et al, Science, 2016, PMID: 27540173). We added more description about this in line 609 of our revised manuscript.*

2. Since TFs from translatome regulate the transcription of genes, it is reasonable that TmGRN outperforms mmGRN in recapitulating ChIP-seq networks. Does TTGRN also outperform mmGRN?

*Reply:* *Thank you for your helpful comments. According to our observation in Fig.3 and FigS9-S11, the TTGRN also showed better performance than the mmGRN in most cases. Additionally, although TTGRN and mmGRN showed comparable power in recapitulation of ChIP-seq networks, mmGRN is better than TTGRN in more cases. We added more comprehensive comparisons between TTGRN, TmGRN and mmGRN in our revised manuscript according to your suggestions.*

**Third round of review**

**Reviewer 1**

For point-to-point response #1: Please add a discussion on the potential problem caused by limited number of input gene expression / translatome samples on the construction of gene network, e.g. “the curse of dimensionality” when the number of genes (and the predictions) is greatly higher than the number of gene expression samples, and how this may affect the network presented here.

For point-to-point response #4:The description seems to be in lines 204-210 in the reviewer version pdf. Please consider further clarifying the statement in text by using intersect/overlap and union/merge and avoid using “integration” because it can be ambiguous. The explanation in the response to reviewer seems more accurate than the description in the text.

**Authors Response**

**Point-by-point responses to the reviewers’ comments:**

Reviewer #1:

1. For point-to-point response #1: Please add a discussion on the potential problem caused by limited number of input gene expression / translatome samples on the construction of gene network, e.g. “the curse of dimensionality” when the number of genes (and the predictions) is greatly higher than the number of gene expression samples, and how this may affect the network presented here.

*Reply: Thank you for the helpful suggestion, we had added a discussion about this in the section Discussion.*

*The sentences of added discussion are “In general, networks constructed using limited number of input samples tend to show lower performance and introduce more false positives. With a limited number of samples, networks tend to have lower weight values and reduced quality of prediction, particularly when more input genes are used during network construction. As an alleviation measure, the integration of multiple omics datasets greatly improves the predictive power of GRNs, and increasing sample size also has a positive effect on network performance [12, 18].”*

2. For point-to-point response #4:The description seems to be in lines 204-210 in the reviewer version pdf. Please consider further clarifying the statement in text by using intersect/overlap and union/merge and avoid using “integration” because it can be ambiguous. The explanation in the response to reviewer seems more accurate than the description in the text.

*Reply: Thank you for the helpful suggestion. We had clarified the statement in all text by using “overlap and union” and avoid using “integration” in our newly revised manuscript. As mentioned in our previous manuscript, we used union GRN for all the subsequential analyses.*
